# Silica–Biomacromolecule Interactions: Toward a Mechanistic Understanding of Silicification

**DOI:** 10.1021/acs.biomac.4c00674

**Published:** 2024-10-09

**Authors:** Christina A. McCutchin, Kevin J. Edgar, Chun-Long Chen, Patricia M. Dove

**Affiliations:** † Department of Chemistry, Virginia Tech, Blacksburg, Virginia 24061, United States; ‡ Department of Sustainable Biomaterials, Virginia Tech, Blacksburg, Virginia 24061, United States; § Macromolecules Innovation Institute, Virginia Tech, Blacksburg, Virginia 24061, United States; ∥ Physical Sciences Division, Pacific Northwest National Laboratory, Richland, Washington 99354, United States; ⊥ Department of Chemical Engineering, University of Washington, Seattle, Washington 98195, United States; # Department of Geosciences, Virginia Tech, Blacksburg, Virginia 24061, United States

## Abstract

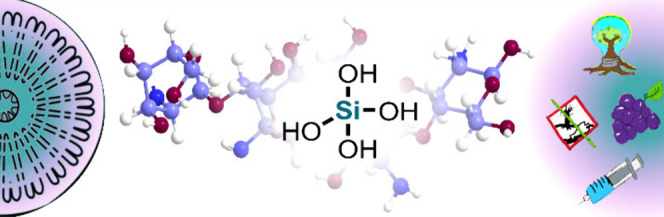

Silica–organic composites are receiving renewed attention for their versatility and environmentally benign compositions. Of particular interest is how macromolecules interact with aqueous silica to produce functional materials that confer remarkable physical properties to living organisms. This Review first examines silicification in organisms and the biomacromolecule properties proposed to modulate these reactions. We then highlight findings from silicification studies organized by major classes of biomacromolecules. Most investigations are qualitative, using disparate experimental and analytical methods and minimally characterized materials. Many findings are contradictory and, altogether, demonstrate that a consistent picture of biomacromolecule–Si interactions has not emerged. However, the collective evidence shows that functional groups, rather than molecular classes, are key to understanding macromolecule controls on mineralization. With recent advances in biopolymer chemistry, there are new opportunities for hypothesis-based studies that use quantitative experimental methods to decipher how macromolecule functional group chemistry and configuration influence thermodynamic and kinetic barriers to silicification. Harnessing the principles of silica–macromolecule interactions holds promise for biocomposites with specialized applications from biomedical and clean energy industries to other material-dependent industries.

## 1 Introduction

Diverse marine and terrestrial organisms have developed the ability to form inorganic–biopolymer composites using processes that are broadly known as biomineralization. The resulting amorphous and crystalline minerals are intimately associated with macromolecules of the organic matrix and exhibit properties that are quite unlike those of their abiotically formed counterparts. For example, these biomineral products often present greater fracture toughness, flexibility, and structural order on multiple length scales. These properties, combined with sometimes unusual morphologies, confer ecological or metabolic advantages to the organism. Skeletal support is a common biomineral function, but organisms can produce a diverse array of sophisticated biological materials that can serve as filters, grinders, light harvesters, and gravity and magnetic field sensors.^1−5^ Most biominerals comprise polymorphs of calcium carbonate, calcium phosphate, or silica, but the more than 60 crystalline and amorphous phases identified to date also include oxides, hydroxides, and sulfates.^1^

Structural biologists have made great strides in establishing the configurations of the “privileged” cellular spaces where biomineralization occurs.^2,6−9^ The cellular machinery contained therein determines the composition of the organic matrix (OM), the macromolecular mixture of proteins, polysaccharides, and lipids that comprise these spaces as well as drive life processes, including biomineral formation.^2,10−12^ Thus, the OM and the composition and conformation of macromolecules associated with sites of mineral formation are subjects of considerable attention from the biomineralization community.

This Review focuses on silica biomineralization and the roles of biopolymers in forming biosilica composite materials. Significant advances in understanding the cellular environment where biosilica is formed, combined with recent advances in glycomaterials research, present opportunities to finally establish the physical basis for the role of macromolecules in biosilicification. First, we highlight the structural and chemical characteristics of relevant aqueous silica chemistry as background before discussing more complex biologically-based silica chemistry. We then highlight recent studies that show the nature of local settings where biosilicification occurs with a focus on the OM and associated polysaccharides, proteins, peptides, and polyamines. Most investigations are highly qualitative, and the materials have not been well-characterized. The current literature illuminates the many challenges of accurately characterizing structurally complex proteins and glycomaterials. Collectively, they also show that our mechanistic understanding of how macromolecules promote (and inhibit) silica condensation continues to be limited.

The available evidence from studies of the organic matrix associated with natural biosilica indicates the overarching influence of the functional group identity on mineralization. Recurring chemical architectures include high charge density, with amines as the major cations and phosphates or carboxylates as the major anions. Cooperative interactions between opposite charges are suggested to play a major role in silicification, along with other molecular interactions, such as hydrogen bonding.

In the second part of this Review, we suggest that recent advances in biopolymer synthesis present an opportunity to design hypothesis-based studies that determine the thermodynamics and kinetics of macromolecule controls on mineralization. Of particular interest are derivatives of chitin, a polysaccharide that is widely found in association with biosilicas. Deacetylating chitin to chitosan yields a versatile material that can be tailored into a remarkable array of compositions to conduct systematic and quantitative studies of the kinetic and thermodynamic drivers of silicification. The opportunities therein open the way to building conceptual and computational models of macromolecular regulation of silicification. Harnessing the roles of these functional groups, as individual species or through cooperative interactions, is fundamental to understanding natural silicifiers and potentially transformational for synthetically developing novel silica-based biomaterials for industrial applications.

### 1.1 Overview: Silica Biominerals

**Figure 1:**
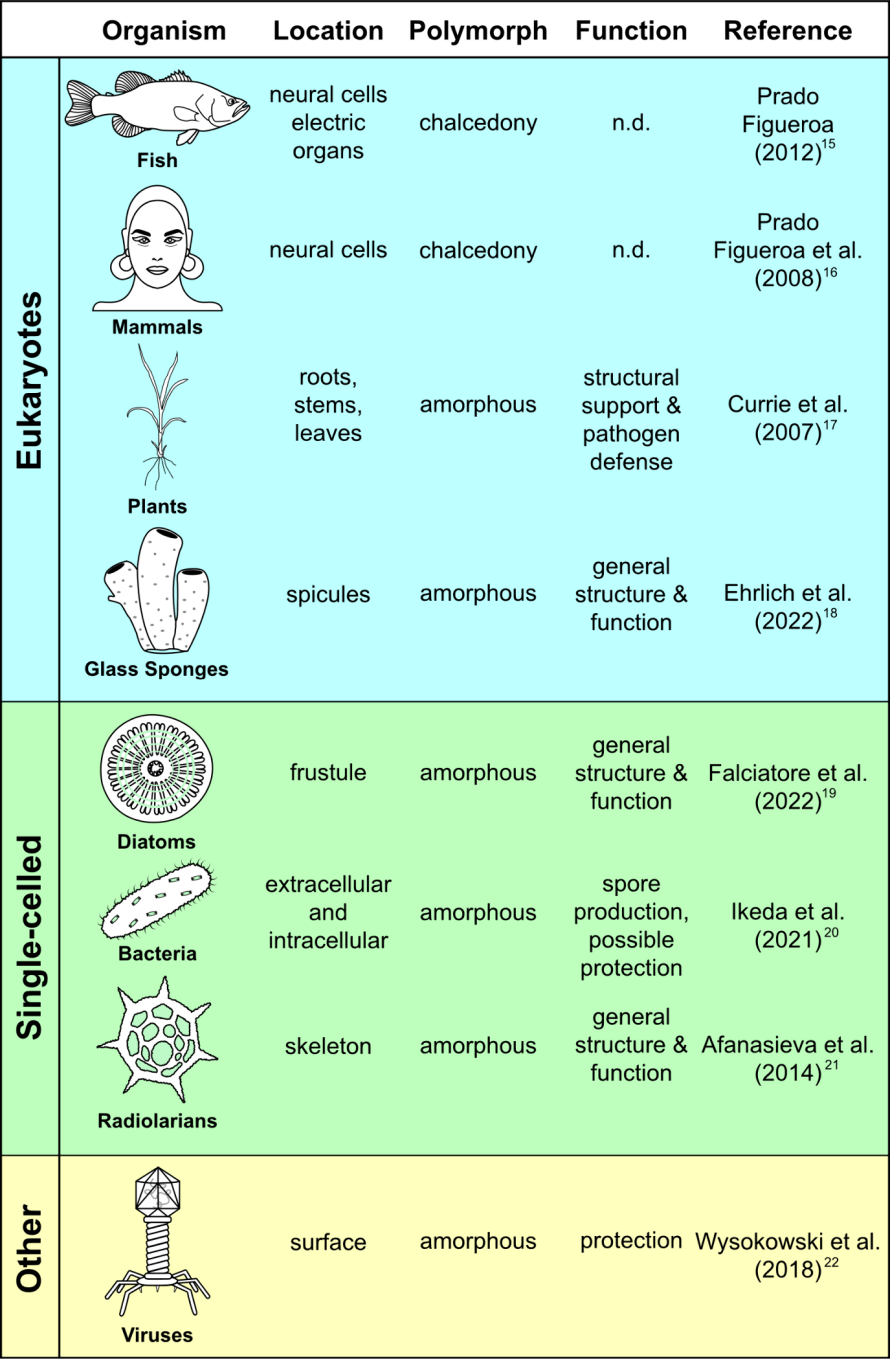
Diverse organisms produce biosilica as organic-SiO_2_ composites.^15−22^ Most structures serve protective or structural functions. “n.d.” indicates “not determined”.

**Figure 2:**
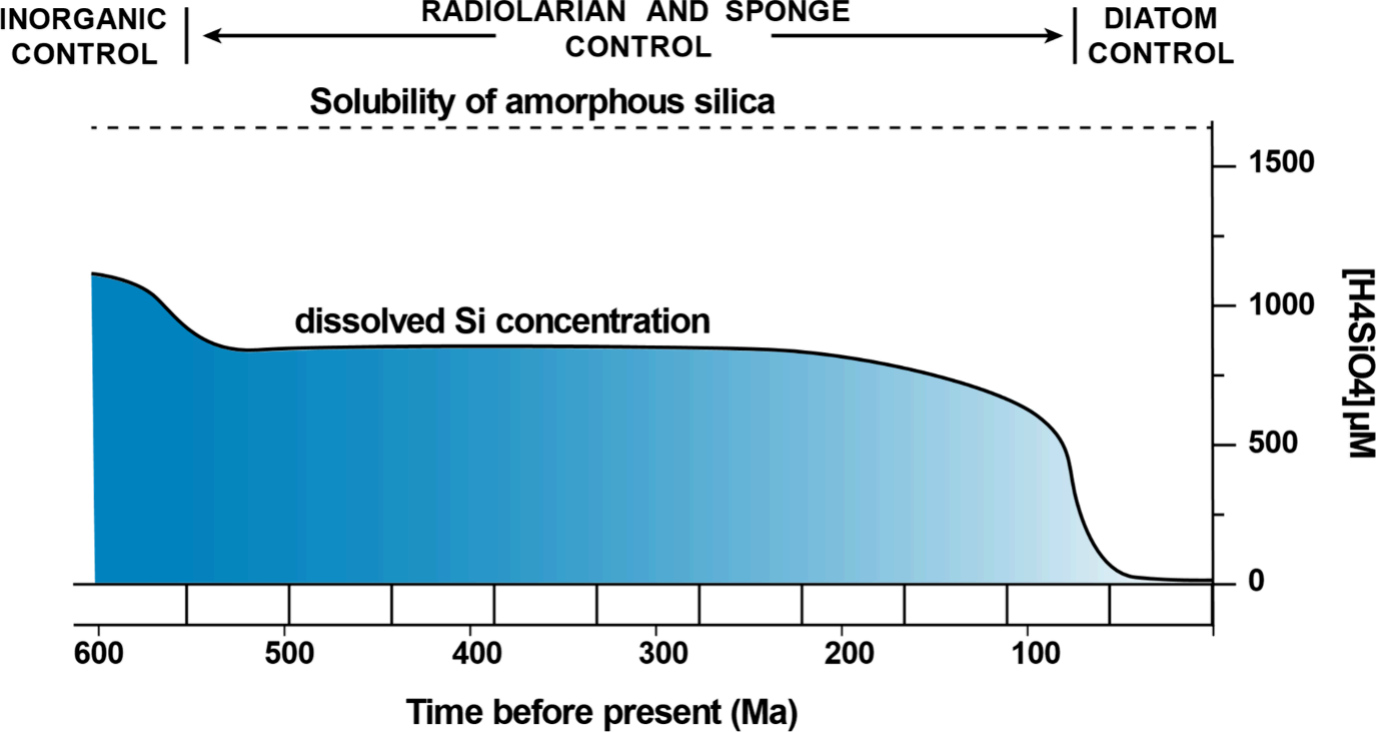
Average silica concentration in the ocean shows that the early ocean was near equilibrium with respect to amorphous silica. Silica levels began to decline and then plateaued with the evolution of radiolarians and glass sponges (550–200 Ma). Upon the emergence of diatoms (at ∼75 Ma), silica levels decreased sharply to present-day levels (after Conley et al., 2017).^13^

In earth systems, silica biominerals are produced by an astonishing diversity of organisms–from viruses and bacteria to plants and mammals (Figure 1). Most silicifiers live in marine environments, and three major groups have appeared over geologic time (Figure 2). Radiolarians and “glass” sponges were the first to emerge at a time when oceans contained more than 1,000 μmol of total H_4_SiO_4_° (∼550 Ma). With the emergence of diatoms (∼75 Ma), which sequester H_4_SiO_4_° to produce biosilica frustules, aqueous silica levels declined sharply (Figure 2).^13,14^

Diatoms dominate the biogeochemical cycling of silicon in modern oceans and annually produce gigatons of biosilica and organic carbon, as well as a significant portion of the oxygen that we breathe.^23^ Indeed, the ecological success of this major class of photosynthesizers over geological time led to a dramatic decrease in ocean water Si concentrations to the ≈31 μM H_4_SiO_4_° that is observed today (Figure 2).^13,24,25^ This continued ecological success is a critical component in the global biogeochemical system and is thus of great interest to climate researchers. The diatom’s mineralized structure, or frustule, exhibits exquisite detail that is reproduced over generations by the biochemical machinery that directs the species-specific morphological details with astonishing fidelity (see Section 3.1).

### 1.2 Overview: Materials Applications

**Figure 3:**
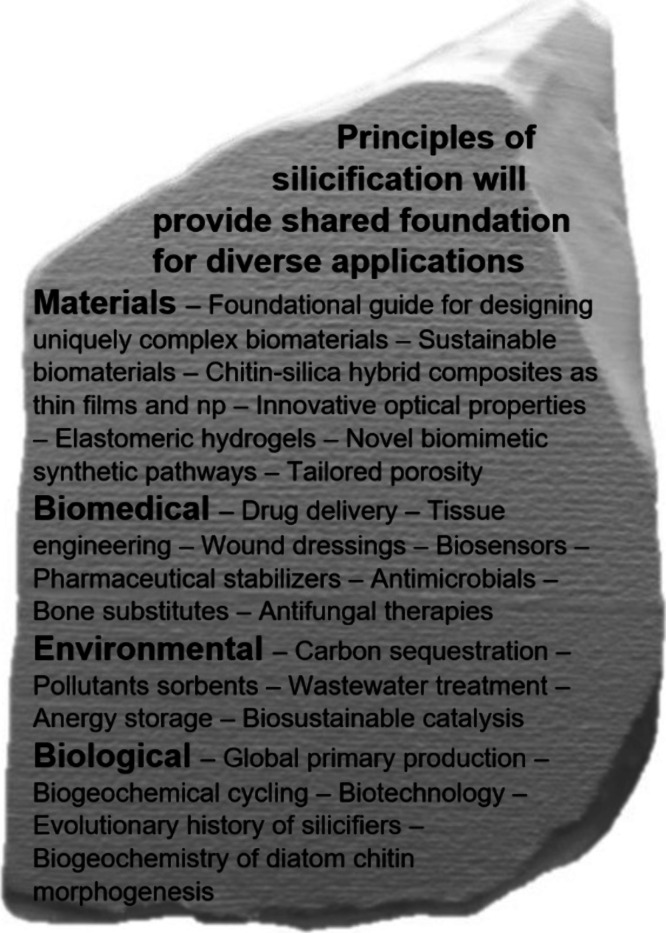
An understanding of silicification processes holds promise for diverse translational opportunities as illustrated by this “Rosetta Stone” of potential applications.

As an environmentally benign and relatively inexpensive material, silica is increasingly being incorporated into a variety of applications (Figure 3). However, the traditional process of industrial silica production, i.e., the Stöber process, is not environmentally benign. The Stöber approach has negative environmental impacts through high energy demands, caustic solvents (e.g., ammonium hydroxide), and petrochemical-based surfactants (toxic to aquatic life).^26^ The severe conditions of these synthetic processes contrast with silicification by marine organisms that produce silica materials at ambient temperatures, with little more than seawater and associated macromolecules. The silica produced by these marine organisms in turn confers hardness, microbial resistance, attrition resistance, and porosity to bioorganic materials such as peptides, proteins, and polysaccharides.^27^ Given these marked differences in natural and industrial pathways to silicification, it is apparent why efforts to harness the ability to sustainably produce tailored, morphologically hierarchical structures will grow as a frontier area of investigation.^26^ The many applications reiterate the transformational potential of establishing the physical basis for silicification and the expanse of translational opportunities for new material development (Figure 3).

## 2 Background to Structure and Chemistry of Silica

To explore how silicification is modulated by an OM or synthetic biopolymer system, we begin with the basic structure and chemistry of silica in aqueous environments. Natural biosilicas are formed at ambient or low temperatures and moderate pressures, where monosilicic acid, H_4_SiO_4_°(aq), and its deprotonated counterpart, H_3_SiO_4_^–^ (aq), are the monomeric forms found in an aqueous solution. These species are rapidly interchangeable by the acid–base reaction:^31^
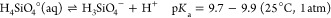
1

Subsequent reactions to polymerize silica are represented by the general condensation reaction:^31^
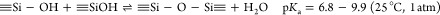
2

For a comprehensive discussion of silica formation, see Iler, 1979, and Belton, 2012.^32,33^

### 2.1 Silicification in Simple Inorganic Systems

The *inorganic* process of silicification is an S_N_2-like polycondensation reaction (that releases a water molecule as the coproduct) whereby the rate-determining step is the nucleophilic attack of H_3_SiO_4_^–^.^33^ Monosilicic acid is a high-energy, unstable monomer that undergoes autopolycondensation at neutral pH and room temperature above a concentration of ≈2 mM.^33−35^ Research suggests that, at most pH values, aside from extremely low pH, silica polymerization or condensation (the terms are used interchangeably in most literature; see Table 1) occurs until the reaction reaches equilibrium.^33^

**Table 1:** Summary of the Silica Morphologies and Definitions Used in This Discussion

Term	Definition
Silicification	Combination of reactions 1 and 2, also referred to as silicic acid polymerization or condensation
Silicic acid	General term for monosilicic acid and its dimers, trimers, and oligomers
Silicate	General term for ionized/deprotonated silicic acid
Silica sol	Aqueous solution of colloidal particles [held together by electrostatic and van der Waals forces]^36^ with sizes 1–1000 nm^37^
Silica gel	Sol particles with siloxane bonds at points of contact (linked together into 3-D networks);^32^ Interconnected rigid structure of polymeric chains with microsized pores^36^
Colloidal silica	Dispersions or sols of discrete particles of amorphous silica, large enough to be stable^32^
Colloidal particles	3-D polymers of silica^32^
Particles	Spherical or nonspherical structures with nm-scale diameters
Aggregates	Unstructured fusions of particles or sols

**Figure 4:**
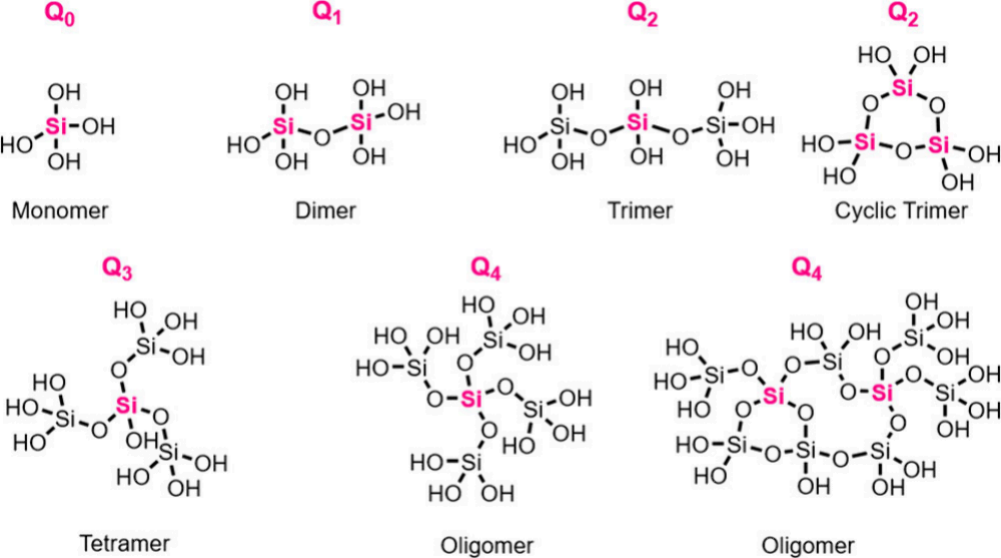
Nomenclature of silicic acid species that can occur in solution (monomer to oligomer). The Q_*n*_ convention refers to the number of Si units attached (through the oxygen) to an adjacent Si atom.^33^ Thus, Q_*n*_ refers to Si(OSi)_*n*_(OH)_4–*n*_ where *n* equals 0, 1, 2, 3, or 4. Q_*n*_ notation is represented by the silicon atom in pink. The Q_*n*_ convention is widely used in ^29^Si NMR.

**Figure 5:**
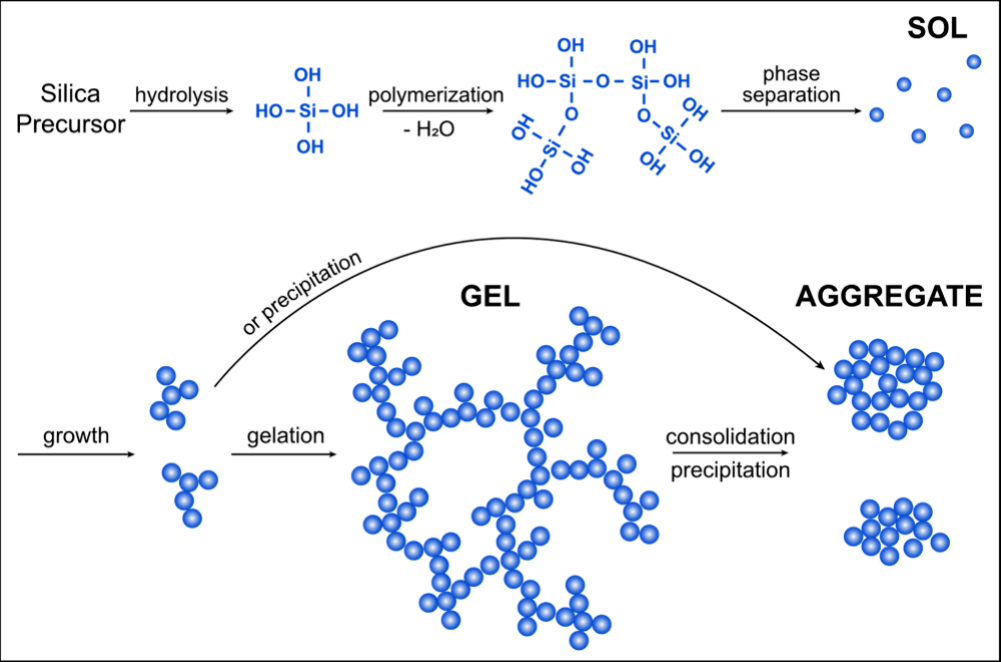
A simple representation of the common nomenclature for the formation of higher order silica structures in inorganic systems (sols, gels, and aggregates) provides a visualization for discussions herein (after Wilhelm and Kind, 2015).^213^

The p*K*_a_ of oligomers is structure-dependent, can vary from 9.5 and 10.7, and progressively decreases with higher degrees of polymerization (DP). For example, surface silanols have a p*K*_a_ of 6.8 when bound to aggregates with a diameter of ≈1 nm (Reaction 2).^33^ Although the p*K*_a_ of silicic acid is relatively high, at neutral pH (Reaction 1) ≈0.18% of the total H_4_SiO_4_° is ionized and can promote oligomerization at room temperature.^33^ Monomers react with one another to form dimers, trimers, and then higher oligomers and polymers (Figure 4). Oligomers that consist of greater than two monomers often cyclize, forming structures such as cubic octamers and prismatic hexamers.^33^ Si–O–Si bonds can have a wide range of angles (135–160°) and bond lengths (1.58–1.62 Å), which allows for a wide array of structures.^38^ To our knowledge, the increase in energetic stability that occurs with the formation of cyclic and prismatic structures has never been quantified; however, ion–solvent interaction enthalpies of ionized monosilicic acid were calculated by Yang et al.^39^ The reversibility of silicification means that thermodynamic and kinetic factors must be favorable to form silica. As oligomers continue to polymerize, larger particles and amorphous aggregates exhibit variable length scale (and degree) of structural order (Figure 5). These larger particles continue to grow at the expense of the smaller particles, which have a higher ratio of surface energy to bulk material properties, in a process generally known as Ostwald Ripening.^26,33^

Silicification is complex and depends upon total H_4_SiO_4_ concentration, pH, types and concentrations of salts/ions, ionic strength, pressure, and the presence of other chemical species such as biomolecules or inorganic impurities.^32^ All of these factors influence the final characteristics of natural and synthetic silicas. The stability of the Q_*n*_ species also is dependent upon solution conditions. Q_*n*_ nomenclature describes Si(OSi)_*n*_(OH)_4–*n*_ moieties (where *n* = 0, 1, 2, 3, or 4, Figure 4) and is commonly used in ^29^Si NMR studies of silica formation where speciation can be resolved. For example, starting with sodium silicate solutions (75 mM with respect to SiO_2_) and very high pH (>12), Q_0_ and Q_1_ species are stable and dominant in solution.^40^ Oligomers and particles are more anionic in circumneutral pH solutions than monomeric species due to their decreasing p*K*_a_ values as previously discussed.^33^ In contrast, at low pH (<2), silicic acid species that form from sodium silicate (75 mM with respect to SiO_2_) are stable in the form Q_0_, Q_1_, Q_2_, Q_3_, and Q_4_.^40^ During the silica nucleation process, oligomers form thermodynamically stable silica nuclei, thought to be on the order of 1–2 nm in diameter, before rapid polymerization forms higher order structures.^41^ pH has significant influence upon the higher order morphology of silica as well. Near-neutral pH and low ionic strength conditions favor the formation of a **silica sol**, or solution of colloidal particles (Figure 5, Table 1).^34^ These particles have a diameter of 1–1000 nm and are held together by electrostatic and van der Waals forces.^36^ At lower pH (<7), a **silica gel** (Figure 5) is formed when a 3-D network of siloxane bonds forms between sol particles.^32^ The resulting interconnected structure of polymeric chains contains microsized pores.^36^ In high pH solutions, sol particles further combine to form **silica aggregates** (Figure 5).

In *in vitro* studies, silicic acid precursors have large and varying effects on the silicification studies. Precursors such as inorganic salts and organic acids are used in *in vitro* silicification studies due to the instability of monosilicic acid.^31^ Tetramethyl orthosilicate (TMOS) and tetraethyl orthosilicate (TEOS) precursors sometimes require the addition of methanol or ethanol to inhibit phase separation in aqueous systems.^29,30^ They also require acidification or alkalization of the solution to produce silicic acids, which then form a sol or gel, and they form a nonprecipitating gel above 25 wt %.^29^ The general reaction for hydrolysis of precursors is given by
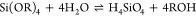
3

Salinity also has an important role in silica reactivity, particularly under conditions where silica species are charged. The combination of salts and charged species decreases the repulsion between silicic species, which leads to increased aggregation.^33^

### 2.2 Methods of Analyzing Silicification

For the purposes of this Review, it is useful to briefly consider the most widely used methods of silicification analysis and the types of information that they provide (Table 2). Two of the classical methods used to track silica formation include (1) the beta-silicomolybdate method (also known as the molybdate yellow method), which produces a yellow color, and (2) the molybdenum blue method, which produces a blue color.^32^ Both of these methods utilize molybdate to form complex monosilicic acid, which forms a color that is detectable via UV–vis spectroscopy. These robust methods have been used for several decades.

**Table 2:** Comparing and Contrasting Established Silicification Analytical Methods

Methods	What It Measures	How It Measures	Quantitative?	Data Gained?	Destructive?	Cons	Pros	Reference
^29^Si NMR	Magnetic environments of Si nuclei	Resonance signal released from magnetic pulse to nuclei	Yes	Relative concentration and identification of silicic acid species	No	Not cost-effective; can be time-consuming	Widely available	Zerda et al. (1986)^53^
β-Silicomolybate or Molybdenum Blue	Molybdate-reactive silica	UV–vis spectroscopy	Yes	Concentration of species in solution	Yes	Instable color	Cost effective, rapid results	Iler (1979)^32^
ESI-MS	Mass to charge ratio	Ionized aerosolized solution separated by voltage	No	Size of species in solution	Yes	Noisy spectra; salt interferes with signal	Rapid results	Takahashi et al. (2015)^42^
SEM/TEM	Diameter and 2-D shapes of particles	Electron scattering	No	Visual morphology	Sometimes	No quantifiable data	Common technique	Belton et al. (2008)^49^
AFM	3-D shapes and sizes of particles	Tactile	Yes, for counting critical nuclei	Number of nuclei formed over time	No	Difficult to work with	Simple quantification of nuclei	Wallace et al. (2009)^51^
GC	Relative sizes of oligomeric or polymeric species	Volatile species separated through a stationary phase	No	General sizes and concentrations of species in solution	Yes	Trimethylsiylation process can obscure species	Cost effective; rapid results	Shimono et al. (1983)^44^

The beta-silicomolybdate assay is the most commonly used analytical method for determining dissolved silica concentration due to its versatility, cost-effectiveness, and accessible equipment requirements. In this assay, a yellow color is observed upon the complexation of a monosilicic acid molecule with MoO_4_^2–^ ions to form a Keggin structure, as demonstrated by Takahashi et al. in 2015 via ESI-mass-spec.^42^ The color takes time to develop as an equilibrium exists between depolymerization of small oligomers and monomeric complexation by the molybdate species.^32^ Another equilibrium exists in this system between the beta and alpha forms of the silicomolybdate structures.^32^ The beta structure degrades into the fainter yellow alpha structure. Iler in 1979 ensured the stability of the beta structure in this method by carefully tuning the concentrations of MoO_4_^2–^, acid, and base, causing the color to be relatively stable between 2 min and 2 h.^32^ The addition of alcohol also favors the beta form.^32^ Absorbance is typically measured at 410 nm.^32^ It is important to remember that no more than 2 mg of SiO_2_ should be added to 40 mL of molybdate reagent before the final volume is adjusted to 50 mL.^32^ Phosphate ions can interfere with this method, but this is minimized by introducing oxalic, citric, or tartaric acids.^32^

The molybdenum blue reaction is used for lower concentrations of silicic acid on the scale of only a few parts per million or when phosphates are present in solution.^32^ This is the reduced molybdate complex of the beta silicomolybdate method. With this method, only 1–20 mL of a sample containing 10–50 μg of SiO_2_ can be added.^32^ After 3 h, the absorbance is measured at 810 nm.^32^ Overall, the beta silicomolybdate method is more robust and efficient for most systems. Both molybdenum-based techniques provide little information about the species in solution. There has been a debate regarding which species (e.g., dimers, oligomers) degrade into the monomeric structures which are then encapsulated by these Mo-based complexes during color development.^31,41,43^ A full outline of these methods is provided by Iler.^32^

Historically, researchers have also used gas chromatography (GC) to assess silicic acid species in silicification. In 1983, Shimono et al. reacted silicic acid species that were formed under various concentrations and pH values with HDMS and *n*-propanol under acidic conditions.^44^ This effectively formed volatile trimethylsilyl species, which could be measured via GC. The group identified species by comparing the elution times to volatile molecules of similar molecular weights. This method effectively showed trends that were similar, but not identical, to those produced by the beta-silicomolybdate method.^44^ This analysis is limited by the reaction rate and potential destructive nature of trimethylsilylation as well as the presence of foreign, nonvolatile species in solution. In addition, a comparison to species of similar molecular weights may not give an accurate picture of the solution. Potentially, this method can be paired with mass spectrometry (GC-MS) to identify and quantify the species in solution more accurately. For more information, a review of spectrophotometry and GC for silicification is available by Tarutani (1989).^45^

Mass spectrometry (MS) is a technique used to characterize molecular weights of molecules. There are many forms of this method with varying size and detection limitations. Classically, MS is used for molecules under a molecular weight of 1,000; however, matrix-assisted laser desorption/ionization (MALDI) was developed to analyze much higher molecular weights.^42^ Bussian et al. teased out the types of silicate oligomers present in a specific solution using MS and ^29^Si NMR.^46^ The team discovered that high concentrations of tetramethylammonium hydroxide (TMAOH) stabilize the cubooctameric double-four-membered ring silicate while tetraethylammonium hydroxide (TEAOH) stabilizes the double–three-membered ring silicate.^46^ Under ocean-inspired conditions, Tanaka et al. used fast atom bombardment mass spectrometry (FAB-MS) to understand silicate speciation and detected a handful of specific complexes in solution. They confirmed that many species exchanged H^+^ for the Na^+^ ion.^47^ The monomeric peak was not visible due to interference by salts in solution.^47^ FAB-MS is now outdated, and MALDI-TOF is more commonly used instead as it can identify somewhat higher molecular weights with high resolution. In 2020, Benhelal used MALDI to analyze silica.^48^ While the spectra they obtained did not have high resolution, the team was able to decipher that the repeating mass unit of 202 amu contains three silicon atoms, one oxygen, and six hydroxy groups.^48^

Applications of MS are generally limited for processes such as silicification analysis due to the complex spectra formed by the many ionized silicate species. For example, two molecules of the same molecular weight with similar ionization patterns are virtually indistinguishable. In addition, while MS technology continues to evolve, a majority of MS methods used in studying silicification are qualitative. Overall, more work is needed in this field to use MS as an effective tool for oligomeric silicate speciation.

Microscopy (SEM and TEM) is one of the most common techniques used today for the visual characterization of silica formation on the micro- to nanometer scale. SEM forms an image by detecting reflected electrons, whereas TEM detects transmitted electrons. These qualitative techniques are used to identify the morphology of the particles that form, their diameter(s), and possible internal structures. Generally, TEM provides higher magnification and resolution, while SEM can provide a larger field of view. Using SEM and TEM, Belton et al. found that silica formed in the presence of certain amines is composed of spherical, hollow nanoparticles.^49^ It is also difficult to distinguish possible contaminants in the system from purely visual systems. Only recently has this technology been developed into techniques such as electron tomography or 3-D SEM to provide three-dimensional information, which is helpful for providing a more complete understanding of the system.^50^

*In situ* atomic force microscopy (AFM) was utilized by Wallace et al. in 2009 to follow the formation of silica nanoparticles.^51^ The AFM method rasters a small tip over a surface to provide high resolution 3-D images of the changing surface structure in real time (<10 nm in the *XY* plane and <0.1 nm in the *Z* direction),^52^ unlike SEM or TEM which provide 2-D snapshots.^52^ Wallace et al. used this method to measure the rate of formation of stable silica nuclei to evaluate the interplay between kinetic and thermodynamic driving forces for nucleation.^51^ When performing AFM, it is important to ensure that the sample is adequately adhered to the substrate, preventing detachment during scanning.^52^ AFM applications can be limited by raster rate when several minutes per frame are required.^52^ However, fast AFM setups are now available and are increasingly used.^52^

Today, ^29^Si NMR is the leading technology used to analyze silicification. NMR is a qualitative and quantitative technique for characterizing molecular species. Both solid-state (MAS NMR) and liquid-state forms are available. As this Review highlights active silica formation in solution, we address solution NMR. The accuracy of silicic acid monomer concentration analyzed by ^29^Si NMR was found to be comparable with the silicomolybdate method according to Zerda et al. 1986.^53^^29^Si is a spin 1/2 nucleus with a natural abundance of 4.7% and a significant gyromagnetic ratio (γ) of −53.190 × 10^6^ rad × s^–1^T^–1^.^54^ While the values of these parameters might indicate that this nucleus is an easy candidate for NMR, the lengthy longitudinal and transverse relaxation times greatly decrease the ability to produce a significant signal-to-noise ratio. The common relaxation agent Cr(acac)_3_ has been used to overcome this challenge and has been shown to not interfere with the silicification process, but it is only an option in organic solutions. Low Si concentrations, such as those mimicking natural and biotic environments, also pose significant barriers to obtaining adequate signal-to-noise. Therefore, costly ^29^Si enrichment is often necessary to obtain strong silicification results. Meinhold et al. used ^29^Si enrichment to track rapid dimer formation,^55^ and Yang et al. rationalized the trends of thermodynamic constants based on monomer and dimer concentrations.^39^ More recently, Preari et al. used this technique to track monomer and dimer concentrations in the presence of a macromolecule to decipher trends.^101^ Montagna et al. used NMR and molecular dynamics simulations to make conclusions about electrostatic interactions between silica and polyamines.^56^ Bravo-Flores used NMR to suggest that Si–O–C bonds were formed in some solutions.^57^ Overall, this quantitative, versatile technique has great potential in understanding silicification.

## 3 Major Silicifiers

Biosilicifying organisms are found worldwide from deep ocean environments to terrestrial ecosystems. Here, we focus on three broad categories of biosilicifiers that provide considerable insight into biosilicification processes: diatoms, glass sponges, and plants.

### 3.1 Diatoms

**Figure 6:**
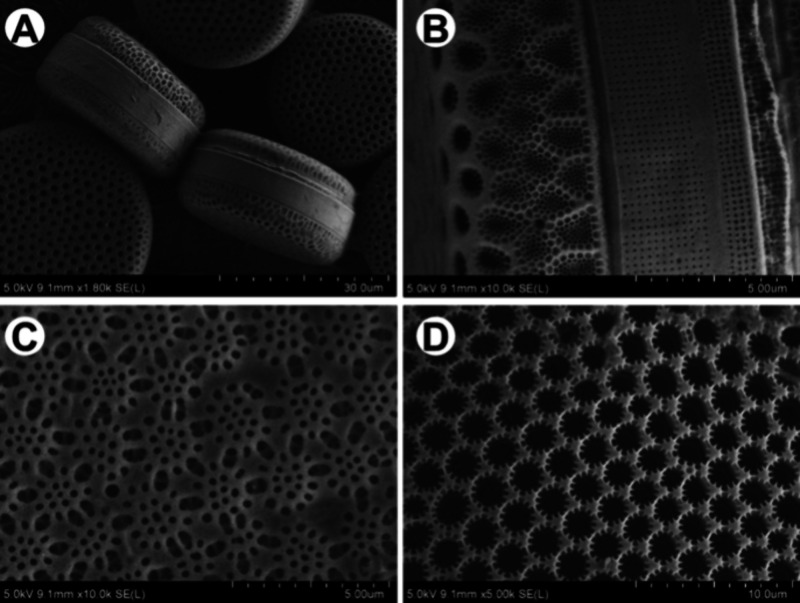
Hierarchical structures of diatoms including the (A) view of valve and girdle of *Endyctia* sp.; (B) central part of diatom internal frustule of *Endyctia* sp.; (C) outer side of valve of *Coscinodiscus* sp.; (D) inner side of valve of *Coscinodiscus* sp. Reprinted with permission under a Creative Commons Attribution 4.0 International License from ref (214). Copyright 2021 Springer Nature.

On modern Earth, there are >250,000 diatom species in freshwater, seawater, and soils.^22,58^ From oceans to freshwaters, diatoms are perhaps the most prevalent silicifiers and photosynthesizers (and are thus critical in carbon fixation; see Section 1.1).^59^ These single-celled organisms sequester and mineralize silicic acid into amorphous silica-based frustules that present stunning morphological and species-specific complexity (Figure 6). Diatom frustules comprise amorphous SiO_2_–OM biocomposites that exhibit high mechanical strength, interesting optical properties such as blue light absorption, and detailed hierarchical structures.^35,60,61^ These structures continue to inspire efforts to harness the biochemical mechanisms by which biomolecules direct silicification, especially within materials synthesis communities.

Efforts to understand the diatom silicification process led to the 1964 discovery of the silica deposition vesicle (SDV) via electron microscopy.^62,63^ Silicification has since been collectively viewed as an intracellular reaction.^62^ However, a 2021 article by Mayzel et al. presents evidence that diatom silicification occurs both extracellularly and intracellularly, showing that diatom research is still advancing.^64^

Diatoms sequester silica from natural waters in two ways: (1) passive diffusion through the cellular membrane at environmentally relevant concentrations and (2) use of specialized silicon transporters that are activated at low local concentrations.^34^ The concentration of intracellular silicic acid is documented above the 2 mM saturation limit, with an average range of ≈1 to 20 mM,^65^ suggesting there are yet-unidentified molecular controls that stabilize the acid and prevent polymerization until it is captured in the SDV.^34^ Silicification in the SDV is thought to occur in diatoms at a lower pH (∼5–6), as this pH range experimentally facilitates the formation of networks of silica structures similar to diatom frustules.^40,66^ After polymerization in the SDV, silica is released from the cell surface. While silica morphology of diatoms varies, diatoms most often produce gel networks of silica, SiO_2_(am).^34^

**Figure 7:**
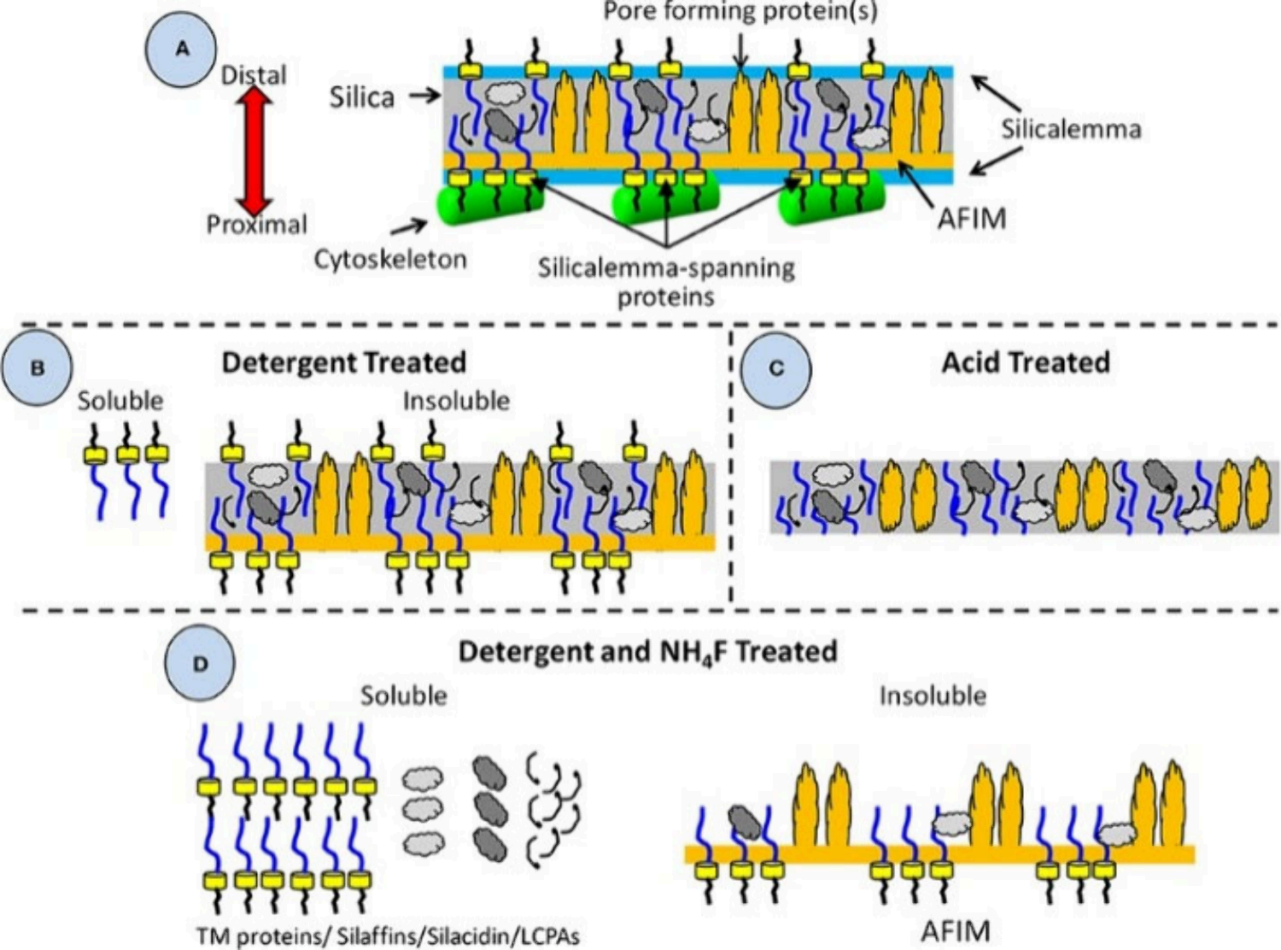
Schematic of diatom frustrule structure and methods for extracting organic molecules. (A) Frustule components and cytoskeleton. Silica is the midgray color surrounding the components, and some proteins are represented by the light and dark gray globules. (B) Detergent treatments remove cytoskeleton and silicalemma and most silicalemma TM proteins. (C) Acid treatment removes all organic material that is external to the silica. Some embedded materials are thought to remain, protected by the silica. (D) Frustules are extracted by both detergent and ammonium fluoride. Most organics are extracted, leaving the AFIM, which includes molecules such as polysaccharides. Reprinted with permission under a Creative Commons Attribution 4.0 International license from ref (34). Copyright 2018 Frontiers Media S.A.

To the best of our knowledge, the SDV has not been isolated, but many biochemical studies have investigated the properties of the organic matrix within the SDV that template and guide silicification. These include genomic studies, silica dissolution with multiple types of extraction (Figure 7), and *in vitro* studies (see Section 4).^34^

Overall, many macromolecules have been extracted or genetically identified from the diatom organic matrix including a variety of proteins, long chain polyamines (LCPAs), and polysaccharides. However, the entire molecular framework of the organic matrix remains unclear to this day, in part due to the rigorous and varied extraction methods required to analyze the vast array of silica-associated molecules.^19^ Detergents and ammonium fluoride are capable of extracting most proteins and LCPAs, but they still leave many molecules yet to be analyzed in the ammonium fluoride insoluble material (AFIM) (Figure 7). The following sections discuss the molecules that have been successfully extracted or genetically determined and analyzed with respect to diatom silicification.

#### 3.1.1 Key Diatom Proteins

**Figure 8:**
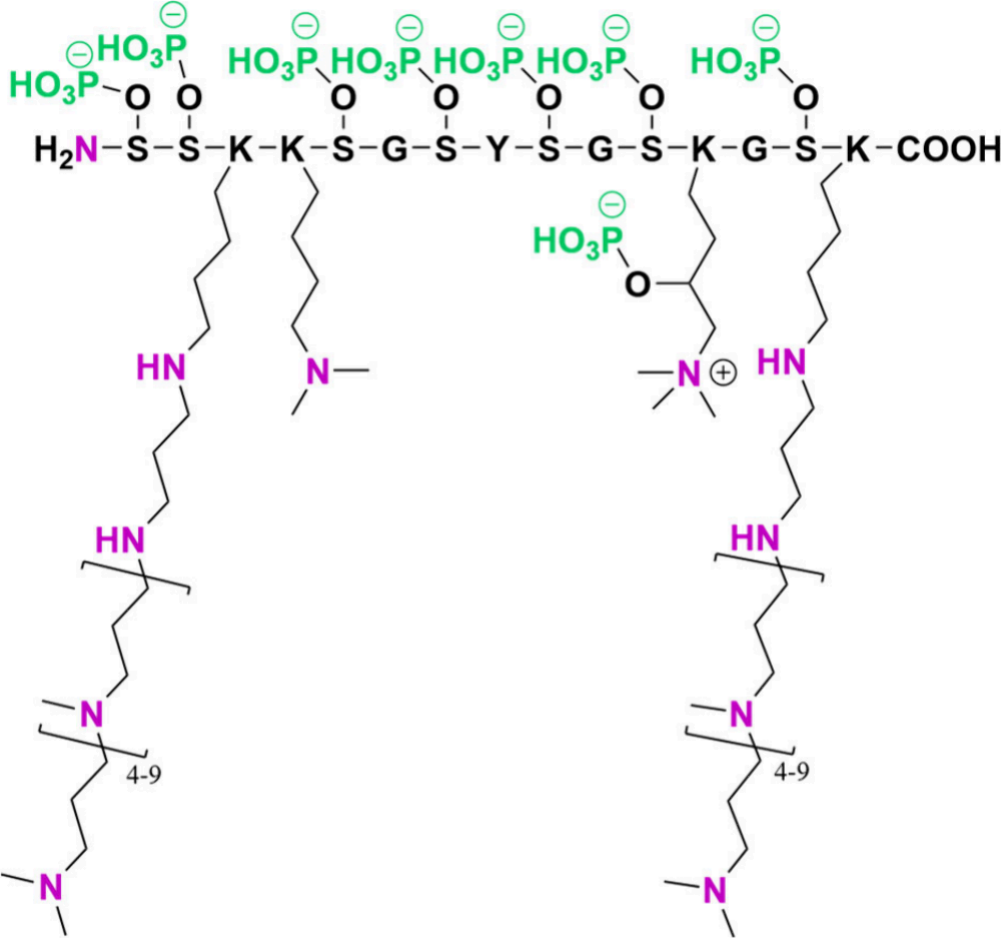
Diatom Silaffin-1A_1_ from *C. fusiformis*(68) shows backbone and post-translational zwitterionic functionalization: anionic phosphate groups (green) and cationic polyamines (nitrogen molecules in pink) (after Kröger et al., 2001).^215^

A variety of proteins are implicated in controlling silicification in the diatoms. Kröger et al. extracted a new family of proteins from a diatom frustule that they denoted *silaffins*.^67^ Six silaffins are known in diatoms, and each present post-translational modifications to form a zwitterionic motif (Figure 8).^19^ The lysine-bound LCPAs are the cationic groups, and the anionic groups are typically phosphorylated serine, threonine, or hydroxyproline groups or sulfated saccharides attached via *O*-glycosylation.^19^ Post-translational phosphorylation of silaffins was discovered after the use of the NH_4_F extraction method, as opposed to the earlier method that employed HF, which hydrolytically cleaved O–P bonds.^26,68^ This detection of phosphate groups bound to silaffins highlights the importance of carefully choosing extraction methods to study diatom OMs (Figure 7).

Silacidins make up another class of proteins extracted from frustules. These short (∼25 amino acid) proteins are highly anionic, being ∼60% phosphorylated.^19^ Silacidins strongly enhance silicification, and experiments that knockdown the genes corresponding to this protein significantly impact the size and silica content of diatoms.^19^ This activity of silacidins suggests they may play a role in silicic acid uptake or size maintenance in diatoms.^19^

Silicanins and silicalemma-associated proteins (SAPs) have been more recently elucidated than silaffins and silacidins.^19^ Silicanins are clustered in SDV and bind to LCPA molecules. Knockout experiments targeting one silicanin, Sin1, led to only subtle changes in the diatom frustule.^19^ In contrast, knockdown experiments of SAP1 and SAP3 caused visible deformities in the frustule.^34^ SAP1 and SAP3 proteins tagged in the C-terminal region with green fluorescent protein (GFP) tags were found to be associated with forming silica structures in diatoms, and SAP3 when tagged at the *N*-terminus appeared to be embedded in the silica.^34^ It was speculated that the serines of SAPs are phosphorylated (similarly to silaffins and silacidins) and interact with polyamine groups.^19^

These studies have advanced our understanding of how proteinaceous macromolecules are associated with sites of biosilicification; however, the exact functions of these protein families have yet to be elucidated.^69^ Sequencing of proteins across a variety of diatom species reveals a lack of homology and fails to pinpoint a key protein sequence that broadly controls diatom silicification.^69^ Overall, the silicification community lacks a strong understanding regarding protein roles in silicification.^69^ These studies also point to the possibility that post-translationally added functional groups or overall charge have a greater impact on controlling silicification than the specific amino acid sequences. For example, silaffin-1 and silaffin-2 show almost no sequential similarity to silaffin-3 or other silaffins. In addition, while silaffins are prevalent in *T. pseudonana*, silaffins appear to be absent in the *Coscinodiscus* genus of diatoms.^69^ This lack of homology between protein sequences, the absence of consistency of “key” proteins among silicifiers, and the gap in functional understanding highlights the need for studies that establish the roles of other functionalized macromolecules in silicification.

#### 3.1.2 Diatom Polyamines

**Figure 9:**
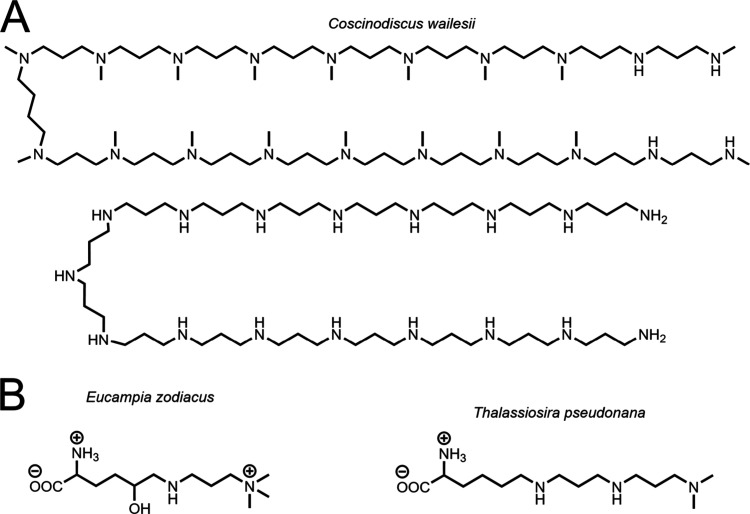
LCPA structures extracted from diatoms. (A) Free amines from a *C. wailesii* diatom frustule. LCPAs can be methylated or unmethylated. (B) LCPAs can be bound to a protein via a lysine residue (*E. zodiacus* and *T. pseudonana* polyamines shown), and polyamines can be charged or neutral (after Falciatore et al., 2022).^19^

Long chain polyamines (LCPAs) are macromolecular chains of amines often found covalently bound to silaffins and electrostatically associated with silacidins and silacateins in the SDV. These macromolecules are isolated from diatoms via HF or NH_4_F silica dissolution (Figure 7b,c). LCPAs are formed of 5–20 repeating units of linear oligo-propyleneimine (Figure 9A).^19^

**Figure 10:**
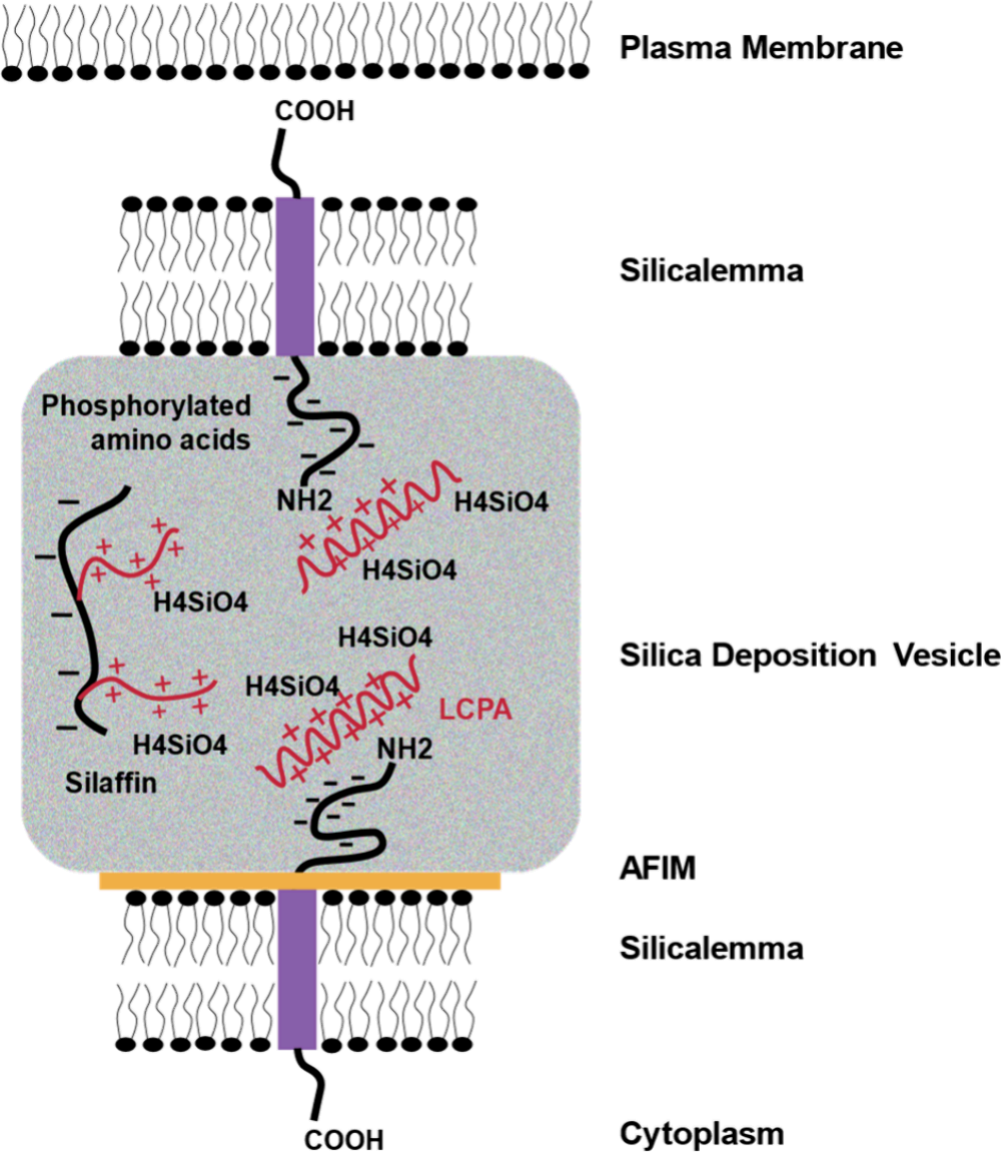
Simplified representation of the diatom silica deposition vesicle (SDV) lumen illustrates the interactions between LCPAs (red cationic chains) or silaffins (red and black zwitterionic moieties) with the phosphorylated serine residues (black anionic chains) of silicalemma-associated proteins (SAPs). Charged chains are suspected to interact with one another within the SDV (after Hildebrand et al., 2018).^34^

Covalent attachment of LCPAs to lysine groups on silaffins was confirmed recently by correlations between polyamine nitrogen and carbonyl carbons in heteronuclear 2-D NMR experiments (Figure 9B).^70^ The degree of methylation is another structural component of LCPAs and is species-dependent (Figure 9).^40^ The amine groups of these macromolecules impart a high degree of positive charge (note: p*K*_*a*_ values vary but many natural polyamines are highly charged below pH 7^71^), which accounts for the previously discussed associations with polyanionic phosphoproteins in the SDV (Figure 10). To our knowledge, *in vitro* experiments using mixtures of LCPA, polysaccharides, silaffins, and silacidins have yet to produce the morphology of the very porous, hierarchically structured diatom silica.^19^

**Figure 11:**
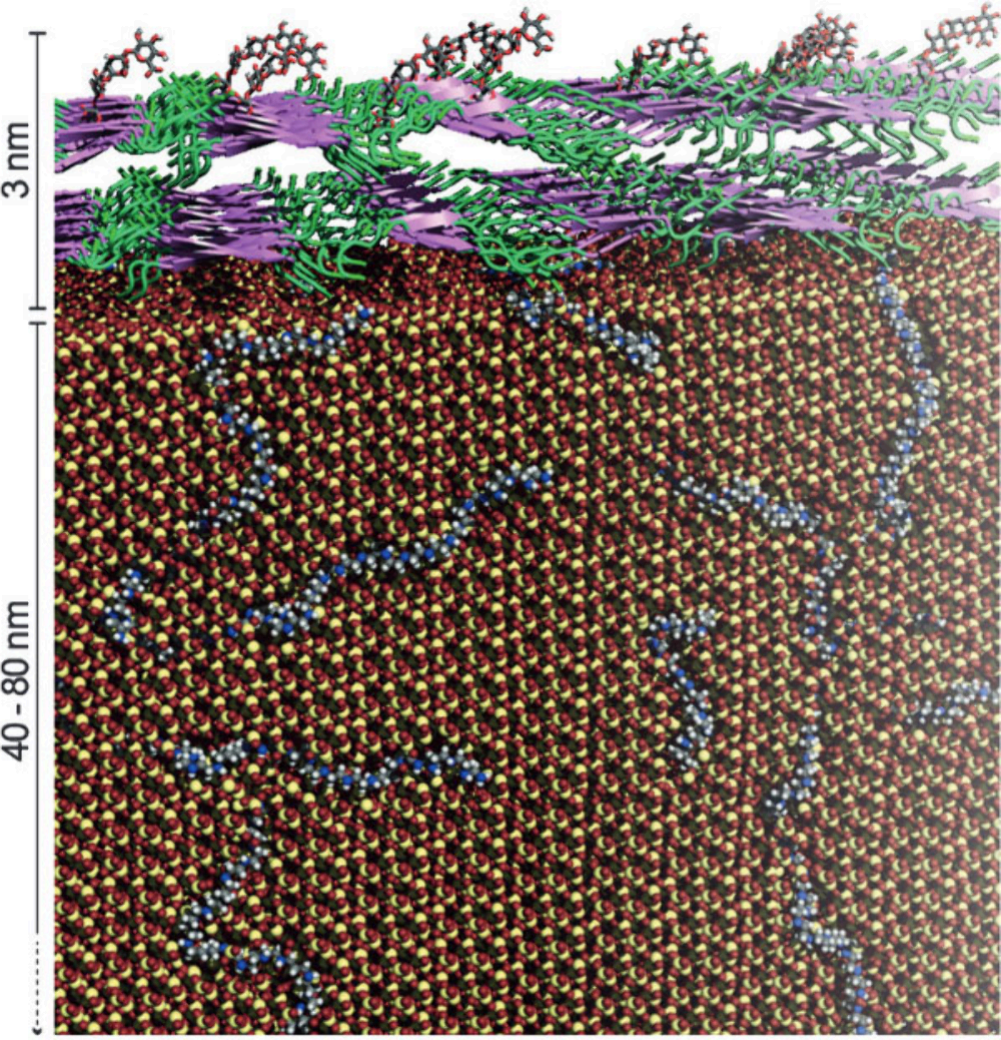
Molecular modeling of supramolecular architecture of biosilica (silica in red and yellow) with polyamines (blue, gray, and white) embedded in the 40–80 nm thick silica frustule. Proteins and carbohydrates (represented as purple and green) cover the silica as a 3 nm layer. Reproduced with permission from ref (72). Copyright 2015 John/Wiley & Sons, Inc.

In 2015, Jantschke et al. used analytical techniques (NMR, MS) as well as molecular dynamics simulations to decipher and model the relative locations of these macromolecules in the frustule and to elucidate their possible roles in silica formation.^72^ The team’s model found that native proteins are a mixture of random coil and β-strand conformations that form a 3 nm thick layer with polysaccharides that cover the silica phase (Figure 11).^72^ The modeling also predicted that polyamine structures are dispersed throughout the frustule.^72^ The findings prompt additional questions regarding the role of higher order structures of proteins and polysaccharides and how these macromolecules might affect silicification. This team’s research also illustrates the insights that come from combining analytical studies with computational modeling. While Jantschke et al. made great strides to further our understanding of this system, more studies must continue to identify the roles of each macromolecule in these complex systems.

#### 3.1.3 Diatom Polysaccharides

Although polysaccharides are prevalent in the biomineralized structures of diverse organisms, including diatom frustules, the role of this class of macromolecules in biomineralization is understudied in as they have been thought to function as inert scaffolding.^19,73−75^ The ubiquity of polysaccharides in biosilica stands in marked contrast to our lack of understanding regarding their roles in biosilicification.

**Figure 12:**
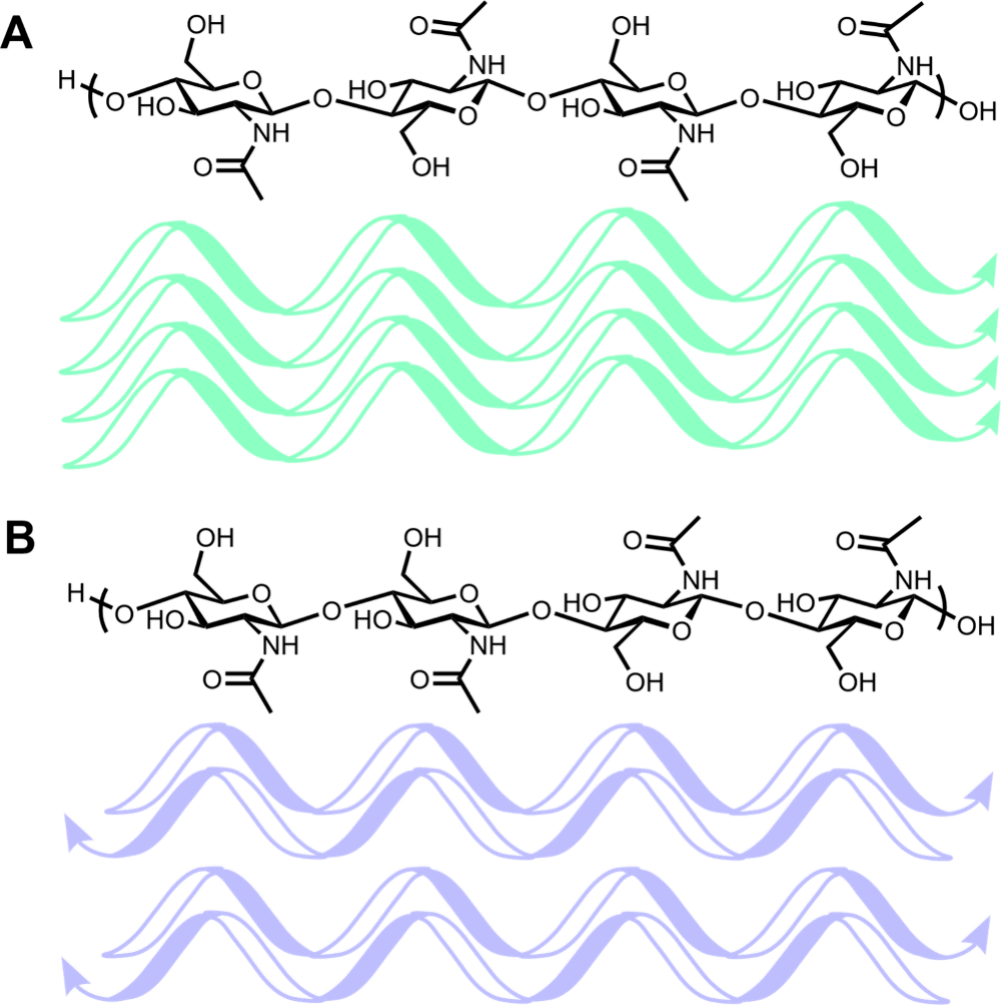
(A) Representation of α-chitin and higher order folding due to intermolecular interactions. Chains are aligned in the same orientation. (B) Representation of β-chitin and higher order folding intermolecular interactions. Chains are aligned in opposing orientations.

Chitin, consisting of *N*-acetylglucosamine units, is a major component of the frustule in diatoms. Chitin comprises two major forms; β-chitin has a parallel chain structure, making it more water-soluble than its α-chitin counterpart, which has antiparallel chains (Figure 12). β-Chitin is commonly found in and excreted from diatoms, possibly as a strategy to promote buoyancy.^76^ Kolbe et al. in 2021 used rotational-echo double-resonance (REDOR) NMR to demonstrate the presence of chitin in *Cyclotella cryptica* biosilica, noting that overall the polysaccharides of the organic matrix are not as well characterized as their protein counterparts.^75^ The observed *C. cryptica* extracted material is most likely composed of both α- and β-chitin.^75^ The AFIM (e.g., Figure 7D) is mostly chitin (a comparatively small portion is composed of proteins).^19^ In addition, chitin synthase genes have been identified widely among a variety of diatoms, suggesting this polysaccharide is a vital player in diatom function,^19,77^ but while chitin has been extracted and characterized in diatoms, the majority of frustule polysaccharides remain unexplored.^19^

Polyanionic diatomaceous polysaccharides have been isolated but have been less extensively characterized than chitin. Hedrich et al. isolated mannose-6-phosphate from *Stephanopyxis turris* biosilica which they believe was a hydrolyzed monomer from a larger phosphorylated polysaccharide.^78^ In addition, a linear poly-α-(1 → 3) mannan decorated with sulfate ester groups and β-d-glucuronic residues was isolated from the *Phaeodactylum tricornutum* cell wall and analyzed in 2017 by Le Costaouëc et al.^79^ It is plausible that the sulfate, phosphate, and glucuronate anionic groups associated with these polysaccharides influence silicification in diatoms. These recent analyses are impactful, as they highlight how much remains to be learned about biosilica synthesis regarding polysaccharides. For a comprehensive discussion of the macromolecules involved in diatom silicification, see Hildebrand, 2018,^34^ and Kröger’s *Biomolecules Involved in Frustule Biogenesis and Function* section of Falciatore et al.^19^

### 3.2 Sponges

**Figure 13:**
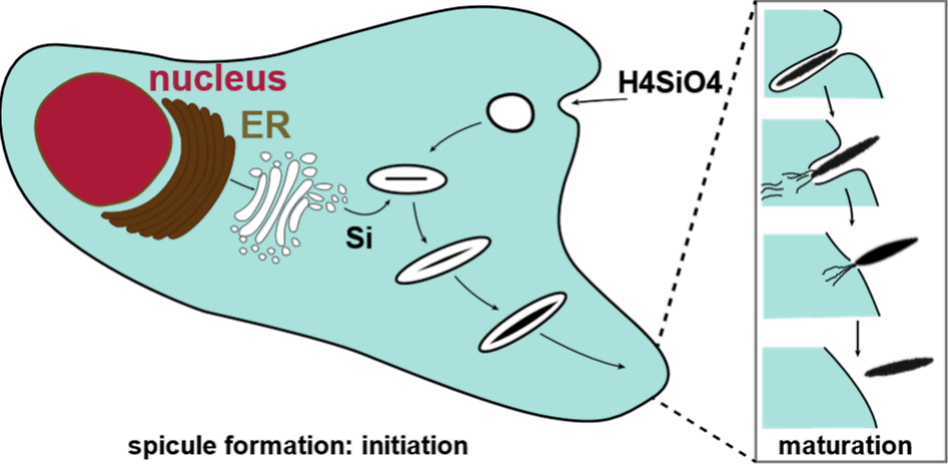
Simplified illustration of sponge spicule formation and extrusion from the sclerocyte. The process begins with the uptake of monosilicic acid by the cell. Subsequent condensation occurs in a vesicle before maturation and release as a biosilica spicule (after Müller et al., 2005.^216^

Sea sponges are the most significant, nonphotosynthetic biosilicifiers. Their global activity is estimated to result in a burial flux of ∼1.71 Tmol Si yr^–1^, thus comprising a considerable reservoir in the biological cycle of silica.^80^ Ninety-two percent of biomineralizing sponges produce silica, while ∼8% produce calcium carbonate skeletons.^81^ Sea sponges, such as those in the phylum *Porifera*, specifically in the classes *Hexactinellida* and *Demospongiae*, produce silica in the form of spicules, or skeletal building blocks, for structure, protection, and anchoring on the sea floor.^18^ Spicules represent 70–90% of the dry body weight of the sponge.^81^ The formation of spicules, like frustule formation in diatoms, is a dominantly intracellular process, forming within the sclerocyte vesicle prior to being extruded from the cell (Figure 13).^69^

Members of the *Demospongiae* class are found in marine and freshwater environments, from shallow water to ∼100 m deep. Due to their availability and simple laboratory cultivation, most silicification-based research on sponges is focused on the *Demospongiae* class. This class appears to utilize silicatein proteins to biosilicify.^82,69^*Hexactinellida* are found at water depths of several hundreds to thousands of meters and appear to use glassin protein as a catalyst for silicification.^82,69^ Overall, a variety of organic molecules are suggested to play a role in sponge silicification, from silicateins and other cathepsins, glassin, and collagen to polysaccharides such as chitin.^18^

Polysaccharides are present in both diatoms and glass sponges. In 2007, Ehrlich et al. discovered the presence of α-chitin in *Hexactinellida* sponges and hypothesized that this macromolecule must be a key templating agent for silica.^83^ In a recent study of *V. pourtalesii* sponges, chitin-binding activity was upregulated when higher silicon concentrations were present in the water.^82^ This was not the only upregulated gene or process; however, other molecules that were thought to be key to silicification, such as glassin 1 protein, were not as upregulated as expected.^82^ These findings are consistent with other studies showing that silicateins and glassin are not regulated in direct proportion to silicic acid concentrations.^82^ Owing to the assumption that polysaccharides provide inert scaffolding without influencing mineralization processes, there are few recent studies of how polysaccharides influence silicification. In contrast, many proteins such as silicatein, silicase, galectin, and collagen have been extensively studied for promotion of silicification in sponges.

Silicatein proteins are present in glass sponges and are highly studied as silicification promoters. Specifically, silicatein-α, -β, and -γ proteins have been researched, and these are phosphorylated similarly to many diatom proteins.^69^ Ehrlich et al. demonstrated that silicateins congregate around structurally supportive actin in the spicule.^18^ Sequence homology between proteins implicated in sponge silicification appears to be sparse, with only silicatein-1 and -2 having 50% homology.^69^ This lack of sequence homology suggests that the recurring functional groups bound post-translationally to these proteins may have a greater impact on silicification than the primary amino acid sequence. LCPAs are present in both glass sponges and diatoms. LCPAs in glass sponges have been found to be complexed with sulfates, suggesting a potential cooperative ion effect to promote silicification.^23^

In 2009, Wiens et al. published the discovery of silintaphin-1 in *Suberites domuncula*, which facilitated the formation of silica filaments *in vitro* in the presence of silicatein.^84^ Silintaphin-2 is smaller than silintaphin-1 and serves a similar purpose. Silintaphin-2 is composed of 20% negatively charged amino acids and 13% positively charged amino acids.^69^ Concentrated, highly hydrophilic regions are evident in both silintaphins.^84^ Glassin, another protein implicated in sponge spicule formation, comprises >30% histidine (often positively charged based on pH) and aspartic acid (often negatively charged near neutral pH).^85,86^ The amount of silica precipitated is directly proportional to the concentration of glassin in solution.^85^ The removal of these His and Asp rich water-soluble fractions from glassin deactivated this protein’s ability to promote silica precipitation.^85^ Nishi et al.^86^ suggested that (His-Asp)_5_ domains have a charge relay effect that drives silicification. Glassin has no significant sequence homology with other silicification-accelerating proteins, yet these proteins often share a zwitterionic motif.^85^

### 3.3 Plants

Silicification in plants is widespread. Plants are classified into three general categories in terms of their silica content as Silica-Accumulators, -Intermediates, and -Excluders (Table 3).^87^

**Table 3:** Plant Silicification Categories and Examples

Category	% of Dry Weight Si in Tissues	Example Species	Location in Example Species	Reference
Si-Accumulators	>4%	Rice [*Oryza sativa*]	mostly in husk and leaf blade	(17, 87−90)
		Sugar cane [*Saccharum officinarum L*.]	in the leaves, leaf sheaths, and root bands	
Si-Intermediates	1–4%	Oats [*Avena sativa L*.]	mostly in the glume, node, and lemma	(87, 88, 91, 92)
		Rye [*Secale cereale*]	mostly in the roots and leaves	
Si-Excluders	<1%	Tomatoes [*Lycopersicon esculentum* Mill.)	in leaves and shoots	(87, 93)

Both passive and active silicification mechanisms in plants have been proposed over the past decade.^94^ Plants take up silicon as H_4_SiO_4_° in soil waters.^94^ Exley suggests that plants are permeable to silicic acid, and thus, silicic acid uptake is passive via osmosis of aqueous solutions.^94^ However, evidence suggests that some species can also use a more active Si transportation process. For example, rice captures silicic acid much faster than water.^95^ In addition, Si transporters are found in several floral taxa, and many studies show silicification can benefit a variety of plant species.^93,96,97^ For example, increased Si often increases the plant’s resistance to biotic and abiotic stressors and increases mechanical strength.^98,87,99^

Plant cell walls are composed primarily of polysaccharides, and there are many cases in which plant cell walls are found to be impregnated with silica.^69^ Initially, a supersaturation of H_4_SiO_4_° (≈8 mM, ∼4–5× higher than the thermodynamic solubility in water at physiological pH) remains stabilized in the apoplast (extracellular space in plants) without precipitating. Like diatoms, the mechanism by which this stabilization occurs is yet unknown.^69,100^ This unidentified mechanism has been attributed to high negative pressures in the xylem or to hydrogen bonding of monosilicic acid with hydroxy groups, such as those in the cell wall polysaccharides.^94,100,101^

Silica mineralization and deposition in plants occur mainly in the apoplast and, to a lesser degree, in the symplast (interconnected cell membrane).^69,100^ In the leaves of some grasses, such as sorghum, specialized “silica cells” have been discovered. These cells secrete a specific protein known as “siliplant1” into the silicic acid-supersaturated apoplast. With the addition of this protein, silica immediately precipitates, and cells promote an ever-thickening silica deposit until they succumb to programmed cell death.^100,102^

Although there are few studies of the OM associated with sites of plant silicification, polysaccharides have been identified as strong candidates for influencing this reaction.^100^ Silica is associated with starch grains in potato tubers, where silica potentially hydrogen bonds with the sugar units.^100^ Hemicelluloses and callose, a β-1,3-glucan that serves as a temporary cell wall under stressful conditions, were identified as templates for silicification.^94^ Silica deposition exactly mimics callose development in horsetail and in fern.^94^ In *in planta* studies, callose, and the production thereof, shows strong associations with silicification in recent imaging and genetic work.^103,104^ The teams suggest that hydrogen bonding between silicic acid and callose plays a key role in silicification.^103,104^

Si-accumulating rice and horsetail plants synthesize a mixed-linkage glucan ((1;3,1;4)-β-d-glucan), which has also been studied for its effects on plant silicification.^105^ When the synthesis of this mixed-linkage glucan is downregulated *in planta*, the total amount of silica accumulation remains unchanged but silica distribution throughout the plants is significantly altered, suggesting this polysaccharide significantly modulates silicification distribution.^105^

Siliplant1 is the first protein suggested to strongly promote silicification in plants. It was isolated from the apoplast surrounding the aforementioned silica cells by Kumar et al. in 2020.^102^ Kumar et al. suggest the zwitterionic nature of this molecule may have a role in promoting silicification.^69^ It is unknown whether this peptide is post-translationally phosphorylated similarly to silaffins.^106^ Overall, silicification in plants is an unexplored area for investigation.

## 4 Studies of Organic Molecule-Directed *in Vitro* Silicification

Many studies have probed the influence of natural and synthetic molecules on silicification. Some investigators intended to mimic or understand natural biosilicification, while others were motivated to develop new biocomposite materials. Collectively, the literature shows that a mechanistic picture is not yet established for how macromolecule composition and structure regulate mineralization, owing to at least three ongoing limitations. First, few studies were designed with quantitative control of reaction conditions or the characterization of the solutions, reacting materials, or final products. While contributing descriptive insights, these approaches cannot establish a quantitative framework for comprehensive physical models. Second, and related to the first, is the fact that few studies monitor (or report) the chemical driving force for polycondensation. This information is critical to building a picture of the kinetic or thermodynamic properties and providing the full package of data necessary to complement modeling studies. Finally, many previous studies use disparate (or uncharacterized) macromolecule compositions, which further limit direct comparisons. As a result, a number of *in vitro* studies yield seemingly opposite conclusions, thus leading to greater confusion in the literature. In this discussion, we highlight studies that provide insights into macromolecular controls on silicification while also demonstrating that a consistent picture has not emerged.

### 4.1 Protein and Peptide-Directed Silicification

Proteins and peptides are the most extensively studied classes of macromolecules for promoting silicification due to an array of analytical techniques available to manipulate and analyze structure and function.^107,108^ Simple methods are used to extract a wide variety of soluble proteins and peptides from biosilica (e.g. Figure 7B,D).^19^ Their associations with sites of biosilicification serve as a guide for the bioactivity in modulating silicification. The composition and structure of the extracted proteins are analyzed through a variety of amino acid characterizations,^19^ genomic, transcriptomic, and proteomic data analysis, and transcription manipulation experiments.^19,34^ The resulting extracted macromolecules are subsequently examined *in vitro* to better understand how nature directs biosilicification.

The following discussion of protein and peptide influences on biosilicification is organized into five major categories: silicatein-based proteins, silaffin-based proteins, plant-based proteins, miscellaneous proteins and peptides, and finally peptidomimetics.

#### 4.1.1 Silicatein-Based Protein-Directed Silicification)

In 1999, Cha et al. extracted a new class of proteins that composed 70% of the spicule filament of the sponge *Tethya aurantia*.^109^ These proteins rapidly precipitated SiO_2_(*am*) from silicon alkoxides, in contrast to the relatively small amount of SiO_2_(*am*) that was precipitated by the control, silk, cellulose, trypsin, BSA, or papain.^109^ Cha et al. named this newly discovered class of proteins “silicateins” due to their apparent silicification catalytic abilities (see Section 3; Table 4).^109^ Silicatein sequences conserve the same arrangement of disulfide bonds (and 3-D structure) that is found in cathepsin proteins.^109^ Cathepsins are well-known proteolytic enzymes with catalytic triads of His, Asn, and Cys.^109^ Silicateins also conserve cathepsin His and Asn residues but replace Cys with Ser (Table 4).^109^

**Table 4:** Summary of Silicification Studies Conducted with Silicatein-Based Proteins and Peptides under Conditions of pH, Time, Temperature, and Solvent

Protein/Polypeptide Substrate	Selected Functional Groups (FGs)	Source of Si Monomer	Conditions	Characterization Methods and Findings	Reference
**Silicatein filaments, Recombinant silicatein**-α from T. aurantia sponge	S–H–N catal. triad	TEOS or C_12_H_20_O_3_Si	pH 6.8	**Molybdenum Blue**:^110^ Controls produce 6.7 nmol–10.2 nmol SiO_2_, compared to 214 nmol SiO_2_ in the presence of 0.06–0.3 mg silicatein-α subunits after 15–60 min. BSA (42.1 nmol Si), papain (22.9 nmol Si), and trypsin (16.2 nmol Si) show similar activity to denatured silicatein (24.5 nmol Si). All with TEOS.	Cha et al. (1999)^109^
**Cellulose, silk, BSA, papain, trypsin**	**FG:**		15 min–12 h	**SEM:** Confirms SiO_2_ precipitation with TEOS. Neither silk nor cellulose exhibit SiO_2_ precipitation, suggesting hydroxy groups alone do not accelerate silicification.	
	–OH		20 °C	**NMR:** Silicatein with TEOS shows Q_2_, Q_3_, and Q_4_ suggesting incomplete condensation.	
	–C(=O)NH_2_, −C_3_N_2_H_3_ or −C_3_N_2_H_4_^+^		Tris-HCl buffer		
**Silicatein-α** (and active site mutants and denatured variants)	S–H–N catal. triad	TEOS	Neutral pH	**Molybdenum Blue:**(110) Protein-free control produced 6.7 ± 2.1 nmol SiO_2_ while silicatein-α produced 140.0 ± 6.2 nmol SiO_2_ in 1 h. Thermally denatured silicatein-α retained only 6.3 ± 1.4% activity. Ser-26 replaced by Ala retained 10.9 ± 2.0% activity and His-165 replaced by Ala 8.0 ± 1.9% catalytic activity. Native Silicatein-α relies on Ser-26 and His-165.	Zhou et al. (1999)^111^
	**FG:**		1 h		
	–OH		20 °C		
	–C(=O)NH_2_, −C_3_N_2_H_3_ or −C_3_N_2_H_4_^+^		Tris-HCl buffer		
**Silicatein A1** (and derivatives)	S–H–N or C–H–N catalytic triad	TGS	pH 5.5 or 6.8	**SEM and XRF of native silicatein A1:** Silica particles formed	Povarova et al. (2018)^112^
**Cathepsin L** (LoCath) from *L. oparinae* (and derivatives)	**FG:**		25 mM Tris-HCl or PBS + 150 mM NaCl	**Molybdenum Blue Method:**(110) All activities of derivatives result in similar or higher activity compared to wild type, suggesting that general protein structure affects silicification rather than the catalytic triad.	
**Human** Cathepsin L CTSL (and derivatives)	–OH or −SH				
	–C(=O)NH_2_, −C_3_N_2_H_3_ or −C_3_N_2_H_4_^+^				
**Silicatein-based Peptide mimic**, sequence Ac-LSLHLNL	S–H–N catal. triad, and L	H_4_SiO_4_ (hydrolyzed TMOS)	18 mL H_2_O and 2 mL of 10× PBS	**SFG:** Shows β-turns and β-strands remain undisturbed after silicification. Structural stability possibly because of the accessible catalytic triad.	Strunge et al. (2022)^113^
	**FG:**			**AFM and XPS:** Thin 4.5 nm structures made of protein and silica.	
	–OH				
	–C(=O)NH_2_, −C_3_N_2_H_3_ or −C_3_N_2_H_4_^+^				
**Recombinant Silicatein A1** (LoSilA1) from marine sponge, *L. oparinae*	S–H–N catal. triad	THEOS (0.1 to 1.5 wt %)	pH 6.8	**SEM and EDX:** 1:2 ratio of BSA:THEOS produced triangular and rhombic dodecahedron crystals. 1:2 ratio of Silicatein:THEOS produced 200–300 nm hexa-tetrahedral crystals coated with amorphous silica.	Shkryl et al. (2016)^114^
**BSA control**	**FG:**		24 h		
	–OH		25 °C		
	–C(=O)NH_2_, −C_3_N_2_H_3_ or −C_3_N_2_H_4_^+^		tris-HCl buffer + 100 mM NaCl		
**Recombinant Silicatein-α** (cross-linked with glutardialdehyde and immobilized on Au surface)	S–H–N catal. triad	H_4_SiO_4_ (hydrolyzed TMOS)	Neutral pH	**AFM:** Uniform coatings of SiO_2_ (20–100 nm thick, 1.2–5.2 nm rough) observed after 120 min. Film roughness and thickness increase with higher silicatein concentrations. No homogeneous films produced at lower silicatein concentrations.	Rai and Perry (2010)^115^
	**FG:**		Ambient T	**SEM:** No silica forms in silicatein-free controls. With silicatein, initial silica layers serve as a template for thicker, more continuous films after 2 h.	
	–OH		30–120 min		
	–C(=O)NH_2_, −C_3_N_2_H_3_ or −C_3_N_2_H_4_^+^				

**Figure 14:**
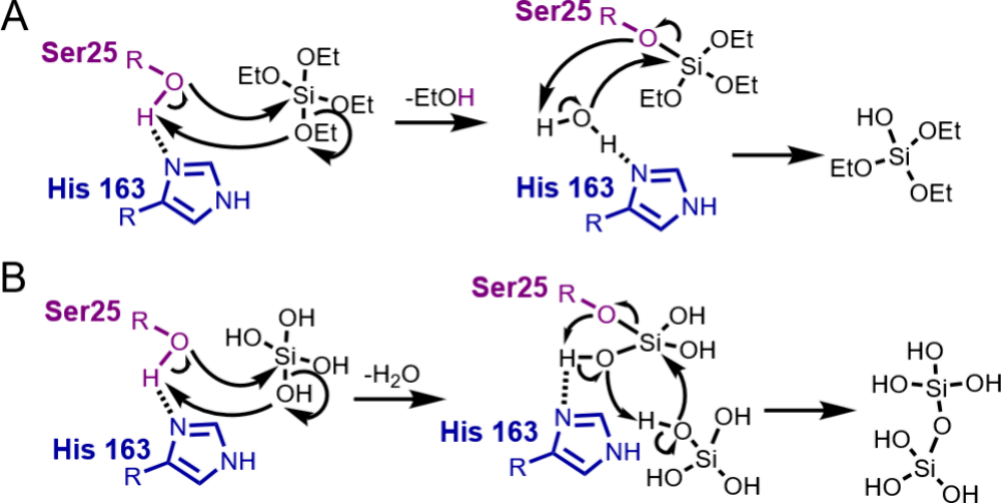
Depiction of hypothesis for silicatein-catalyzed silicification. (A) Hydrolysis of TEOS by silicatein. Ser-25 and His-163 bind via hydrogen bonding, and then, the Ser-25 oxygen attacks electrophilic Si of TEOS. The reaction concertedly extracts serine’s proton and leaves serine bound to TEOS and a water molecule hydrogen bound to histidine. The process is repeated to hydrolyze ethanol from TEOS. (B) Ser-25 and His-163 hydrogen bond before serine’s oxygen attacks monosilicic acid’s electrophilic Si, which concertedly extracts serine’s proton. The process is repeated with a second monosilicic acid molecule, forming a dimer (after Povarova et al., 2018).^112^

The mechanism for silicification catalysis by silicateins was proposed as in Figure 14.^109,112^ Silicatein catalysis of silicification is hypothesized to be based on a series of hydrogen bonding and acid–base reactions via His and Ser residues (Figure 14).^109^ First, Ser-25 and His-163 residues hydrogen bond, before the serine oxygen attacks the electrophilic Si atom and the Si-bound oxygen attacks Ser-25’s hydroxy group proton. This binds the tetraethyl orthosilicic acid or the monosilicic acid molecule to Ser-25 before similar reactions occur, producing silica.^109^ It is notable that poorly water-soluble silicon alkoxides (TEOS), rather than first being hydrolyzed into monosilicic acid, were used to test the silicification-promoting activity of silicateins (Figure 14).^109^

Subsequent studies investigated silicateins or derivatives thereof to catalyze the formation of amorphous silica. In 2018, Povarova et al. argued that silicification-promoting activity is not due to a catalytic triad active site.^112^ Rather, Povarova et al. compared interactions of silicatein with silicic acid to “surface-templating,” by which a wide array of accessible proteinaceous macromolecular functional groups induces silicification.^112^ By explaining this effect as “surface-templating,” Povarova et al. indicated that silicatein simply provides a substrate for heterogeneous silica nucleation, which has a much lower Gibbs free energy barrier compared to homogeneous nucleation (see also Sumerel et al.^116^). This process for directing silicification sharply contrasts with the catalytic triad pathway proposed previously.^112^ Povarova et al.’s claim was supported by evidence that catalytic triad-lacking mutants of silicatein repeatedly produced higher concentrations of silica using an unhydrolyzed, water-soluble silicic acid precursor (Table 4).^112^ While many studies track the rate of silicification, few studies report quantitative kinetic measurements regarding the effects of silicatein or probe the Si–silicatein interaction. Thus, the physical basis for silicatein activity remains open for discussion and requires thermodynamic and kinetic experimental measurements and complementary modeling.

#### 4.1.2 Silaffin-Based Protein-Directed Silicification

In the same year that silicatein was identified, Kröger et al. discovered the silaffin proteins in diatoms (Section 3.1.1).^67,109^ By experimenting with silaffins isolated from frustules of *Cylindrotheca fusiformis* via *in vitro* methods, they found the amount of silica precipitation was proportional to the amount of silaffin protein added, and condensation occurred much faster than the protein-free controls (Table 5).^67^ This effect was especially pronounced at pH 5.^67^ It is notable that the solvent used in this experiment was a phosphate buffer solution.^67^

**Table 5:** Summary of Silicification Studies Conducted with Silaffin-Based Proteins and Peptides under Conditions of pH, Time, Temperature, and Solvent

Protein/Polypeptide Substrate	Selected Functional Groups (FG)	Source of Si Monomer	Conditions	Characterization Methods and Findings	Reference
**Silaffin-1A** (4 kDa)	**Silaffin 1A:** S, K, G, Y	H_4_SiO_4_ (hydrolyzed TMOS)	pH 3–7	**β-Silicomolybdate:** Silaffin-1A promoted silicification at pH > 3, peaking around pH 5. pR5 does not promote silicification until pH > 6. SiO_2_ does not precipitate for many hours in the protein-free control while silaffins condense H_4_SiO_4_ into metastable silicic acid in seconds. SiO_2_ precipitation is proportional to added silaffin: 0–25 μg of protein produces 0–500 nmol of silica.	Kröger et al. (1999)^117^
**Silaffin-1B** (8 kDa)	**FG:**		5 min	**SEM:** Silaffin-1A produces spherical particles with diameters 500–700 nm. Silaffin mixture produces rough silica particles, diameters <50 nm.	
**Silaffin-2** (17 kDa) (all lacking phosphate groups)	–OH		Ambient T		
**pR5** (synthetic peptide, repeat unit of silaffin 1A lacking post-translational modifications)	–NH_2_ or −NH_3_^+^		Sodium phosphate-citrate buffer		
**Phosphorylated Silaffin-1A** (natSil-1A) (8 mol P for every 1 mol silaffin at the serine residues)	S, K, G, Y	H_4_SiO_4_ (hydrolyzed TMOS)	pH 5.5	**SEM:** Phosphorylated silaffin-1A in 50 mM sodium acetate produces a comparable amount of SiO_2_ 400–700 nm nanospheres to unphosphorylated silaffin-1A in 30 mM PBS. Unphosphorylated silaffin-1A in sodium acetate shows no silicification.	Kröger et al. (2002)^68^
	**FG:**		0–10 min	**^31^P NMR:** Linewidth broadening suggests silaffins electrostatically aggregate.	
	–OH		50 mM sodium acetate solution, 30 mM PBS, or 3 mM PBS		
	–NH_2_ or −NH_3_^+^				
	–HPO_4_^–^				
**Peptide 1:** lacks two N-terminal serines	S, K, G, Y, C	H_4_SiO_4_ (hydrolyzed TMOS)	pH 7	**SEM:**Peptides 1, 4, and 5: smooth SiO_2_ particles 250–500 nm in diameter. Peptides 2 and 3: particles with rough surfaces.	Kamalov et al. (2018)^118^
**Peptide 2:** native-like R5	**FG:**		Ambient T	**LCMS:** Dimerization of Peptide 5 during silicification increased from 0 (*t* = 0) to the maximum at 8 h, likely due to disulfide bridging.	
**Peptide 3:** N-terminal serines phosphorylated	–OH		30 min		
**Peptide 4:** Cy5 conjugated	–NH_2_ or −NH_3_^+^				
**Peptide 5:** N-terminal cysteine attached	–HPO_4_^–^				
	–SH				
**R5** (lacking post-translational modifications, incorporated into cross-linked pentaacrylate)	S, K, G, Y	H_4_SiO_4_ (hydrolyzed TMOS)	pH 8	**SEM and EDS:** Silica spheres (diameter: 452 ± 81 nm) formed a regular 2-D array with the periodicity of the hologram enriched with R5 peptides. Silica sphere formation/patterning was not observed in the absence of R5.	Brott et al. (2001)^119^
	**FG:**		10 min		
	–OH		Sodium phosphate-citrate buffer or water		
	–NH_2_ or −NH_3_^+^				
**P5S3** (designed silaffin-like peptide)	K, R, S	H_4_SiO_4_ (hydrolyzed TMOS)	pH 5.4, 7.0, 8.5	**β-Silicomolybdate Method:** At pH 7, after 8 h, increasing P5S3 concentration from 20 to 165 ppm stabilizes ∼178 ppm more silicic acid.	Spinthaki et al. (2017)^120^
**PAA** (MW = 2,000 and 450,000 Da)	**FG:**		0–72 h	Combining PAA and P5S3 strongly reduced molybdate-reactive silica vs P5S3 alone. P5S3 and polyamines aggregate silica at low concentrations and accelerate silica dissolution at higher concentrations.	
**KH**_**2**_**PO**_**4**_**, Pentaethylenehexamine, Tetrapropylenepentaamine**	–NH_2_ or −NH_3_^+^		Sodium acetate, bis-tris-HCl, and tris-HCl buffer solution	**EDS/FTIR:** Only P5S3 is present in SiO_2_.	
	–OH			**SEM:** Control shows nondescript morphology. 60 ppm P5S3 causes spherical particles, and 100 ppm P5S3 causes aggregates.	
	–C(=O)NH_2_			**Conclusions:** P5S3 likely harvests autocondensed silica and leads to precipitation. Stabilization is likely due to amide groups.	
	H_2_PO_4_^–^				
**Silacidin** (silacidin A is 2920.2 Da ∼60% phosphorylated) from *Thalassiosira pseudonana*	S, E, D, G, S	H_4_SiO_4_ (hydrolyzed TMOS)	pH 5.5	**Molybdenum Blue:** In polyamine+silacidin solutions, silicification occurs at a concentration dependent rate, ∼2–3× greater than phosphate ions alone.	Wenzl et al. (2008)^121^
	**FG:**		12 min	**SEM:** With increasing silacidin concentration, larger silica spheres formed. All SiO_2_ had uniform shape and size.	
	–OH		25 mM sodium acetate		
	–COO^–^				
	–NH_2_ or −NH_3_^+^				
	–HPO_4_^–^				

Silaffins have distinct characteristics including post-translationally bound cationic polyamines and a common repeat unit, which Kröger et al. synthesized and isolated, calling it “pR5”.^67^ The pR5 peptide facilitated a much slower production of silica in contrast to the entire silaffin protein and primarily promoted silicification at pH > 6 (Table 5).^67^ Therefore, minimal activity was largely attributed to the pR5 sequence.^67^

Other teams since confirm a similar ability of the silaffin R5 repeat unit to promote silicic acid condensation at higher pH values. The R5 unit is the same amino acid sequence of pR5, but it includes post-translational modifications that pR5 lacks.^67^ Kamalov et al. suggested the *N*-terminus has a strong impact upon secondary and tertiary structure, which likely affects its silicification-promoting activity (Table 5).^118^ These studies were conducted at pH 7, rather than the suggested physiological pH of 5–6 for silicification in diatoms and sponges, thus potentially limiting their relevance to biosilicification in these organisms.^40,66^

In a later study, Kröger et al. isolated silaffins from *C. fusiformis* using a milder method. Extracting the silaffins with NH_4_F (instead of HF) revealed post-translationally phosphorylated silaffins.^68^ Their findings suggest phosphate groups are vital to promoting the silicification activity of silaffins (Table 5).^68^ Recall that the previously HF-extracted silaffins also were able to promote condensation, albeit to a lesser degree, likely due to complexation with free phosphate groups in PBS solution.^67,68^ The investigators concluded the activity of silaffins can be attributed to their zwitterionic character.^68^

In 2017, Spinthaki et al. synthesized a silaffin-like protein, P5S3, which inhibited silica condensation at conditions 4–8× supersaturation with respect to amorphous silica and enhanced silicification at 15–30× saturation (∼30 mM H_4_SiO_4_) (Table 5).^120^ The team proposed the protein’s inhibitory ability is due to the backbone amide groups nonelectrostatically directed silicification while the grafted polyamines electrostatically controlled silicifcation.^120^ These findings suggest silaffin activity is dependent on the supersaturation of silicic acid and there is no particular active site. Rather, it is likely charge–charge interactions or inductive effect interactions are at play.^120^

In studying silacidins, another significant family of silica-active proteins, Wenzl et al. determined particular functional groups have a strong, yet not fully elucidated effect on silicification, consistent with previous studies.^121^ The team reported cationic polyamines alone do not promote silicification, but the addition of an anionic silacidin phosphoprotein accelerates silicification (Table 5).^121^ The effects of polyions on silica formation will continue to be a common theme in the discussion of silicification.

#### 4.1.3 Plant-Based Protein-Directed Silicification

**Figure 15:**
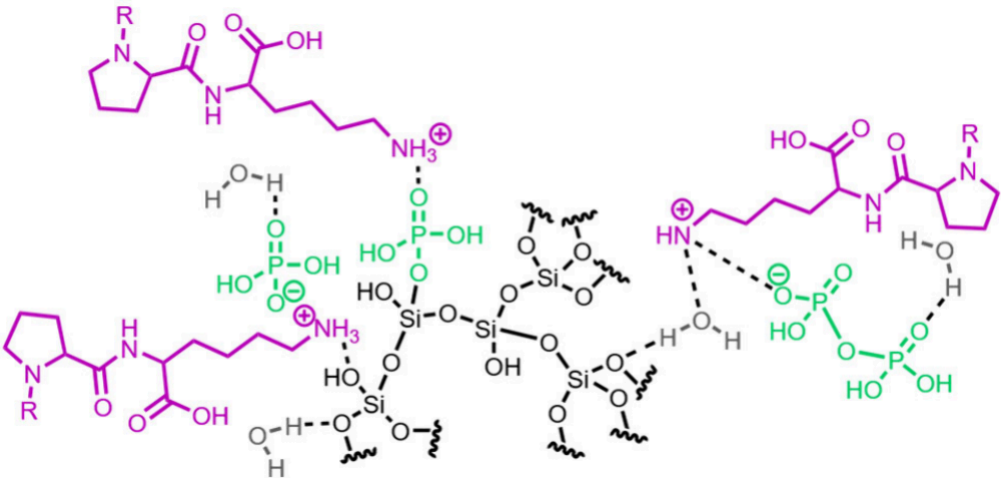
Proposed associations of siliplant proteins with silicic acid species. Electrostatic binding between lysine (pink) and phosphates (green) is likely, as are hydrogen bonds formed between silicic acids or silica with phosphate groups (after Adiram-Filiba et al., 2020).^106^

Plants also contain proteins that show evidence of modulating silicification. In 2020, Kumar et al. isolated siliplant proteins from sorghum plants (see Section 3.3).^102^ NMR analyses showed these proteins are intimately associated with silica.^102^ They also found phosphates promote silicification in the presence of siliplant proteins, and NMR studies further indicate that Si–O–P bonds could form during silicification (Table 6).^106^ The anionic phosphates and cationic lysine amines likely are bound electrostatically, and in some cases, the phosphates are additionally stabilized by lysine residues via hydrogen bonding (Figure 15).^122^

**Table 6:** Summary of Silicification Studies Conducted with Plant-Based Proteins and Peptides under Conditions of pH, Time, Temperature, and Solvent

Protein/Polypeptide Substrate	Selected Functional Groups (FGs)	Source of Si Monomer	Conditions	Characterization Methods and Findings	Reference
Proteases:	Polyamines:	Na_2_SiO_3_	pH 7	**SEM:** Smooth spherical particles 125–325 nm in diameter (no proteases). Bromelain and papain produce particles similar to the control, and trypsin produced smaller and more irregular sized particles.	Baker et al. (2014)^123^
**Bromelain, Papain, Trypsin**	–NH_3_^+^ or		2 min		
Amines:	–NH_2_		25 °C		
**DETA, TETA, TEPA, and PEHA**	–NH_2_^+^–		100 mM phosphate buffer		
3 Segments of Proline-rich Protein (PRP1):	Y, P, K, R	H_4_SiO_4_ (hydrolyzed TMOS)	pH 5–8	**β-Silicomolybdate:** Silicification peaks pH ∼ 6.25 for Pep1 and Pep2, and each has a dose-dependent linear relationship with silicification, unlike Pep3, likely due to charge density characteristics. These peptides seem to influence silicification identically. Silicification peaks with Pep3 pH ≈ 8. Silicification slightly increases with BSA concentration, likely due to hydrogen bonding and BSA’s high MW.	Kauss et al. (2003)^122^
**Pep1** (9 cationic residues)	**FG:**		5 min		
**Pep2** (9 cationic residues)	–OH		25 °C		
**Pep3** (6 cationic residues)	–NH_3_^+^ or −NH_2_ or =NH_2_^+^		Sodium phosphate/citrate buffer		
**BSA Control**					
Siliplant1–Peptide 1	P, K, E, H	H_4_SiO_4_ (hydrolyzed TMOS)	pH 7	**Raman spectroscopy:** Vibrations at 922, 724, and 598 cm^–1^ suggest protein −COO^–^ interacts with silica −OH groups.	Kumar et al. (2020)^102^
Siliplant1–Peptide 3	**FG:**		5 min	^**1**^**H,**^**13**^**C, and**^**29**^**Si NMR:** Narrow peaks (fwhm ≈ 282 Hz), and peaks shifting to a higher field ∼2 ppm), suggest the peptides complex with SiO_2_. Mostly Q_4_ species were seen in the polymerized silica.	
	–NH_3_^+^ or −NH_2_		0.1 M potassium phosphate buffer solution	**SEM:** Peptide 1 precipitates 500 nm SiO_2_ spheres at 90.9 mM H_4_SiO_4_, while lysine-free Peptide 3 did not show silica precipitation.	
	–C_3_N_2_H_3_ or −C_3_N_2_H_4_^+^				
	–COOH or −COO–				
**Siliplant 1 peptide** (SLP1, pI = 10.2, possibly β-hairpin conformation with external lysines)	P, K, E, H, D	H_4_SiO_4_ (hydrolyzed TMOS)	pH 7.1	**Observations:** In buffer, SiO_2_ gel forms after 24 h. With peptides, turbidity observed within 1–4 min.	Adiram-Filiba et al. (2020)^106^
**Silaffin peptide** (PL12, pI = 10.6, random coil)	**FG:**		50 or 100 mM of PBS or HEPES buffer	**SEM:**PL12+PBS and SLP1+PBS – 500 nm spherical particles form within the first 5 min.	
	–NH_3_^+^ or −NH_2_			PBS – In 30 min 135 nm spherical particles form.	
	–C_3_N_2_H_3_ or −C_3_N_2_H_4_^+^			PL12-HEPES – First, a SiO_2_ film forms, followed by grainy 60–80 nm particles, which conglomerate after 30 min.	
	–COOH or −COO–			HEPES – Formed SiO_2_ film, then grainy particles, that then fuse into 100 nm spherical particles.	
				**1-D and cross-polarization**^**31**^**P NMR:** The 2.1 ppm peak on spectra for PBS, PL12-PBS, and SLP1-PBS SiO_2_ is attributed to physiosorbed phosphate ions. A peak at −6.3 ppm is seen in the SiO_2_ solutions with PBS and protein, suggesting increased water-phosphate binding. −5 to −45 ppm peaks are associated with P–O–Si bonds.	
				**29Si{**^**31**^**P} REDOR:**SLP1-PBS-SiO_2_ – 11% decay of Q_3_ signal observed after 12.8 ms of REDOR recoupling, due to dipole–dipole coupling of surface Si with ^31^P. The short dephasing indicates 1 bond length separation.	
				**SEDRA and 2-D DARR**^**31**^**P NMR:**PL12-SiO_2_ and SLP1-SiO_2_:^31^P–^31^P distances ≈2.85 Å, a distance similar to phosphates in NaP_2_O_7_.	
				**Comparing 2-D**^**1**^**H-**^**31**^**P,**^**1**^**H-**^**13**^**Cand**^**1**^**H-**^**29**^**Si spectra:** Protein Lys residues interact with SiO_2_ surface and phosphates. Lys displaces water from SiO_2_ surface.	

Adiram-Filiba et al. subsequently suggested that siliplant proteins may be phosphorylated similarly to many silica-directing proteins.^106^ They also found that lysine residues could displace a portion of the water molecules away from silicic acid, potentially suggesting a form of a hydrophobic effect (Table 6).^106^

Kauss et al. investigated three peptides from the proline and lysine-rich protein (PRP1), extracted from cucumbers.^122^ This protein was thought to play a role in silicification as it was consistently upregulated in their study to resist plant pathogens.^122^ The investigation demonstrates that peptides with higher charge density promoted the most silicification, regardless of primary amino acid sequence (Table 6).^122^ Thus, Kauss et al. concluded this protein is likely involved in the fortification of cucumber plant walls with silica.^122^ The next section, discussing other proteins and peptides not typically associated with silicification, continues to examine evidence for macromolecular charge as a factor in silicification.

#### 4.1.4 Miscellaneous Protein- and Peptide-Directed Silicification

Various proteins have served as model systems for studying the influence of specific characteristics on silicification. For example, Gautier et al. tested the effects of positive charges on silicification using the highly cationic protein gelatin.^124^ Gelatin promotes silicification due to its high charge density, which is consistent with the previous discussions of PRP peptides, silaffins, silacidins, and siliplant proteins (Tables 4–7).^124^

**Table 7:** Summary of Silicification Studies Conducted with Miscellaneous Proteins and Polypeptides under Conditions of pH, Time, Temperature, and Solvent

Protein/Polypeptide Substrate	Selected Functional Groups (FGs)	Source of Si Monomer	Conditions	Characterization Methods and Findings	Reference
**native sericin protein** (extracted from *Bombyx mori*)	–OH	C_18_H_12_K_2_O_6_Si	pH 6.8	**Colorimetric:**Sericin proteins show no statistically significant catalytic or stabilization effects on the formation of trimers. Diols: For a Si:OH ratio of 1:4 and 1:10, trimerization rate constants increase with increasing diol chain length but oligomerization kinetic constants are unchanged. Potentially, diol micellization aids formation of silica, with a critical micelle concentration at the 1:4 ratio. Ethanediol and propanediol may stabilize silicic acid via H-bonding.	Tilburey et al. (2007)^125^
**recombinant sericin precursor peptide**			0–100 h	**Photon Correlation Spectroscopy (PCS):** Protein aggregates dissociate as silica is formed. Silicification occurs at a rate similar to the control, suggesting only weak intermolecular interactions between silicic acid and proteins. Diols: In KCl and catechol solutions, micellization of diols is seen with chain lengths of 6 and 7 carbons but not for shorter chains.	
**alkanediols**			Deionized distilled water	**SEM:** At higher ratios of protein in silica (Si:OH is 1:1), morphological differences compared to the blank are observed. Diols: Particles 200–400 nm in diameter are formed with chains of greater than 3 carbons, and no particles formed with shorter chained diols.	
				**Conclusions:** Hydrophobicity/phase separation and H-bonding have the biggest effects on silicification.	
**Gelatin** (polycationic protein, MW ≈ 40 kDa)	K, R, E, D	Na_2_SiO_3_ (27% SiO_2_, 10% NaOH)	pH ≈ 5	**Observation:** Instant precipitation of white solid when Na_2_SiO_3_ is added to gelatin and gelatin+alginate solution at 37 °C. This does not occur in the presence of pure alginate. Control forms SiO_2_ gel overnight.	Gautier et al. (2008)^124^
**Alginate**	**FG:**		1 h at 37 °C then 20 °C for 1 day	**TGA:** All gelatin was associated with silica while a small fraction of alginate was associated with silica.	
	–NH_3_^+^ or −NH_2_		DI water	**TEM:** SiO_2_ pores 100–200 nm in size in all except for pure alginate-silica, which showed 5 nm nanoparticles.	
	=NH_2_^+^			**Conclusion:** Gelatin activates silica formation, but both polymers interact to control the silica morphology	
	–COOH or −COO^–^				
**Silk, 6mer and 15mer silk peptides**	**Silk:** S, G, L, R, Q	H_4_SiO_4_ (Hydrolyzed TEOS)	pH 7	**Molybdenum Blue:** The condensation from trimers to oligomers was slower for the Silk-Pep1 chimera compared to Pep1. Overall, the silk-based biomolecules decreased the rate of oligomerization and promoted oligomer dissolution. Increasing molecular weights of the molecules increased these effects, possibly due to monosilicic acid complexation and stabilization via the peptides.	Canabady-Rochelle et al. (2012)^43^
**Silica binding peptide** (Pep1)	**FG:**		Time: 0–24 h	**SEM/EDX:** No biomolecule control and silk: SiO_2_ formed as granular spherical particles ∼50 nm in diameter.	
**Silk or silk peptide-silica binding peptide chimeras** (bound via EDC coupling)	–OH		1 M citric acid and 73 μL of 1 M bis-tris propane, water, and ethanol	Pep1: SiO_2_ particles ∼100 nm diameter	
	=NH_2_^+^			Chimeras: 6mer-Pep1 caused the most dramatic difference in SiO_2_ with aggregates ∼690 nm in diameter and more monodispersity.	
	–C(=O)NH_2_				
	**Pep1:** S, K, R, H, D				
	**FG:**				
	–OH				
	–NH_3_^+^ or −NH_2_				
	–C_3_N_2_H_3_ or −C_3_N_2_H_4_^+^				
	=NH_2_^+^				
**MAX8** (β-sheet former)	K, T, E, V, P	**TMOS** (used to silicify with MAX8)	pH 9	**TEM and SEM:** SiO_2_ formed only around the protein fibrils. This templating effect is suggested to be due to the external lysine groups accessible to the SiO_2_ precursor.	Altunbas et al. (2010)^126^
**MAX1**	**FG:**	**TEOS** (used to silicify with MAX1)	1 h	**Modulus:** MAX8 exhibits shear thinning capabilities.	
	–OH		Ambient T		
	–NH_3_^+^ or −NH_2_		DI water with HEPES or with borate buffer		
	–COOH or −COO^–^				

**Figure 16:**
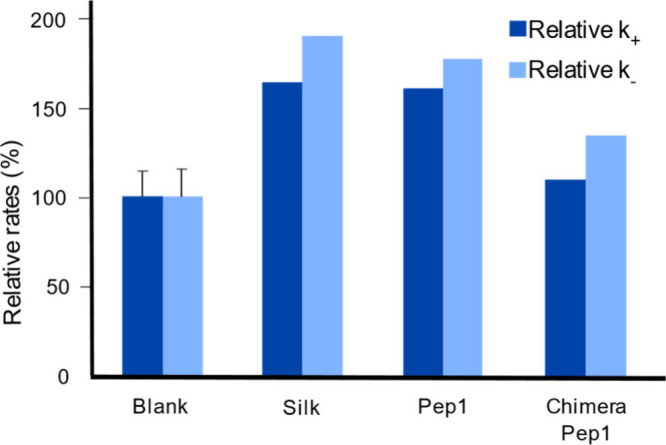
Relative first order rate constants for the transition from trisilicic acid to oligomers in the presence of three proteins. The dissolution rate constant (*k*_–_) is higher than the forward first order rate constant (*k*_+_), slowing the net rate of silicification (30 mM Si) in the presence of the macromolecules tested (after Canabady-Rochelle et al., 2012).^43^

In contrast, Canabady-Rochelle et al. tested minimally charged, R5 conjugated silk-based biomolecules that lacked amine groups on silicification.^43^ This team quantified monosilicic acid concentrations over time using the molybdenum blue method. The concentration data were evaluated assuming a reversible first order kinetics described by ln(*A* – *A*_f_), where *A* = H_4_SiO_4_° concentration at time *t* and *A*_f_ = monosilicic acid concentration at the final experimental time. The equilibrium between trisilicic acids and oligomeric silicic acids was assessed via a first order kinetic rate expression (see more information regarding the methods used in Harrison and Loton^127^). These peptides decrease the rate of silicic acid oligomerization, mathematically represented by the first order rate constant, *k*_+_, and increase the rate of silicic acid oligomer dissolution, *k*_–_, at pH 7 (Figure 16, Table 7).^43^ Silicic acid trimer dissolution is correlated with higher protein molecular weights; therefore, the team suggests that larger biomolecules stabilize charged monosilicic acid (Table 7).^43^ Furthermore, the team proposed that the silica that does form in conjunction with chimera proteins is the result of a scaffolding or aggregation effect, like that of the R5 peptide, alone, rather than a specific catalytic event.^43^ Analysis of the transition between dimers and trimers in the presence of the protein additives provide this insightful information through the extraction of relative first-order rate constants (Figure 16, Table 7).^43^

Using a highly hydroxylated protein along with a series of diols, the impact of −OH group concentration upon silicification was examined.^125^ The macromolecules’ number of hydroxy groups, and thus hydrogen bonding abilities with silicic acid, was a negligible factor in promoting silicification (Table 7).^125^ Instead, the observed minor silicification was thought to be due to the formation of hydrophobic micelles by the longer chain diols.^125^ These results provide insight into our discussion of the effects of hydrogen bonding between organic molecules and silicic acid in controlling silicification.

Higher-order protein structures may also play a role in silicification. This effect was investigated using laboratory-designed β-sheet-forming MAX proteins. The stimuli-responsive MAX proteins remained folded as silica precipitated onto them, suggesting that 3-D effects could affect silicification (Table 7).^126^

Taken together, these studies suggest the primary sequences of proteins do not have active roles in biosilicification. Rather, specific functional groups (often ionized) confer activity in controlling silicification. Phosphoryl and amine groups appear to play the most important roles in promoting silica formation. In contrast, it is possible the role of hydrogen bonding via the hydroxy group is negligible. Hydrophobicity and phase separation may also influence the silicification. The collective evidence reiterates that functional groups, rather than molecular class, are likely a key to understanding macromolecule controls on mineralization.^73^

#### 4.1.5 Peptidomimetic-Directed Silicification

**Figure 17:**
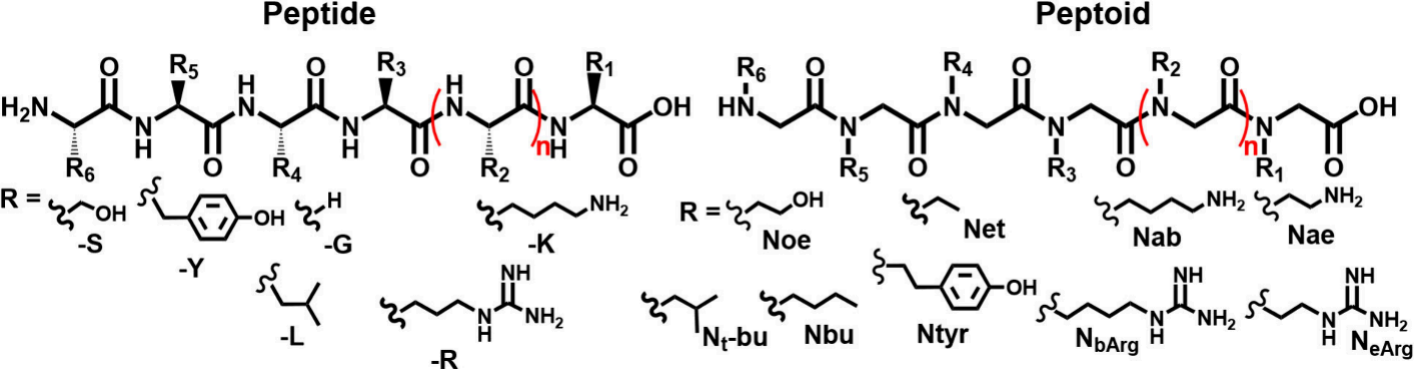
Structural differences between a peptide and a peptoid with examples of side chain similarities.

Peptoids have emerged as useful materials for mimicking peptides with specific structures and establishing the roles of particular functional groups and a large variety of sequences in controlling inorganic crystallization,^128−130^ including silicification.^131,132^ These poly(*N*-substituted glycine) polymers (Figure 17) are similar to peptides but offer several advantages for studies of functional group and motif controls on mineralization. First, the substitution on the backbone amide nitrogen, rather than the α-carbon, precludes hydrogen bonding donation at this site. Second, the backbone of these polymers is achiral, therefore, higher order molecular structure is dependent on the side chains and therefore tunable.^129,133,134^

Developed in the late 1980s, this relatively new class of polymers has grown in popularity for researching protein mimicry, to control the formation of material hierarchical assembly, and to develop biomimetic mineralization approaches.^135^ For example, Chen et al. show calcite crystal growth is accelerated 23× by the presence of an amphiphilic (anionic and hydrophobic side-chained) peptoid at 50 nM levels.^136,137^ The tunability of peptoids enabled this group to resolve the influence of side-chain length, sequence, and chemical functionality on calcite mineralization.^136,137^ Peptoids have also been designed for controlling the formation of titanium,^138^ calcium phosphate,^139^ metallic nanocrystals,^140−142^ and metal oxides.^130−132^

Despite the tremendous efforts to imitate the activity of proteins and peptides for directed silicification, it remains a significant challenge to mimic the high-level control over silica formation in living organisms, such as the formation of biosilica spicules in demosponges thought to be induced by the highly ordered axial filaments assembled from silicateins. Recently, by designing sequence-defined peptoids containing side chains that bind strongly to silica, Yang et al. demonstrated that self-assembly of these peptoids into fiber structures enables mimicking of both biocatalytic and templating functions of silicatein filaments to form silica nanofibers at near-neutral pH and ambient temperature.^131^ This team further showed that the presence of amino groups is significant for the mineralization of silica on self-assembled peptoid nanofibers.^131^ Molecular dynamics simulation further confirmed that having silica-binding of amino side chains is critical for self-assembled peptoid fibers in triggering silica nucleation and growth.^131^ The formation of a silica shell on peptoid fibers improves the mechanical properties of the peptoid hydrogel networks by nearly 1000×. This highlights the potential of using mineralization to enhance hydrogel materials for applications including tissue engineering.^131^ Furthermore, Yang et al. demonstrated that tuning interpeptoid interactions by varying carboxyl and amino side chains significantly influences the assembly kinetics and final morphologies of peptoid assemblies as scaffolds for directing the formation of silica materials including nanospheres, nanofibers, and nanosheets.^131^ By varying the numbers of amino and carboxyl side chains, we can tune the peptoid–silica interactions to influence silica mineralization.^131^ These results suggest the strategy of designing self-assembled peptoid materials with programmable interpeptoid and peptoid–particle interactions is promising for synthesizing various inorganic nanomaterials.

A common strategy to discern the underlying chemical mechanisms of protein-directed biosilicification has been the use of short peptide sequences with chemistry mimicking those found in natural systems such as the silaffin-derived R5 peptide (see Section 4.1.2). While progress has been made using this approach, many limitations have prevented breakthroughs in biomimicry.

**Figure 18:**
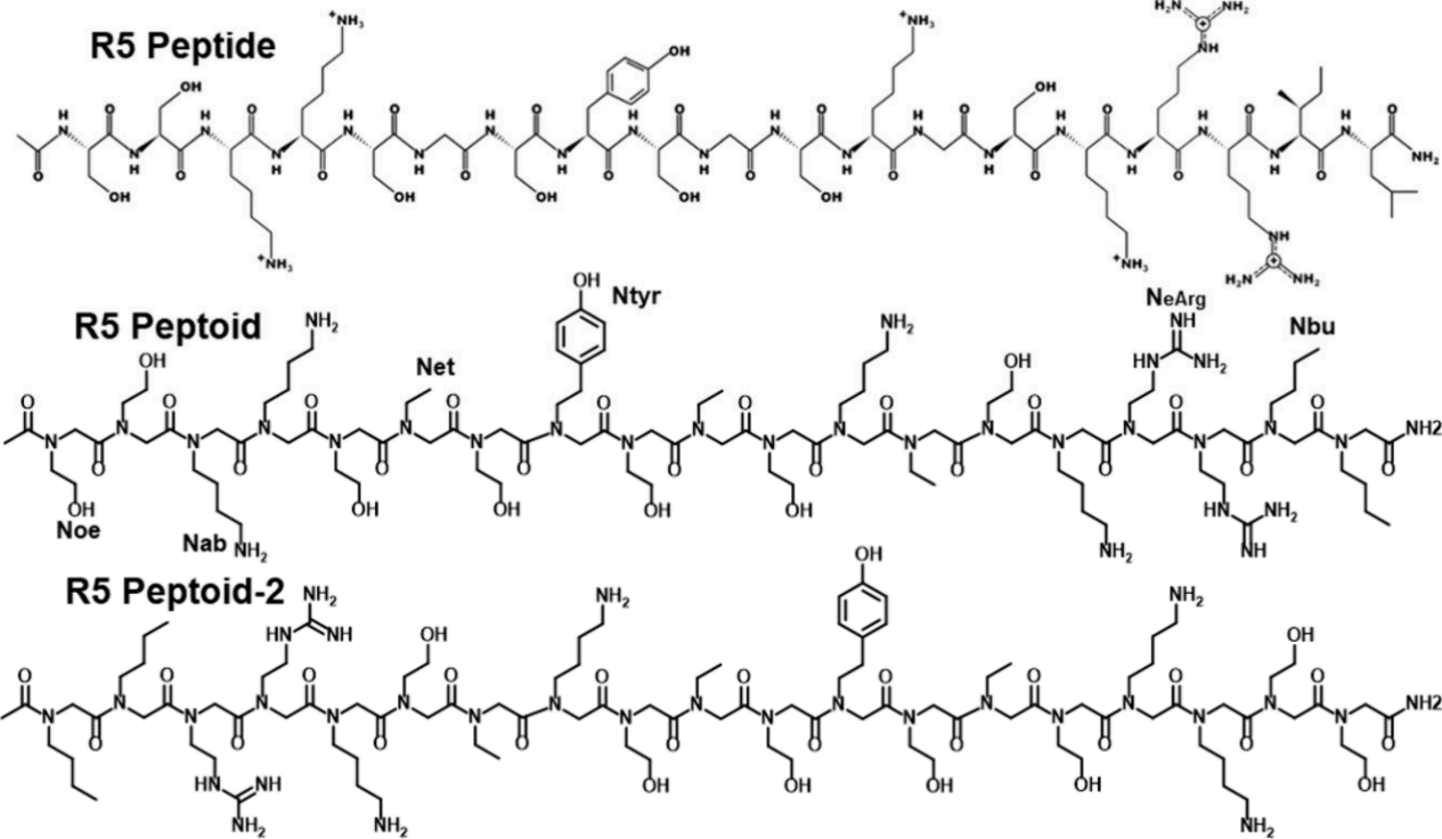
R5 peptide and the synthesized peptidomimetics used by Torkelson et al. to study silicification. R5 Peptoid is also discussed as *toid*R5A, and R5 Peptoid-2 is the reverse analogue of *toid*R5A.^132^

Given that the R5 peptide is well-studied and binds strongly to silica near pH 7, recently, Torkelson et al. used R5 peptide as a resource to computationally design peptoid sequences that can be used for silicification.^132^ Torkelson et al. used the “side chain similarity” approach to design and synthesize R5 peptoid analogs (Figure 17) that mimic R5 peptides for controlling the formation of silica, by using Nab to mimic lysine (K), N_bArg_ to mimic arginine (R), Net to mimic glycine (G), Ntyr to mimic tyrosine (Y), and Nbu to mimic leucine (L) (Figure 18).^132^ This study presents a computationally predicted design of these polymers that are proposed to direct the controlled formation of silica nanomaterials.^132^

**Figure 19:**
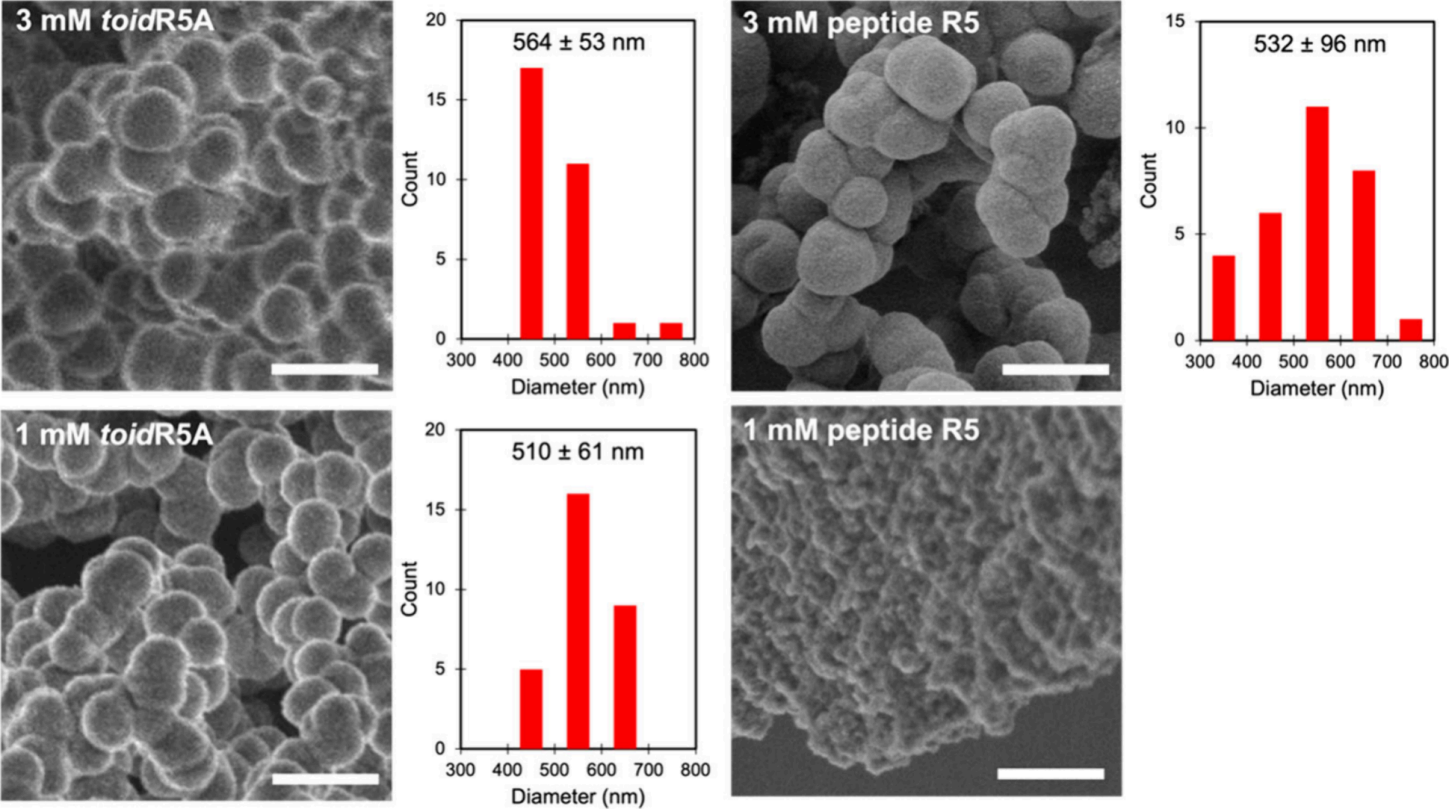
SEM images of silicification products (scale bar = 1 μm) in the presence of 3 mM (top row) or 1 mM (bottom row) of the peptoid *toid*R5A (left column) or the R5 peptide (right column). Each graph represents distributions of particle sizes based on *n* = 30 particles. Reproduced from ref (132). Copyright 2024 American Chemical Society.

Torkelson et al. investigated surface adsorption and the mineralization process through analysis of binding mechanisms and energetics of the R5 system.^132^ The two peptoid analogs validate the computational prediction by showing a higher binding affinity to silica than R5 peptides.^132^ These peptoids were further used to induce the formation of quasi-spherical silica nanoparticles in the 500–550 nm range (Figures 18 and 19).^132^ Through careful analysis of the differences and similarities in the simulations and synthesis outcomes, several key features of biomolecule/silica interactions were proposed as targets for future designs of peptoid sequences to produce spherical silica nanoparticles.^132^

Calkins et al. studied a peptoid-SiO_2_ system to determine the interfacial binding thermodynamics. They show peptoid-silica binding is an endothermic process which depends on peptoid charge and length, as well as the release of water as peptoids adsorb to SiO_2_ surfaces.^143^ Overall, peptoids present untapped potential as simple models to investigate the underlying mechanisms of silicifying proteins.

### 4.2 Polyamine-Directed Silicification

Polyamines are compositionally and structurally diverse molecules commonly associated with sites of biosilicification. They have been studied widely for their potential to template silica as their positive charges are proposed to have roles in modulating silicification (Figure 9, Section 3.1.2, Section 3.2).^2,144^Section 4.1 explores the effects of protein-charged groups and hydrophobicity on silicification. Here, we discuss similar themes with respect to polyamines. It is useful to keep in mind that, while the p*K*_a_ of amine groups in polyamines varies greatly, many natural polyamines are highly protonated at pH values below 7.^71^

**Figure 20:**
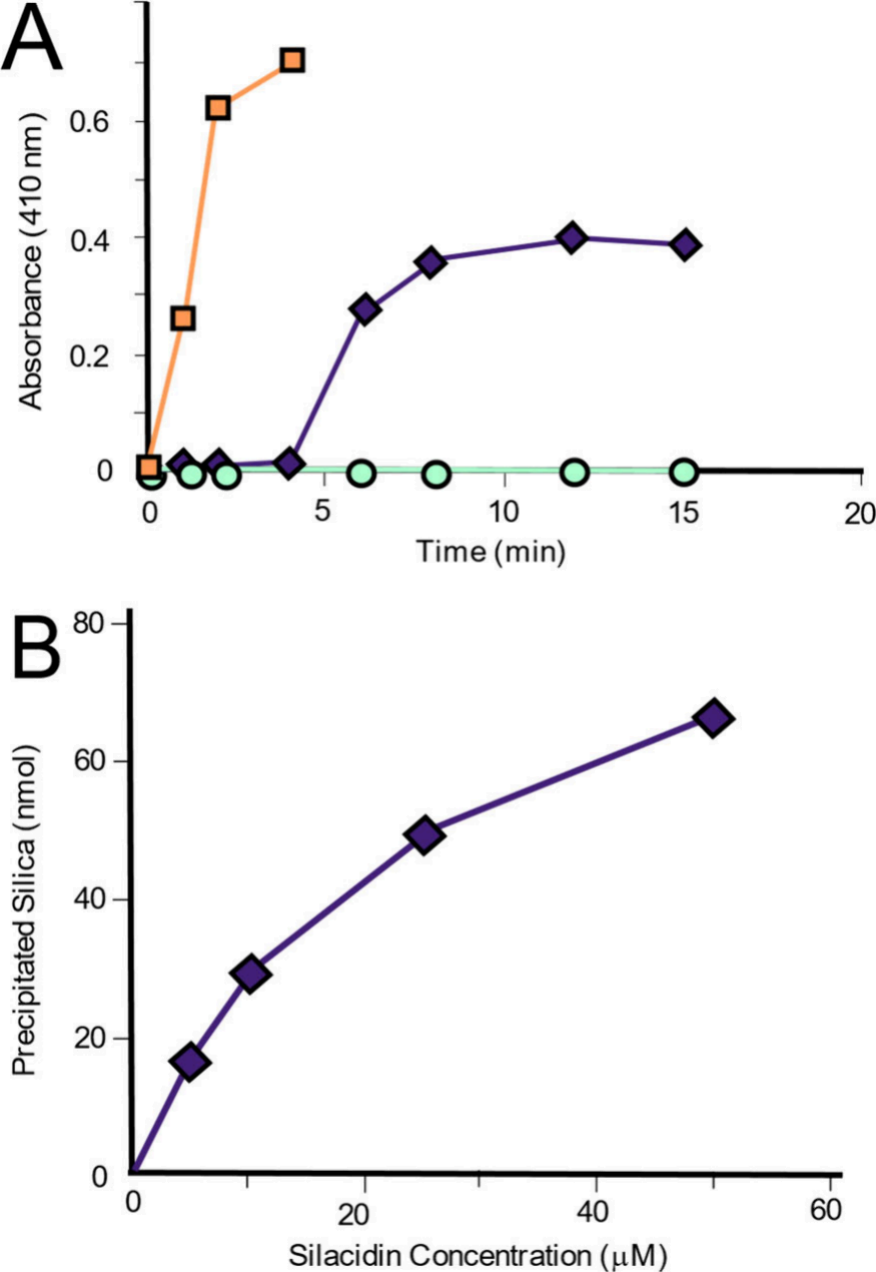
Studies of phosphate-polyamine-directed silicification at pH 5.5. (A) An increase in the absorbance correlates with an increase in precipitated silica. Concentration of formed silica determined via the beta-silicomolybdate method in the presence of polyamines with sodium acetate and silicic acid (green), polyamines added to premixed silicic acid with sodium phosphate (orange), polyamines, sodium phosphate, and silicic acid mixed at *t* = 0 (purple) (after Sumper et al. 2003).^145^ (B) Larger silica spheres, and increasing nmol of total silica precipitates, form in the presence of polyamines with increasing concentrations of phosphates in the form of silacidin (after Wenzl et al. 2008).^121^

At physiological pH, native polyamines require complexation with phosphate groups to promote silicification. Sumper et al. and Wenzl et al. found that natural polyamines do not promote silicification at pH 5.5 (Table 8).^121,145^ Sumper measured this change in silicification rate by collecting the silica formed via centrifugation at specific time points, depolymerizing the precipitated silica using 2 M NaOH, and then quantifying the molybdate-reactive silica that formed using the beta-silicomolybdate method. Therefore, an increase in absorbance (Figure 20A) is related to an increase in precipitated silica. While the team found that natural polyamines mixed with sodium acetate do not promote silicification, polyamines mixed with phosphates promoted silicification. When silicic acid was premixed with phosphate ions for 15 min, then polyamines were added, and rapid silica condensation was observed (e.g., Figure 20A, Table 8).^145^ Wenzl, however, tracked the formation of silica using SEM images and observed that (1) by combining a highly phosphorylated silacidin protein with polyamines, 2–3× more silica formed than phosphate ions alone, (2) increased silica formation correlated with increasing concentrations of silacidin (Figure 20B), and (3) no precipitate was formed in a polyamine–acetate system (Table 8).^121^ They postulated that these effects arose from cooperation between polyamines and phosphate ions to produce an electrostatic effect that controls silicification. Despite the differing methods of investigation, both Sumper and Wenzl found that phosphate-based molecules increased the quantity of silica formed in the presence of polyamines over a specific amount of time.

**Table 8:** Summary of Silicification Studies Conducted with Polyamines under Conditions of pH, Time, Temperature, and Solvent

Polyamine Substrate	Selected Functional Groups	Source of Si Monomer	Conditions	Characterization Methods and Findings	Reference
**Polyamines** (from diatoms, MW ∼ 600–1500 Da)	–NR_2_ or −NR_3_^+^	H_4_SiO_4_ (hydrolyzed TMOS)	pH 5.4–8.3	**SEM:** 1–1.25 kDa polyamines at pH 5, forms 0.8–1 μm SiO_2_ aggregates covered in 100–200 nm silica spheres. 600–700 Da polyamines mostly produce 100–200 nm spheres. Diameter of spheres decreases as pH increases.	Kröger et al. (2000)^146^
			Ambient T		
**Polyamines** (from diatoms, 15–21 *N*-methylpropyleneimine repeating units attached to putrescine)**Phosphates**	–NR_2_ or −NR_3_^+^–	H_4_SiO_4_ (hydrolyzed TMOS)	pH 5.5	**β-Silicomolybdate:** Polyamines alone produce no SiO_2_. Polyamines+phosphates show silicification. Phosphates premixed with silicic acid for 15 min before the addition of polyamines greatly increases silicification.	Sumper et al. (2003)^145^
	–HPO_4_^–^		Sodium phosphate (30 mM) or sodium acetate (30 mM)	**SEM:** With polyamines, pyrophosphate produces larger (1000 nm) nanoparticles than orthophosphate (30 to 700 nm), depending on concentration.	
				^**1**^**H, CPMG NMR:**^1^H NMR shifts and relaxation times suggest polyamine aggregation in the presence and absence of phosphate.	
				**GC:** Monosilicic acid concentration is unchanged by additives. With β-silicomolybdate method results, it is likely that these charged additives only affect oligo- and polysilicic acid condensation.	
**Polyamines** (from *T. pseudonana*) **Phosphates**	–NR_2_ or −NR_3_^+^–	H_4_SiO_4_ (hydrolyzed from TMOS)	pH 5.5	**Molybdenum Blue:** In the presence of polyamines and acetate from the buffer solution, no precipitate forms. In polyamine+silacidin solutions, silicification occurs at a concentration dependent rate, ∼2–3× greater than phosphate ions alone.	Wenzl et al. (2008)^121^
	–HPO_4_^–^		12 min		
			25 mM sodium acetate		
**Ethyleneamines, Propylamines, Spermidine, Spermine, Methylated amines** (norspermidine with amine methylation variations)	–NH_2_ or −NH_3_^+^	H_4_SiO_4_ (hydrolyzed K_2_Si(O_2_C_6_H_4_)_3_)	pH 5.6–7	**Molybdenum Blue:** Spermidine and spermine produced no effect on the 3rd order rate constant of silicification. All propylamines and the longer ethyleneamines significantly increase the rate constant.	Belton et al. (2008)^49^
	–NR_2_ or −NR_3_^+^			**TEM and SEM:** The extent of methylation of the norspermidine series minimally affected SiO_2_ sphere size, which ranged from ∼200 to 300 nm. Hollow SiO_2_ particles (with a central void of 50–100 nm) form with increasing amines in a chain.	
				**Conclusions:** Stability of hydrophobic microemulsions of polyamines translates to increasing ratios of hollow:solid particles.	
**PLL** (MW = 22 100 Da)	–NH_2_ or −NH_3_^+^	K_2_Si(O_2_C_6_H_4_)_3_	pH 6.8	**Molybdenum Blue:** For a H_4_SiO_4_ undersaturated solution, no additives affected H_4_SiO_4_ concentration, regardless of Si:N ratio.	Patwardhan et al. (2011)^147^
**PAH** (MW = 15 000 Da)	–NR_2_ or −NR_3_^+^		0–1000 min	**Dissolution Studies:** Molybdate-reactive silica increases for all reactions from 0 to 1,000 min. Polyelectrolytes show 6× and small molecules show 2–3× faster SiO_2_ dissolution than the control. Small molecules with >5 amines show SiO_2_ precipitate formation. PEHA increases SiO_2_ initial dissolution rate for the first 3–5 min, but SiO_2_ precipitated in the presence of PEHA after 7 min, likely due electrostatic interactions and particle double layer bridging.	
**PEI** (MW = 25 000 Da)			Distilled and DI water	**SEM:** Aggregate sizes ≈131, ≈222, and ≈137 nm form for TEPA, PEHA, and N5, compared to ≈105 nm for the control.	
**DAE, DETA, TETA, PEHA, dipropylenetriamine** (N3), **tetrapropylenepentamine** (N5)					
**Allylamine** (p*K*_a_ = 9.49)	–NH_2_ or −NH_3_^+^	Na_2_SiO_3_	pH 5–7	**Silicomolybdate Method and Turbidity test:** PAH strongly enhances turbidity vs the control at pH 6.8. At pH 5.5, both PAH and control have a 100 min induction period. PAH+phosphate at pH 5.5 dramatically increases turbidity. Phosphate alone retards turbidity. Monomeric amines promote aggregation less efficiently than PAH or PAH+phosphate at pH 6.8. Allylamine Q promotes more silicification than monomeric allylamine, likely due to the hydrophobic effects of additional methyl groups. Phosphate increases silicification with allylamine Q and decreases SiO_2_ formation with allylamine.	Jantschke et al. (2014)^40^
**Fully methylated allyltrimethylammonium bromide (allylamineQ)**	–NR_2_ or −NR_3_^+^		0–800 min	TMEDA, the most methylated molecule, is associated with the most turbidity.	
**PAH** (p*K*_a_ = 9.7)	–HPO_4_^–^ or −PO_4_^2–^			**Conclusions:** Electrostatic interactions and hydrophobic effects have the biggest effects on silicification.	
**Methylated diamines** (EN, MEEN, ENQ, TMEDA)					
**Phosphate**					
**PEHA**	–NH_2_ or −NH_3_^+^	Na_2_SiO_3_·5H_2_O	pH 2–7	**SEM:** PEHA or DETA forms spherical SiO_2_ particles. After acidification, the SiO_2_ presents pores with sizes on the same order of magnitude as the polyamines, suggesting the acid removes the polyamines. Maximum removal occurred at pH ≤ 3.	Manning et al. (2017)^148^
**DETA**	–NR_2_ or −NR_3_^+^		5 min	**Simulations:** At pH 5, surface amines are removed. At pH ≤ 3, all polyamines are removed. Each polyamine only interacted with a single siloxide group. Interaction energies of individual molecules do not change with pH value.	
				At pH < 4, SiO_2_ is mostly neutral and interacts preferentially with water rather than PEHA or DETA. Methylated PEHA is 25% less likely to depart from the silica surface at lower pH values due to hydrophobic interactions.	

Synthetic macromolecular polyamines also show a pH-dependent influence upon silicification. Jantschke et al. found that the synthetic macromolecule PAH greatly increases solution turbidity at pH 6.8 but has almost no effect at pH 5.5 (Table 8).^40^ This pH dependence is attributed to a slightly lower total charge of the silica in solution and thus fewer charge–charge interactions between the PAH and the silica or silicic acid at lower pH.^40^ Also, the increasing positive charges on PAH with decreasing pH would prevent self-assembly (thus preventing the formation of hydrophobic regions) among these macromolecules due to repulsion between like charges.^40^ However, when Jantschke et al. added phosphate anions to the silicic acid/polyamine solutions at pH 5.5, the turbidity increased dramatically, suggesting that charge balance was restored and the amine groups could more efficiently sequester silica (Table 8).^40^

Manning et al. corroborate the suggestion that an interplay of macromolecular charge and pH limits the effects of polyamines on silicification.^148^ They found acidifying amine–silica structures at pH 4–5 removes polyamines from the surface of silica.^148^ Montagna et al. agreed with Manning et al. regarding the importance of charge–charge interactions between polyamines and silica/silanol groups.^56^ In a study (not featured in Table 8), Montagna et al. conducted molecular dynamics simulations along with NMR analyses and concluded that electrostatic interactions were the biggest factors in polyamine–silica composites.^56^

Hydrophobic groups also play a role in silicification via polyamines. Molecular dynamics simulations of methylated and unmethylated synthetic versions of PEHA polyamine show the more hydrophobic methylated version is 25% less likely to be removed from the surface of silica than its unmethylated counterpart, likely due to van der Waals interactions.^148^

To further investigate the effects of hydrophobicity and charge of amines on silicification, short-chain amines have also been methylated. Jantschke et al. determined the fully methylated cationic analogue of allylamine, allyltrimethylammonium bromide, increased the turbidity of a silica solution at a faster rate than its unmethylated counterpart, likely also due to hydrophobic effects and permanent charge (Table 8).^40^ Belton et al. also noted the probable influence of hydrophobicity on silicification and suggested the stability of hydrophobic microemulsions of polyamines may promote the formation of hollow or solid silica particles.^49^ Such an effect would correlate with the cation concentration, as aggregation is inhibited by charge–charge interactions.

When considering the impacts of hydrophobicity, recall Reaction 2 which depicts a representative silicification reaction with a water molecule produced as a byproduct. According to Le Chatelier’s principle, removing the product of a reversible reaction shifts the reaction equilibrium toward product formation.^149^ Therefore, greater hydrophobic regions around the silicic acid (with reduced local water molecules) could promote silicification by shifting the kinetic equilibrium toward products.

Overall, these studies demonstrate amines and polyamines promote silicification when they are charged, methylated, and in the presence of a phosphate counterion.^40,49,121,145,147^ This appears to be due to the charge balance and hydrophobicity.

### 4.3 Polysaccharide-Directed Silicification

Most studies regarding silica formation in the presence of polysaccharides are driven by potential for materials science applications, in contrast to biologically motivated protein and polyamine research. However, investigations of how native frustule polysaccharides influence biosilicification have proven to be difficult to conduct. The organic–inorganic separation techniques of biosilica require successive alkali extraction and deproteination steps before analytical characterization can take place.^150^ The low aqueous solubility of many polysaccharides, which leaves them in the AFIM (ammonium fluoride insoluble material) after protein extraction, also complicates their characterization and causes degradation of the original molecular structure.^19^ For this reason, current research on complex natural silicification-directing polysaccharides continues to be based almost exclusively on characterizations of monosaccharide composition.^19^ This may also contribute to the comparatively lower number of *in vitro* polysaccharide-directed silicification studies in comparison to *in vitro* protein-directed silicification investigations.

Three broad types of polysaccharides have been studied with respect to controlling silicification: cationic, anionic, and neutral polysaccharides. This framework organizes the discussion below and aids our analysis of polysaccharide structure–function relationships. Although many studies exhibit qualitative trends, future quantitative studies will be necessary to effectively characterize the kinetics and thermodynamics of the influence of polysaccharides on silicification systems.

#### 4.3.1 Cationic Polysaccharide-Directed Silicification

A limited number of studies use cationic polysaccharides, and this remains an area open for further research into composite biomaterials. Chitosan is perhaps the most important member of this group due to its similarities to the common biosilica polysaccharide, chitin. However, most of these studies are qualitative with insufficient characterization of the reaction conditions and materials (e.g., Table 9).^151,152^ Shchipunov et al. studied a variety of polysaccharides, including cationic polysaccharides, with respect to silica gelation for applications in food, drugs, or cosmetics.^151,153^ They suggest that, while charges may have an impact on silicification, hydrogen bonding likely drives the silicification process as evidenced by a lack of observed differences between silica products synthesized in the presence of anionic versus cationic polysaccharides.^151,153^ SEM images show the silica products form a smooth coating on the polysaccharide fibrils in contrast to aggregated silica spheres that form between these fibrils.^151,154^ These qualitative studies illustrate that polysaccharides influence silicification, but quantitative characterization techniques will be necessary to decipher the reaction processes.

**Table 9:** Summary of Silicification Studies Conducted with Cationic Polysaccharides with Conditions of pH, Time, Temperature, and Solvent

Polysaccharide Substrate	Selected Functional Groups	Source of Si Monomer	Conditions	Characterization Methods and Findings	Reference
**Chitosan** (DS(Ac) = 0.28)	–OH	THEOS	Ambient T	**FTIR,**^**13**^**C NMR and**^**29**^**Si NMR:** Suggests chitosan directs silicification via covalent C–O–Si bonds.	Bravo-Flores et al. (2021)^57^
	–NH_3_^+^ or −NH_2_	MeTHEOS	1% acetic acid		
	–NHAc				
**Chitosan** (DS(Ac) = 0.20, MW ≈ 290 kDa)	–OH	TEOS	pH 5–9	**N**_**2**_**Sorption, SEM, TEM:** Low chitosan concentrations at pH 5–6 form larger SiO_2_ nanoparticles with increased pore size, and smaller particles with decreased pore size at pH 6.5–8.5. High chitosan concentrations form SiO_2_ along the same trend. At pH 9, chitosan has no significant effects on SiO_2_ formation.	Witoon et al. (2012)^155^
	–NH_3_^+^ or −NH_2_		6 h at 40 °C, then 24 h at 60 °C		
	–NHAc		2% acetic acid buffered with 5 M NH_4_OH		
**Chitosan** (DS(Ac) = 0.20)	–OH	Na_2_Si_3_O_7_ (27 wt % SiO_2_, 4 wt % NaOH	pH 3, 5, 6	**Observations:** Precipitation is immediately observed at pH 6 but not at pH 3 or 5. Immediate precipitation at chitosan/SiO_2_ ratio of 0.4 at pH 3–6.	Witoon et al. (2011)^156^
	–NH_3_^+^ or −NH_2_		24 h at 40 °C, then 24 h at 100 °C	**TGA:** At chitosan/silica ratio >0.8, the precipitate phase separates into a silica-rich phase and a chitosan-rich phase.	
	–NHAc		2% acetic acid	**SEM:** Chitosan networks likely limit SiO_2_ particle sizes.	
				**N**_**2**_**Sorption:** The void size of chitosan networks decreases from pH 3 to 6.	
**Chitosan** (DS(Ac) = 0.24, MW ≈ 500 kDa) (DS(Ac) = 0.19, MW ≈ 200 kDa) (DS(Ac) = 0.14, MW ≈ 70 kDa) (DS(Ac) = 10, MW ≈ 20 kDa)	–OH	Na_2_SiO_3_ (0.82 wt % in 0.05 M sodium acetate)	pH 4–5.6	**β-Silicomolybdate:** Chitosan changes silicification rate from a 4th order reaction to a two-stage 1, 2, 3, or 5th order reaction. The presence of 200 kDa chitosan induces the largest rate constant of all chitosan samples.	Chang et al. (2006)^157^
	–NH_3_^+^ or −NH_2_		0–800 min	**Turbidity:** Aggregation rate increases 20–30× with the addition of chitosan.	
	–NHAc			**SEM:** SiO_2_ nanoparticles aggregate into clusters in the presence of chitosan.	
				**Elemental analysis:** SiO_2_ products contain 10% chitosan.	
				**Conclusions:** Likely −NH_3_^+^ and H-bonding −OH groups attract soluble silica species to facilitate polycondensation. Chitosan does not significantly increase the rate of polycondensation but does increase the aggregation of colloidal silica nanoparticles.	
**Chitosan** (DS(Ac) = 0.10)	–OH	Na_2_Si_3_O_7_ (27 wt % SiO_2_; 4 wt % NaOH)	pH 2–6	**N**_**2**_**Sorption/SEM/TEM:** Pore sizes increase with increasing pH.	Witoon et al. (2009)^158^
	–NH_3_^+^ or −NH_2_		24 h at 40 °C, then 24 h at 100 °C	**TGA/DTG:** More chitosan is incorporated into SiO_2_ at higher pH.	
	–NHAc		2% acetic acid	**Zeta potential:** Isoelectric points of SiO_2_-chitosan at pH 2, 3, and 4 are 4.74, 4.90, and 5.76. At higher pH, more chitosan molecules adsorb to the SiO_2_ particles’ surface with chitosan fully adsorbed at pH 5 and 6.	
**Chitosan** (DS(Ac) = 0.9, MW ≈ 13 kDa)	–OH	H_4_SiO_4_ (hydrolyzed TMOS)	pH 5.8	**Cryo-TEM/Cryo-ET:** Aggregated SiO_2_ spheres (≈15 nm diameter), then aggregated and tabular, and finally starfruit-like SiO_2_ structures form depending on incubation time of chitosan and phosphate ions. Suggests that phosphate ions promote chitosan aggregation and organizes spherical particles into star-fruit-like structures.	Leng et al. (2010)^159^
**NaH**_**2**_**PO**_**4**_	–NH_3_^+^ or −NH_2_		Ambient T	**EDX:** Phosphate ions were incorporated into SiO_2_.	
	–NHAc		4 h		
	H_2_PO_4_^–^		DI water		
**Chitosan**	–OH	H_4_SiO_4_ (hydrolyzed THEOS)	pH 5.5–6	**Observations:** Chitosan-silica produced an opalescent monolith hydrogel, and cat-HEC-silica produced a transparent monolith hydrogel.	Shchipunov et al. (2005)^154^
	–NH_3_^+^ or −NH_2_		≥1 week	**SEM:** Cross-linked cat-HEC (1.5 wt %) fibrils are covered by SiO_2_ (10 wt % THEOS) and surrounded by spherical SiO_2_ nanoparticles.	
	–NHAc		Ambient T	**Rheology:** Sol–gel transition of cat-HEC occurred when 0.5 wt % THEOS was silicified.	
**Cat-HEC**	–OH		Water		
	–N(CH_3_)_3_^+^				
**Cat-HEC** (MW = 950 kDa)	–OH	H_4_SiO_4_ (hydrolyzed THEOS)	Neutral pH	**Observations:** Transparent monolith structural features observed for cat-HEC-SiO_2_.	Shchipunov and Karpenko (2004)^151^
	–N(CH_3_)_3_^+^		Ambient T	**SEM:** Cat-HEC shows structure loosening and thicker filaments as wt % decreases or as cation functionalization decreases. Small spheres were observed.	
			Water	**Conclusions:** Lack of differences between cationic vs anionic polysaccharides suggests acceleration of silicification is due to hydroxy groups.	

**Figure 21:**
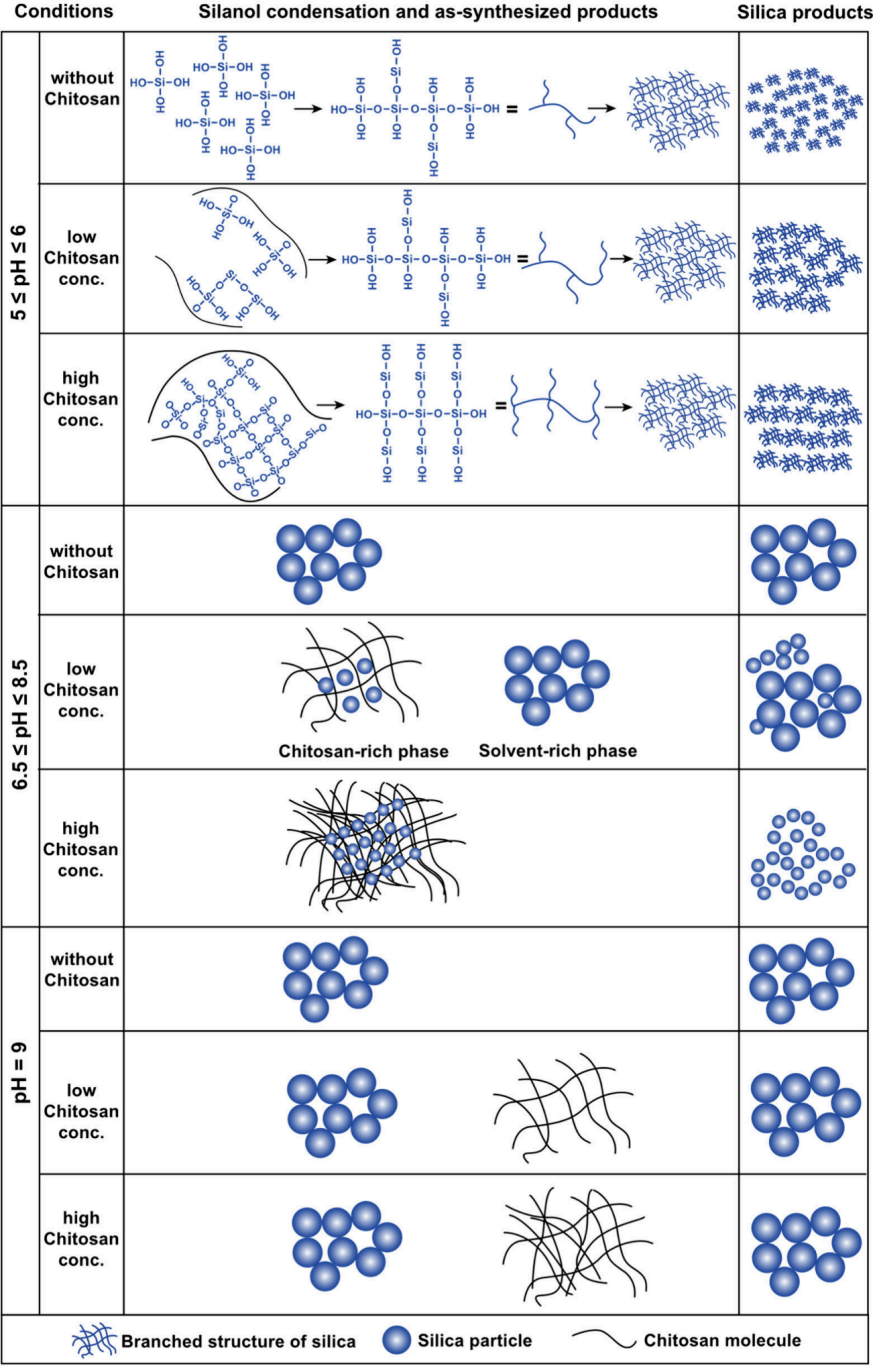
Depiction of how the chitosan concentration and solution pH affect silica formation. At pH 5–6, more of a gel-like structure is formed. Higher chitosan concentrations produce denser composite networks. At pH 6.5–8.5, large particles form in the absence of local chitosan and high local chitosan sterically hinders silica formation producing smaller particles. At pH 9, chitosan phase separates, leading to the unhindered formation of large particles (after Witoon et al., 2012).^155^

Witoon et al. provide a different perspective by coupling additional materials characterization with TEM and SEM observations of the products that form (Figure 21).^155,156,158^ Chitosan, with a degree of substitution (DS)(Ac) of 0.09–0.2, has a strong impact on silicification. In lower pH solutions (pH 5–6), larger silica particle sizes are formed and present larger pores. In contrast, higher pH values (pH 6.5–8.5) yield smaller silica particles with smaller pores (Table 9).^155,156,158^ The authors cite phase separation and chitosan sterically hindering silica formation as causes for the effects they observe. For example, at pH 6.5–8.5, they suggest chitosan becomes less water-soluble, thus phase separating and producing more tightly packed networks that physically hindered the growth of silica particles.^155^ Note that chitosan, like any polyelectrolyte, has a range of p*K*_a_ values that depend on chain length, the presence of salts, and other experimental conditions. Generally, the p*K*_a_ of chitosan is ∼6.2–6.8.^160^ With this in mind, one might further question the effects of positively charged chitosan C2 amino groups at lower pH on silicification in these systems.^155,156,158^ Witoon et al. characterized the DS(Ac) of the polymer but did not characterize chain length in all studies.^155,156,158^ Therefore, relationships between silicification and chitosan molecular weight remain unclear.

A relatively detailed study with regard to structure–property relationships investigated silica formation in the presence of chitosan materials with variable degree of polymerization (DP) and DS(Ac).^157^ Experiments conducted at pH 5.6 showed the rate of silica condensation increases with chitosan molecular weight.^157^ The resulting silica–chitosan nanoparticles (np’s) formed with an average diameter of 1.2 nm (1 h) and grew to 32 nm (24 h) (Table 9).^157^ The np’s subsequently aggregated to form clusters ∼20–30× faster in the presence of chitosan than the chitosan-free controls.^157^ Elemental analysis indicated the silica–chitosan composites contained ∼10% chitosan which approximated the initial weight ratio of chitosan and silicic acid in the reactant solutions.^157^ The authors proposed the linear cationic chitosan attracts silicic acid from solution through H-bonding with hydroxy groups on C3 and C6.^157^ They further postulated the attracted silica molecules subsequently provide a template for further silica synthesis.^157^

Using NMR spectra to evaluate silica–cationic polysaccharide systems, Bravo-Flores et al. reported Si–O–C bonds form between precursor THEOS and chitosan to promote silicification.^57^ It should be noted that the Si–O–C ^13^C NMR resonance which is presented as evidence for this interpretation is uncharacteristically narrow compared to the broad peaks typically seen in polysaccharide ^13^C NMR spectra.^57^

#### 4.3.2 Anionic Polysaccharide-Directed Silicification

Anionic polysaccharides have also been investigated as possible matrices for silicification due to possible applications for enzyme or cell encapsulation and gelling capabilities.^153^ Alginic acid or alginate is a polysaccharide with −COO^–^ or −COOH groups (p*K*_a_ ≈ 5) on the C6 position of its β-d-mannuronic (M) and α-l-guluronic (G) monosaccharides.^161^ Alginate is known for its complexation and gelation with Ca^2+^. Coradin et al. found via SEM that, at pH 7, alginate produced differently shaped silica np’s than the polysaccharide-free controls. They suggest alginate interferes with the assembly of nucleated particles due to charge–charge repulsion with any negatively charged silicates (Table 10).^161^ Alginate was not suspected to interfere with the initial nucleation process.^161^

**Table 10:** Summary of Silicification Studies Conducted with Anionic Polysaccharides under Conditions of pH, Time, Temperature, and Solvent

Polysaccharide Substrate	Selected Functional Groups	Source of Si Monomer	Conditions	Characterization Methods and Findings	Reference
**Alginate** (MW ≈ 150 kDa, 70% guluronic acid)	–COO^–^ or −COOH	Na_2_SiO_3_ (27 wt % SiO_2_, 14 wt % NaOH) or colloid silica (12 nm particle)	pH ≈ 7	**TGA:** All initial alginate was incorporated into the SiO_2_.	Coradin and Livage (2003)^161^
	–OH		Overnight	**SEM:** Mostly aggregates and a few spherical SiO_2_ particles. Lower alginate concentration produced larger aggregates of smaller silica particles. Higher alginate concentrations produced multiple particle sizes.	
			Tris-HCl buffer	**Conclusions:** No strong interactions between alginate and silica precursors are expected; the nucleation of primary SiO_2_ particles is not influenced by alginate. However, SiO_2_ particle assembly may be controlled by their limited diffusion in the viscous alginate.	
			Addition of CaCl_2_		
**Alginic acid** (MW ≈ 40 kDa, pI = 3.6–3.8 at pH 5)	–COO^–^ or −COOH	Na_2_SiO_3_ (27% SiO_2_, 10% NaOH)	pH ≈ 5	**Observations:** Immediate SiO_2_ precipitation observed at 37 °C with polymers (gelatin and gelatin+alginic acid) except pure alginate slowed the precipitation rate. Control forms a gel overnight.	Gautier et al. (2008)^124^
**Gelatin**	–OH		1 h at 37 °C then 20 °C for 1 day	**TGA:** Only small fraction of alginate associated with SiO_2_, contrasted with the total gelatin incorporation.	
			DI water	**TEM:** With alginate in solution, 5 nm SiO_2_ nanoparticles were produced.	
				**Conclusion:** Alginate helps to control SiO_2_ morphology.	
**Alginate** (MW n.d.)	–COO^–^ or −COOH	H_4_SiO_4_ (hydrolyzed THEOS)	Neutral pH	**Observations:** Turbid and syneresis or monolith structural features developed from SiO_2_-polysaccharide gels.	Shchipunov and Karpenko (2004)^151^
**κ-,ι-, and λ-carrageenans** (MW 700, 700, and 1024 kDa)	–OH		Ambient T	**SEM:** Aerogel with κ-carrageenan showed a different structure (fibrillar) than the C_9_H_24_ClNO_3_Si with 0.1 M sulfuric acid control (connected solid SiO_2_ particulates).	
**Xanthan** (MW n.d.)	–OSO_3_^–^		Water	**Conclusions:** Lack of differences between cationic vs anionic polysaccharides suggest acceleration of silicification is due to hydroxy groups.	
**κ-,ι-, and λ-carrageenans**^a^ (700 kDa, 700 kDa, 1024 kDa)	–OSO_3_^–^	H_4_SiO_4_ (hydrolyzed THEOS)	Neutral pH	**Observations:** Polysaccharides promoted silicification. Increasing SiO_2_ concentration increased brittleness and stiffness. Increasing polysaccharide concentration increased elasticity. Only κ-carrageenans caused syneresis.	Shchipunov (2003)^153^
	–OH		Ambient T	**SEM:** Cross-linked fibers decorated with ∼10–40 nm SiO_2_ particles	
			1 week to 9 months	**Rheology:** Carrageenans accelerated the sol–gel silicification kinetics.	
			DI water		

a κ-Carrageenan contained low-molecular-weight impurities (97 and 340 Da).

Gautier et al. compared the effects of alginate (a carboxylated, highly anionic polysaccharide) with gelatin (a highly cationic protein), the effects of which are discussed in Section 4.1.4 and Tables 7 and 10. This study also showed alginate did not interfere with the condensation of silica, but the composites that formed were 5 nm diameter spheres (nanoparticles) rather than gels.^124^ This suggests alginate acts as a flocculant. Gautier et al. further suggest there are weak interactions between the alginate and silicate, as evidenced by incorporation of only 10% of the initial polymer into the silica.^124^ In contrast, gelatin–silica composites formed instantaneously, producing 50 nm diameter particles.^124^ Overall, this study suggested cationic charges promote silica formation but both cationic and anionic macromolecules control silica morphology.

Shchipunov et al. found sulfated carrageenan polysaccharides accelerated the rate of the silica’s sol–gel transition (Table 10).^153^ Hydrogen-bonding was proposed as the likely cause of silicification promotion in polysaccharides because polyanions and polycations showed similar results.^151^ Among the studies highlighted here, the evidence suggests anionic polysaccharides have negligible effects on the onset of silica condensation but affect the subsequent growth and aggregation stages. However, these studies are mostly based on SEM and rheological analysis without a quantitative or mechanistic understanding of silica–anionic polysaccharide interactions.

#### 4.3.3 Neutral Polysaccharide-Directed Silicification

Neutral (uncharged) polysaccharides also have been investigated from a materials science perspective due to an array of applications from insulation to controlled drug delivery to gelling agents. As discussed in Sections 3.1.3, 3.2, and 3.3, neutral polysaccharides found in biosilicifiers, such as chitin and callose, are implicated in directing biosilicification. Moreover, the abundance of chitin in the frustule of diatoms and glass sponges (Sections 3.1.3, 3.2) continues to raise questions regarding the possibility of yet-unidentified roles in directing mineralization.

**Figure 22:**
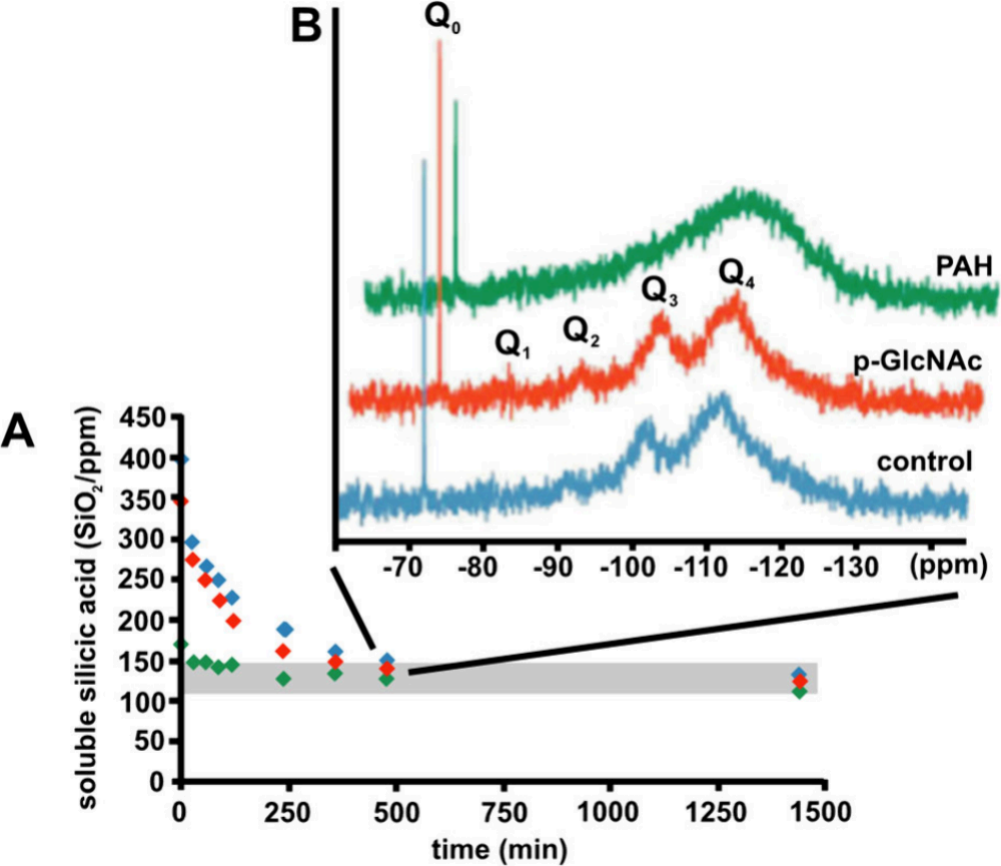
(A) Silicification rate experiments using the Molybdenum Blue method: Control without additive (blue), chitin (red), and PAH (green). (B) ^29^Si NMR spectra collected at 480 min of reaction time show the evolution of Q species in solution. Adapted from ref (162). Copyright 2011 American Chemical Society.

To the best of our knowledge, only one study has investigated the rate of silicification onto a chitin matrix. Spinde et al. used the molybdenum blue method to measure the rate of silicification under mild aqueous conditions and found rate is not significantly increased in the presence of β-chitin extracted from diatoms (Table 11).^162^ Parallel NMR characterizations of the silica species that form show many oligomers remain in solution without polymerizing after 480 min of reaction time (Figure 22).^162^ Corresponding ^13^C NMR analyses suggested β-chitin interacted with silicic acid via hydrogen bonding, but the strength of this interaction was insufficient to accelerate silicification.^162^ The effects of chitin in this study were compared to those of the highly cationic poly(allylamine hydrochloride), which appeared to significantly promote silicification and increase perturbations of the Si–O–Si bond in ^29^Si NMR.^162^ It is notable the team studied β-chitin rather than α-chitin, as the α-form is highly insoluble in aqueous single solvents due to its propensity for self-association (Figure 12). The molecular weight of the chitin used was not reported.

**Table 11:** Summary of Silicification Studies Conducted with Neutral Polysaccharides under Conditions of pH, Time, Temperature, and Solvent

Polysaccharide Substrate	Selected Functional Groups	Source of Si Monomer	Conditions	Characterization Methods and Findings	Reference
**β-chitin** (extracted from diatoms, *n* = 160, 15 kDa)	–NHCOCH_3_–OH	Na_2_SiO_3_	pH 5.5	**Molybdenum Blue:** Chitin does not increase the rate of silicification, in contrast to PAH.	Spinde et al. (2011)^162^
**PAH**			0.5–24 h	**Light, Fluorescence and Scanning Electron Microscopy:** β-Chitin is homogeneously embedded in the SiO_2_.	
			Ultrapure water	^**13**^**C NMR, Raman, and ^29^Si MAS NMR:** Confirm interfacial interactions between chitin −OH groups and SiO_2_; suggest Si–O–Si bond angle is less perturbed by chitin than PAH.	
**α-Chitin** (extracted from *Ianthella basta*)	–NHCOCH_3_–OH	TEOS	1 h at Ambient T or 120 °C or 20 h at 37 °C	**EDX:** More SiO_2_ formed via higher temperature method.	Wysokowski et al. (2013)^163^
			EtOH and NH_3_ solutions	**SEM:** Homogenous distribution of spherical SiO_2_ particles on chitin and greater coating at high temperatures.	
				**FTIR:** 474 cm^–1^ peak corresponds to the deformation vibrations of Si–O–C bond.	
				**Conclusion:** Silica preferentially interacts with chitin via hydrogen bonding with hydroxy groups and carbonyl groups.	
**HEC**	–OH	H_4_SiO_4_ (hydrolyzed THEOS)	pH 5.5–6	**Observations:** Cyclodextrins have strong catalytic effects on SiO_2_ sol–gel processing.	Shchipunov et al. (2005)^154^
**Laminaran**	–OR		≥1 week	**Rheology:** Silica sol–gel transitions occur with arabinogalactan with ∼5 wt % THEOS, α- and β-cyclodextrin with ∼4 wt % THEOS, and locust bean gum with ∼5 wt % THEOS.	
**Arabinogalactan**			Ambient T		
**α- and β-cyclodextrin**			Water		
**Locust bean gum**					
**Guar gum**					
**Hydroxypropyl guar gum** (HPGG)	–OH	THEOS	Ambient T	**Rheological Measurements:** Induction period occurred. HPGG decreases sol–gel transition time.	Wang and Zhang (2007)^164^
	–OR		Water	**SEM:** Network of crossed/branched filaments and structure of connected SiO_2_ particles (looser network with more spherical silica in previous work)	
				**Conclusions:** Suggests −OH bonding for silicification catalysis.	
**Cellulose nanofibril** (CNF)	–OH	H_4_SiO_4_ (hydrolyzed TEOS)	pH 8–12	**SEM:** SiO_2_ control had dense aggregates of silica spheres in a “pearl necklace structure” while composite had spherical particles coating CNF fibrils	Fu et al. (2016)^29^
			10 min	**Bulk Density and Si Content:** Bulk density increased from 0.059 g/cm^3^ to 0.295 g/cm^3^ and Si content increased from 3.8 wt % to 79.5 wt % as pH increased from 8 to 12.	
			Ethanol and water	**FTIR:** CNF hydroxy peak decreased with increasing SiO_2_ and basicity, potentially due to SiO_2_ binding to hydroxy oxygen, releasing the proton.	
				**Observation:** Higher pH correlated with faster silicification.	

**Figure 23:**
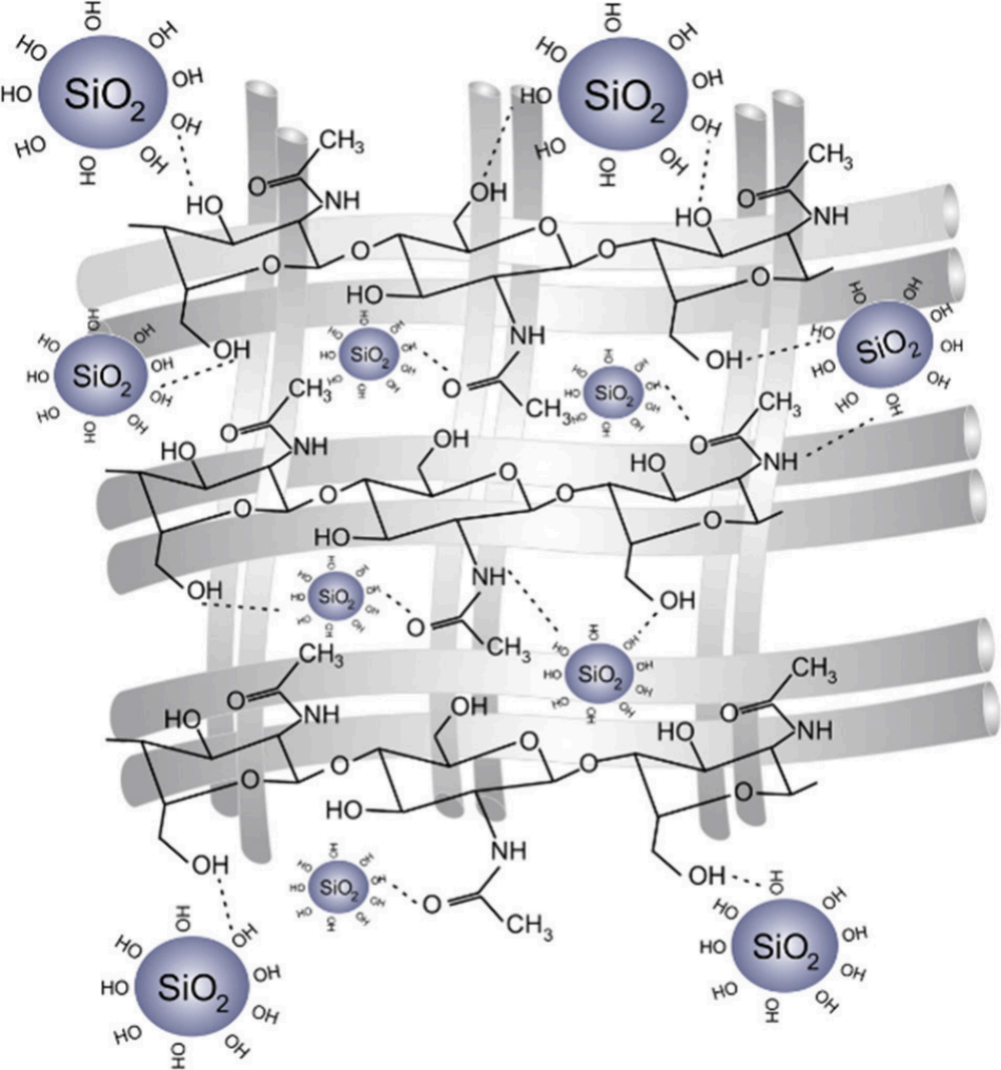
Simplified depiction of chitin–silica interactions. Hydrogen bonds are shown between the silica hydroxy groups and the hydroxy and amide groups on chitin. Reproduced with permission from ref (163). Copyright 2013 Elsevier S.A.

Another study by Wysokowski et al. investigated silicification onto an insoluble matrix of α-chitin (Figure 23).^163^ They suggested silica preferentially interacts with chitin via hydrogen bonding.^163^ Unfortunately, due to the stark differences between reaction conditions and characterization methods by Wysokowski et al. and Spinde et al.,^162^ the data cannot be compared to probe the impact of polysaccharide folding on the silicification rate (Table 11). In addition, Wysokowski et al. provided thorough characterization of the product but did not provide kinetic data.^163^ While Spinde et al. utilized aqueous conditions, Wysokowski et al. followed industrial conditions of ethanol, ammonia, and water solutions and elevated temperatures of 37° or 120 °C (Table 11).^163^ Both chitin-based studies suggest that hydrogen bonding takes place between silanol groups and chitin.

A number of other studies investigating neutral polysaccharides argued that hydrogen bonding occurs between the polymer hydroxy groups and the hydroxy groups of silicic acid.^29,164^ Some, such as Shchipunov et al., suggested hydrogen bonding accelerates silicification. This hypothesis was based solely on SEM observations, and kinetic data was not obtained.^28^ The team in 2005 suggested cyclodextrins had strong catalytic effects on silicification, but this was not further elucidated (Table 11).^154^ Hydroxypropyl guar gum was investigated by Wang et al. and through rheological measurements; they found this polysaccharide decreased the time to reach the sol–gel transition, likely facilitated by hydrogen bonding (Table 11).^164^

Hydrogen bonding is a common theme in discussions of polysaccharide influences on silicic acid condensation. While it is very likely that hydrogen bonding does play a role, there is minimal understanding as to what that role is and how other variables might influence it. Hydrogen bonding is a directional force that relies on factors such as stereochemistry and orientation. Thus, chain conformation and self-association of polysaccharides, for example, affect hydrogen bonding abilities with both the silicic acid and water molecules. More research and characterization of polysaccharides are required to fully understand how hydrogen bonding might affect silicic acid binding and condensation.

### 4.4 Other Organic Model Systems for Silicification

#### 4.4.1 Amine and Carboxyl Group-Focused Silicification

Systematic studies that quantified the rate of silica nucleation in the presence of amine and carboxyl groups have also been performed. Experimental measurements of silica nucleation rates in a series of amino acid solutions (all amino acids contain carboxyl and amine groups) shows that all organic acids decrease the induction time to condensation at all supersaturations. Analysis of the rate data using Classical Nucleation Theory and the Makrides-Turner-Slaughter equation suggests amino acids promote silicification by lowering the kinetic barrier to nucleation (Table 12), while the thermodynamic barrier to nucleation is unaffected (see Sections 5.1.1 and 5.1.2).^165^ Using this mathematical model, the relationship between the free energy of adsorption and the kinetic barrier shows silica nucleation rate is faster in the presence of lysine and arginine compared to glycine.^165^ Overall, amino acids reduced the kinetic energy barrier to nucleation in proportion to their net positive charge, suggesting that ionic interactions have the strongest control over silicification. This trend of charge-promoted silicification was explored using citric acid, which is highly anionic with three carboxylate groups. The measurements showed citric acid most strongly enhanced the rate of silicification.^165^

**Table 12:** Summary of Silicification Studies Conducted with Model Systems with Conditions of pH, Time, Temperature, and Solvent

Substrate	Selected Functional Groups	Source of Si Monomer	Conditions	Characterization Methods and Findings	Reference
**11-mercaptoundecanoic acid** (grafted to gold matrix)	–NH_2_ or −NH_3_^+^	H_4_SiO_4_ (hydrolyzed TMOS)	pH 5	**AFM:** The rate of silica nucleation is ∼18× faster on NH_3_^+^/COO^–^ surfaces than on COO^–^ surfaces alone. Amine terminated surfaces alone failed to induce surface nucleation.	Wallace et al. (2009)^51^
**11-amino-1-undecanethiol hydrochloride** (grafted to gold matrix)	–COO^–^		Ultrapure water with 0.1 M NaCl	The increase in nuclei seen on carboxyl and amine hybrid surfaces was confirmed when these areas exhibited more nuclei than other areas of a patterned surface.	
**Orthophosphate**	H_2_PO_4_^–^		Flow through method kept supersaturation constant	In the presence of amines and orthophosphate, silica formation occurred at a lower nucleation site density than NH_3_^+^/COO^–^ surfaces.	
**Amino acids**	A large variety of functional groups	H_4_SiO_4_ (hydrolyzed TMOS)	pH 5	**β-Silicomolybdate:** All solutions showed a lag/induction time. At a lower silicic acid supersaturation state, the induction time increased. All organic acids decreased the induction time for all supersaturations. Amino acids promote rate in direct proportion to their net positive charge. These findings suggest that H-bonds play a role in silicification but ionic interactions have the strongest controls. Anionic citric acid does not fit the net trend observed for cationic charges.	Dove et al. (2019)^165^
**Citric acid**			20 °C	**MTS Model:** For the NaCl control, interfacial free energy was ≈54.9 mJ m^–2^. At higher NaCl concentration (0.10 to 0.70 M), interfacial free energy decreased to ≈51.4 mJ m^–2^; therefore, NaCl affects the rate of silicification through reductions in the interfacial free energy. A critical nucleus radius was estimated to be 5.1–7.8 × 10^–8^ cm. NaCl lowers thermodynamic barrier to nucleation likely by stabilizing charged, reactive species. Amino acids and citric acid reduce the kinetic barrier to nucleation, with a range of –1009 ± 169 J mol^–1^ (alanine) to –1690 ± 96 J mol^–1^ (citric acid).	
**NaCl**			Ultrapure water with 0.10 or 0.70 M NaCl		
**Potassium**d**-gluconate**	–OH	SiO_2_ solution	Alkaline solution	^**29**^**Si NMR:** Two binomial septets appear in the region of hexaoxosilicon centers (−140.8 and −141.5 ppm) when silicon is in the presence of monopotassium d-saccharic acid. An increase in polyol concentration and/or pH or a decrease in temperature favors hexa-coordinated Si over penta- or tetra- coordinated species.	Kinrade et al. (2001)^166^
**Monopotassium**d**-saccharic acid**			270–300 K	^**13**^**C NMR:** Si coordinates at the hydroxy groups flanking the *threo* pair. Integrating intensities indicates that each organosilicon complex contains exactly three polyol molecules per silicon center.	
				**Conclusions:** Threo configuration strongly interacts with silicate anions to produce stable penta- or hexa-coordinated SiO_2_	
**Lignin** (from mature rice straw)	–OH highly aromatic	Na_2_SiO_3_	pH 6.4 or 5.4	**TEM, EDXA:** Silica formation was seen in lignin+borax solutions but not in lignin+DMSO solutions. Suggests macromolecular lignin, but not its moieties, induces silica deposition in plants.	Fang and Ma (2006)^167^
			DMSO		
			pH 9.11–10.05		
			Borax aqueous solution		
**Mannose**	–OH	H_4_SiO_4_ (hydrolyzed TMOS)	pH 6.8	**SEM:** The PAH and phosphate system produced 200 nm diameter particles. Addition of mannose produced no significant changes. The addition of phosphorylated mannose increased particle diameters to ≈700 nm. All additives produce spherical, smooth surface nanoparticles.	Hedrich et al. (2013)^78^
**α-**d**-mannopyranose-1-phosphate**, **3-O-Me-β-mannopyranose-1-phosphate** (extracted from diatoms)	–HPO_4_^–^		12 min		
**PAH**	–NH_2_ or −NH_3_^+^		BIS-TRIS propane/HCl buffer solution		
**Sodium phosphate**	H_2_PO_4_^–^				
**PEG** (1550–20 000 Da)	R–O–R	Na_2_SiO_3_·5H_2_O	pH 7–8	**Molybdenum Blue:** Larger MW PEG results in more molybdate-reactive silica in solution, until the PEG has a molecular weight of ≥10 000 Da, where no more than 360–370 ppm of silicic acid is stabilized. The amount of molybdate-reactive silicic acid also reaches a maximum at a certain concentration of PEG.	Preari et al. (2014)^101^
			1–72 h	^**29**^**Si NMR:** Only mono- and disilicic acid (*Q*_0_ and *Q*_1_) peaks are observed.	
				^**1**^**H NMR and ^2^D HETCOR:** Proton shift from 6 to 7 ppm indicates stronger hydrogen bonding in the presence of PEG.	
				**FTIR:** PEG O atom band positions and vibrations are perturbed in the presence of silica species.	
**Human Enterovirus Type 71** (EV71, a nonenveloped picornavirus)		Na_2_SiO_3_	pH: 5.5–6.5	**TEM:** The cationic rich regions of the EV71 acted as nucleation sites silicification. SiO_2_ protected the from significant thermal damage.	Wang et al. (2015)^168^
			15–30 min	**Raman Spectroscopy and EDX:** Indicate presence of amorphous SiO_2_ around EV71.	
**Choline**	–OH	Na_2_SiO_3_	pH 7	^**13**^**C–^29^Si CP-REDOR and Molecular Dynamics Simulations:** Tightly and loosely bound choline molecules are detectable on SiO_2_ surfaces. As ionized SiOH groups increase, it is likely that both H-bonding and electrostatics play a role in choline-silica interactions.	Brückner et al. (2016)^169^
	–N(CH_3_)_3_^+^		24 h		
			ultrapure water		
**PEI**	–NH_2_ or −NH_3_^+^	Na_2_SiO_3_·5H_2_O	pH 7	**β-Silicomolybdate Method:**PEI inhibits silica formation with 53 ppm more silicic acid than the control after 72 h. PPEI has even greater inhibitory effects: >200 ppm soluble silica than the control was left in solution after 24 h (dropping after 48 and 72 h). Linear inhibition of silicification seen with increasing phosphomethylation with the fully grafted PPEI causing the highest inhibition.	Spinthaki et al. (2016)^170^
**PPEI** (zwitterionic phosphonated analog of PEI)	–NR_2_		0–72 h		
	–PO_3_H^–^				
**PVA** (MW 1100–31 000; 57 000–66 000; 88 000–97 000 Da)	–OH	Na_2_SiO_3_·5H_2_O	pH 6–8	**β-Silicomolybdate Method:** The concentration of molybdate-reactive silica did not change in the presence of PVA compared to the control. The concentration of molybdate reactive silica increased in the presence of PEG in comparison to the control.	Korhatzis et al. (2022)^171^
**PEG** (MW 1550–20 000 Da)	R–O–R		0–72 h	**Computational**: PVA self-associates and does not interact with silicic acid molecules. The ether groups of PEG are shown to hydrogen bond with the −OH groups on silicic acid, which helps to dissociate silicic acid monomers from one another. In a solution of PVA, PEG, and silicic acid, the PVA preferentially hydrogen bonds to the PEG and prevents it from stabilizing the silicic acid.	
			Ambient T		

**Figure 24:**
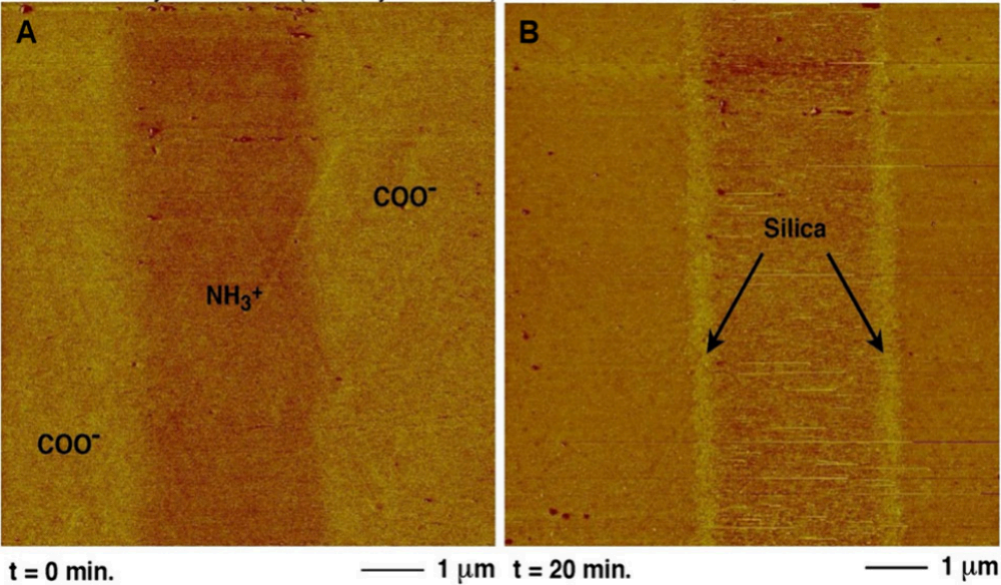
AFM investigation of silica precipitation onto patterned surface with alternating stripes of carboxyl- and amine-functional groups (A) before treatment and (B) after silicification. Most silica is deposited at the interface between carboxyl and amine groups. Conditions for this work are pH 5, σ = 2.14, and *T* = 25 °C. Reproduced from ref (51). Copyright 2009 American Chemical Society.

In another quantitative study, silica was nucleated onto gold substrates functionalized with carboxyl-terminated molecules, amine-terminated molecules, or both.^51^ Using a flow-through cell to hold the monosilicic acid concentration (supersaturation) constant, the team conducted an *in situ* atomic force microscopy (AFM) study of silica nucleation on these functionalized surfaces. Wallace et al. found the rate of nucleation is strongly promoted by the presence of both amine and carboxylate groups.^51^ By measuring nucleation events over time for a series of constant chemical driving force conditions, they calculated variables proportional to energy barriers to nucleation (see Section 4.5, Figure 24, and Table 12).^51^ Surfaces functionalized with both NH_3_^+^ and COO^–^ groups promote the rate of silicification ∼18× compared to COO^–^ surfaces alone (Figure 24, Table 12).^51^ Surfaces functionalized solely with amines failed to induce a measurable rate of surface nucleation.^51^ By directly measuring the rate of nucleation via an *in situ* method, they were able to discern energetic barriers that could not otherwise be determined via SEM or other qualitative techniques.

#### 4.4.2 Additional Functional Group-Focused Studies

The effects of specific functional groups on silicification have been investigated using natural and synthetic macromolecular and small molecule model systems. Two studies determined that synthetic macromolecules decreased the rate of silicification. Preari et al. found that higher molecular weights of the ether-rich polymer PEG slow the rate of silicification (Table 12).^101^^29^Si NMR showed only the presence of the monomer (Q_0_) and dimer (Q_1_).^101^ Subsequent NMR methods indicated a possible interaction between the ether species and the silicic acid species.^101^ This observation suggests ether-based hydrogen bonding plays a role in inhibiting silicification. Spinthaki et al. used polyethylene imine (PEI) to test the effects of amines on silicification and found that, at pH 7, PEI slowed silicic acid condensation (Table 12).^120^ When PEI was converted to a zwitterion by functionalization with phosphate groups, the polymer further decreased the rate of silicic acid condensation.^120^ This result sharply contrasts with multiple studies that report highly charged molecules, particularly zwitterions, promote silicification.^68,85,165^ It is possible the branching or self-association or the charge balance/distribution of phosphorylated PEI differentiates this model from previous studies. In addition, this study was mostly conducted at pH 7 rather than pH 5.^120^

Small molecules were also studied as model systems for silicification. Brückner et al. used choline to investigate the effects of cationic amine groups and hydrogen bonding on silicification.^169^ Both the cationic amines and hydroxy functional groups played roles in promoting silicification (Table 12).^169^ This was supported by solid state NMR investigations and molecular dynamics simulations as well.^169^ Using these technologies, they found both electrostatic interactions and hydrogen bonding at the organic–inorganic interface greatly depend on the hydration level and charge of the silica surface.^169^ For example, in the dried and partially ionized state, hydrogen bonding more tightly bound the choline to silica.^169^

Kinrade et al. hypothesized the orientation of hydroxy groups influenced silicification.^166^ To test this hypothesis, the team induced silicification with monosaccharides in solution.^166^ The team analyzed these experiments using solution state ^13^C and ^29^Si NMR and found that Si–O–C bonds were formed through condensation with the hydroxy groups flanking the *threo* pair of the monosaccharides (Table 12).^166^ However, ^13^C–^29^Si HMBC NMR was not performed, which would have been useful in confirming this hypothesis. When the sugars were acidified into their open chain forms or “sugar acids”, these carboxyl-containing chains dramatically enhanced silicification.^166^ Overall, more information is needed to understand the effects of the hydroxy group orientation on silica. Hedrich et al. tested the effects of mannose and mannopyranose-1-phosphate monosaccharides on silica formation and found via SEM that the presence of phosphate groups led to formation of larger particles (Table 12).^78^ While hydroxy groups may affect silicification, studies continue to report that charged groups have stronger influence over silicic acid condensation.^125,165,169^

DNA-templated silicification has been increasingly studied, particularly with regard to DNA origami-templated silica condensation. This area of research is intriguing and growing, but this Review is focused on macromolecule–silica interactions under aqueous conditions. In these systems, the silicification methods rely upon either the nonaqueous Sẗober method or the mixing of *N*-trimethoxysilylpropyl-*N,N,N*-trimethylammonium chloride (TMAPS) or 3-aminopropyl triethoxysilane (APTES) with a silica precursor such as TEOS to polymerize the silica with charged moieties to adhere to the DNA.^172^ To explore this field, we refer the reader to a number of excellent studies on related topics.^172−175^

### 4.5 Cooperative Interactions in Biosilicification?

From silaffins and polyamines to functionalized substrates, studies repeatedly suggest the importance of cooperativity between ions and macromolecules in silicification. However, quantitative evidence is limited. Understanding the mechanisms by which cooperative interactions promote silicification is a frontier area that will require complementary experimental and computational approaches. As discussed in Section 4.4.1, the study by Wallace et al. that measured the kinetics of silicification onto carboxyl- and amine-grafted surfaces provides evidence of cooperative interactions between functional groups (Figure 24).^51^ Surfaces with both amine and carboxylate groups increase nucleation rate ∼18× compared to those with only carboxylate groups.^51^ The differences raise a number of questions regarding the mechanisms and thermodynamic versus kinetic drivers by which ion cooperative interactions can be tuned to promote silicification.

Other quantitative crystal nucleation studies of cooperative ionic interactions may be used for inspiration and insights into future studies of silicification. Nielsen et al. investigated the influence of functional groups on CaCO_3_ nucleation using an amphiphilic diblock-polypeptoid where the hydrophilic block contained carboxyl- and amine-functionalized residues.^176^ They showed the peptoid-functionalized substrate presented a significantly lower barrier to nucleation than carboxyl- or amine-terminated SAMs alone.^176^ The calcium carbonate nucleation rate was higher on a 1:1 carboxyl:amine functionalized SAM, illustrating the significance of ionic cooperation.^176^ Similarly, Hamm et al. reconciled disparate views of template-directed calcite nucleation using SAMs as templates. The team found that both stereochemical matching of organic molecules guides nucleation and good binding strength equates to promotion of nucleation as interfacial free energies correlated to the free energy of binding.^177^ Using quantitative methods to understand the energetic driving forces of silicification, these studies show how cooperativity between molecules and ions could be active in directing silicification.

## 5 Toward a Mechanistic Understanding of Silicification

With recent advances in biopolymer synthesis, it is now possible to quantitatively address ongoing uncertainties regarding the role(s) of macromolecules in silicification. The extensive silica nucleation literature provides a general guide with qualitative experimental insights that include the dependence on solution pH, temperature, and supersaturation as well as molecular composition, solubility, and purity (Tables 4–12). However, Sections 3 and 4 highlight multiple contradictions regarding the types of biomolecules and conditions that have the strongest influences on silicification. For example, amine groups are reported to inhibit silicification in some chemical environments,^49,51,121,145^ while in other conditions amines promote silica condensation.^31,49,146,165^ The effects of the cooperation between opposing charges on silicification also continue to be actively debated, particularly regarding phosphate–amine interactions.^40,51,65,68,78,106,120,121,145,159,170,178^ Literature is also contradictory with regard to arguments for or against other driving factors of silicification including hydrophobicity^40,49,106,125,148^ and hydrogen bonding.^100,101,104,125,151,153,162,164,179,180^ It is remarkable that many additional areas in which macromolecules have the potential to influence silicification are simply unstudied; including very little research into higher order interactions between organic molecules and silicic acid species or silica.

To decipher the macromolecule–silica interactions that control nucleation and build a quantitative and comprehensive understanding, future studies must adhere to two standards. First, the organic substrates used in these studies must be thoroughly characterized. Higher order structure, molecular weight, and charge concentration are a few examples of characteristics that likely have profound impacts on silicification. We cannot seek to understand the influence of these factors, individually or iteratively, and build comprehensive physical models without this critical information. The technology used to specifically tune and mimic these crucial characteristics for protein and peptide research has existed for decades, while techniques to tune these characteristics in polysaccharides have only recently begun to develop and remain difficult.

Second, rates of silicification must be quantified and analyzed to resolve the thermodynamic versus kinetic energy barriers that drive nucleation. By establishing relationships between rate and driving force, we can use theoretical constructs to obtain the thermodynamic barriers and kinetic prefactors for the reaction. With the fundamental approaches suggested here, it will become possible to finally build the underlying principles of silicification and develop a “Rosetta Stone” that accelerates translations to diverse applications for natural systems and controlled synthesis of new materials.

### 5.1 Opportunity to Build Quantitative Model of Biosilicification

To demonstrate the potential of quantitative approaches for deciphering biosilicification, we first highlight the relationships contained in Classical Nucleation Theory (CNT) and then introduce the Makrides–Turner–Slaughter (MTS) model that is used in studies of silica condensation.^181^ CNT was first developed to describe the energy barrier to forming amorphous materials^182^ and provides a useful framework for resolving the kinetic and thermodynamic contributions to reaction rate.^181^ Detailed derivations of CNT and MTS as well as their applications are found elsewhere.^165,183,184^

#### 5.1.1 Classical Nucleation Theory

Nucleation occurs when the energy of bond formation overcomes the cost of creating a new interface, the interfacial free energy, to form a stable embryo. Classical Nucleation Theory states that the flux or steady state nucleation rate (*J*, cm^–3^s^–1^) of homogeneous or heterogeneous crystal formation from a supersaturated aqueous solution is determined by two energetic parameters: the thermodynamic barrier (Δ*g*_*c*_, Joules (J)) and the kinetic barrier, or activation energy (*E*_*a*_, J),^181,185,186^ such that
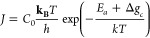
4where *C*_0_ is the initial concentration of nucleating species in solution, **k**_**B**_ is the Boltzmann constant (J K^−1^), *T* is temperature (K), and *h* is Planck’s constant (Js). The thermodynamic barrier to nucleation describes the excess free energy required to create a newly formed phase of critical radius (*r*_*c*_), while the kinetic energy barrier describes the activation energy associated with the transfer of a molecule from a solution to the surface (desolvation and attachment) and/or structural rearrangement within the nucleus before or during the phase separation necessary to form a critical nucleus.^181,185,186^

**Figure 25:**
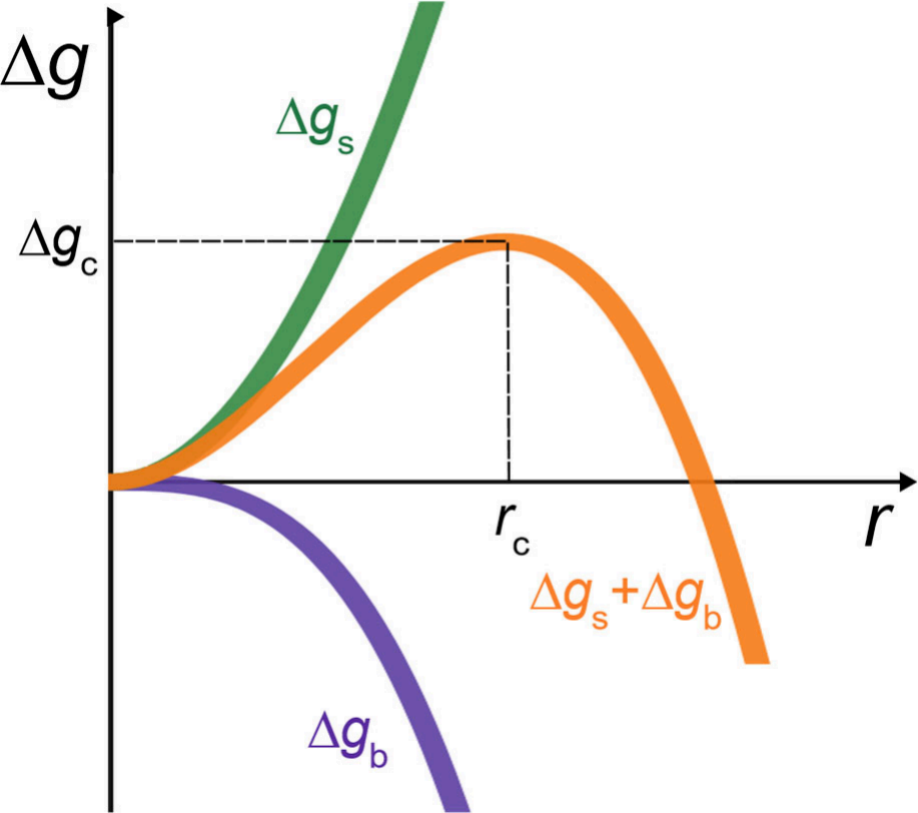
Representation of the free energy of nucleus formation per molecule (Δ*g*_*s*_ + Δ*g*_*b*_, orange) surface free energy change per molecule (Δ*g*_*s*_, green), and bulk free energy change per molecule (Δ*g*_*b*_, purple) versus critical radius, *r*_*c*_.

The thermodynamic barrier to nucleation can be described by first considering the free energy of formation per molecule (Δ*g*) to form a spherical embryo with *r*_*c*_ that is given by^5^
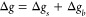
5
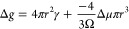
6where Δ*g*_*s*_ is the surface free energy change per molecule, Δ*g*_*b*_ is the bulk free energy change per molecule, γ is the interfacial free energy between the solution and critical nucleus (in mJ m^−2^), Ω is the volume per molecule (for silica in the solid phase, this is ≈4.5 × 10^–23^ cm^3^),^165^ and Δμ is the chemical potential of nucleating species. Δ*g*_*s*_ varies as a function of *r*^2^, and Δ*g*_*b*_ varies as a function of *r*^3^. These, along with the overall function Δ*g*, obey the relations shown in Figure 25.^187^ Taking the first derivative of Δ*g* (eq 6) with respect to *r* and setting this equation equal to zero  obtains the radius at which Δ*g* is at its maximum, which is termed the critical radius (*r*_*c*_)^5^
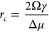
7

In addition, the chemical potential of nucleating species (Δμ) is often represented by
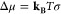
8where **k**_**B**_ is the Boltzmann constant (J K^−1^), *T* is temperature (K), and σ is the supersaturation. Supersaturation (σ) can also be written as
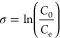
9where *C*_e_ is the concentration of H_4_SiO_4_° in equilibrium with respect to the bulk solubility of amorphous silica (≈1.93 mM at 25 °C).^188^ Substituting eqs 7 and 8 into eq 6 and simplifying obtains the maximum free energy of nucleation or the energy at the critical nucleus (Δ*g*_*c*_) for a single molecule:
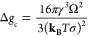
10Equations 7 and 8 combine into an expression of the Gibbs–Thomson relation, which shows the dependence of critical nucleus size on the chemical driving force (supersaturation, σ) and interfacial free energy (γ); stable particle size decreases with increasing σ or with decreasing γ. The critical radius (*r*_*c*_) and critical free energy of nucleation (Δ*g*_*c*_) are also depicted in Figure 25. Substituting eq 10 into eq 4 and rearranging produces the steady state rate of nucleation (*J*), which is given by^189^
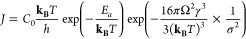
11Equation 11 is simplified by collecting terms to define:
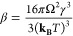
12and
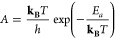
13to obtain
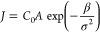
14where *A* (s^–1^) is the kinetic constant for forming a critical nucleus and β contains a thermodynamic constant for creating a new interface during nucleation. From this linear form, the thermodynamic and kinetic barriers to nucleation can be estimated from β and *A*, respectively, using rate data (*J*), the initial concentration of silicic acid (*C*_0_), and supersaturation (σ).

#### 5.1.2 Makrides-Turner-Slaughter Nucleation Theory

To quantify the barriers to silicification, the Makrides-Turner-Slaughter (MTS) method was developed in 1980 based on classical nucleation theory to evaluate silica condensation from brine solutions.^181^ It is an established method based on induction time (τ, s) or the period of time during which critical nuclei are formed. During the induction period, the amount of silicic acid removed from solution is below the detection limit of most analytical techniques. This theory assumes (1) particles are forming or fluctuating continuously throughout the induction time until stable nuclei of critical size are formed, and (2) the majority of nuclei have formed by the end of the induction time.^181^ The derivation (described in Makrides et al., 1980^181^) obtains a relationship between the induction time (τ) and initial silicic acid concentration (*C*_0_):
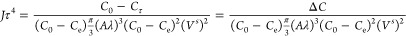
15where *C*_τ_ is the silicic acid concentration at the end of the stable period, *C*_e_ is the equilibrium concentration of silicic acid in solution, Δ*C* is the detection limit for silicic acid concentration, λ is the molecular diameter of a silica molecule (3 × 10^–8^ cm); *V*^*s*^ is the molar volume of the solid precipitate (Ω) times Avogadro’s number (*V*^*s*^ = Ω*N* = 27.09 cm^3^ mol^–1^), *J* is the flux or nucleation rate per unit volume, and *A* is given by eq 13.^165^

#### 5.1.3 Evaluating the Experimental Data

To fit the MTS model to rate data, eq 14 is substituted into eq 15 and rewritten to obtain the relationship between τ and σ such that
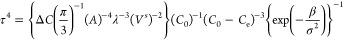
16This expression can be simplified into the form:
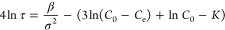
17where
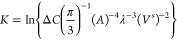
18Equation 17 predicts an inverse, linear relationship between induction time and supersaturation. Estimates of β are obtained from the slope of 4 ln τ versus  while *A* is evaluated using a second derivative statistical test (such as JMP, SAS Institute). Substituting β into eq 12 yields the interfacial free energy, γ for the corresponding experimental conditions. Broad application of this equation to silica nucleation data could revolutionize our mechanistic understanding of silicification because, with only a handful of experimental parameters, we are able to estimate fundamental thermodynamic and kinetic parameters of silicification.

### 5.2 Proof of Concept

**Figure 26:**
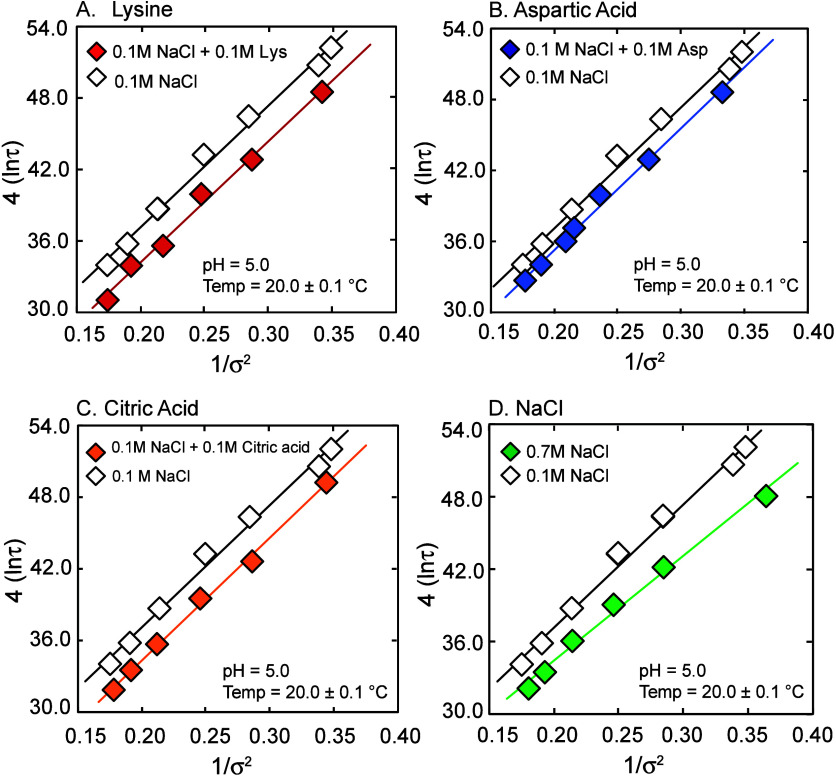
Measurements of silica nucleation induction times estimated per the Makrides-Turner-Slaughter (MTS) model as the natural log of the induction time (τ) versus the reciprocal of supersaturation squared (1/σ^2^). Fitting the MTS model to the data, the interfacial free energy (thermodynamic barrier) is extracted from the slope, and the kinetic barrier to silicification is determined from the *y*-intercept. Control experiments are represented by open diamonds, and filled diamonds represent the additive treatments. Organic acids reduce the kinetic barrier to silicification without affecting reaction thermodynamics: (A) Lysine, (B) Aspartic acid, and (C) Citric acid. In contrast, NaCl (D) enhances the rate by decreasing the thermodynamic barrier to nucleation (see also Figure 25) (after Dove et al., 2019).^165^

A study of silica polymerization rates in solutions containing a series of amino acids (and citric acid) demonstrates the quantitative information that can be obtained using the MTS approach.^165^ By fitting the MTS model (eq 17) to measurements of induction time (τ) at 20 °C for a series of supersaturated solutions, Dove and coauthors estimated the thermodynamic barrier (Δ*g*_*c*_), interfacial free energy (γ), and kinetic barrier (*E*_*a*_) for silica nucleation in a series of different amino acid solutions at variable ionic strength. The approach found that NaCl and organic acids modify the rate of silica nucleation through thermodynamic and kinetic factors, respectively.^165^ The introduction of organic acids increased rate through biomolecule-specific reductions in *E*_*a*_.^165^ For example, lysine reduces the *E*_*a*_*lysine*__ by ≈1685 ± 315 × 10^3^ J·mol^–1^ and citric acid, the *E*_*a*_*citric*__ by ≈1690.7 ± 96 × 10^3^ J·mol^–1^ relative to the Control where *E*_*Aa*_ was referenced to 0.0 J·mol^–1^.^165^ These reductions in the kinetic barrier correlate with net positive charge of the amino acids and the dissociation of the corresponding amine (*K*_α–*NH*_3_^+^_)) group and, thus, the abundance of the conjugate base (Figure 26).^165^ Citric acid, lacking amine groups, promoted the greatest rate-enhancing activity, thus demonstrating the ability of other functional groups to also promote nucleation rate, possibly through cooperative effects (Figure 26).^165^

**Figure 27:**
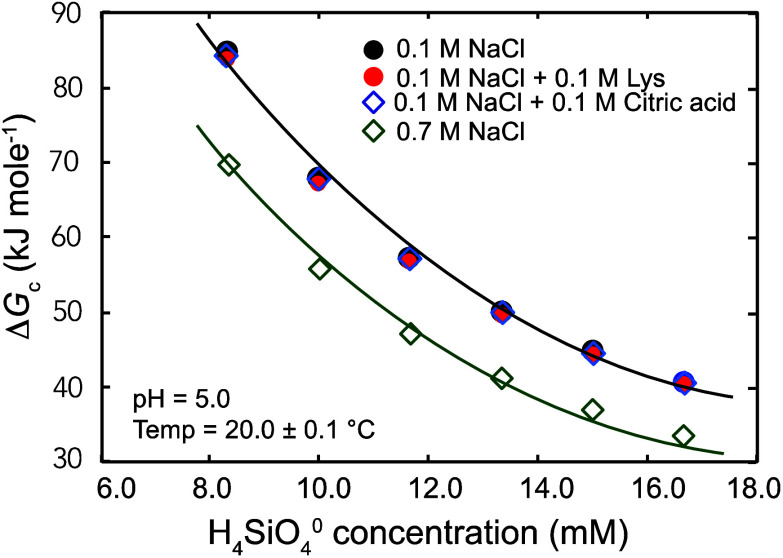
Free energy barrier to nucleation decreases with increasing supersaturation, as predicted by theory. Experimental measurements show NaCl reduces the thermodynamic barrier to nucleation independent of the organic acid in solution (after Dove et al., 2019).^165^

In contrast, electrolytes increase the rate of silicification through thermodynamic factors. They show that faster nucleation rates measured in 0.7 M NaCl solutions (compared to the 0.10 M NaCl control) are due to reductions in the thermodynamic nucleation barrier, Δ*g*_*c*_, without modifying the kinetic term (Figure 27).^165^ For example, γ_0.1 *M NaCl*_ and γ_0.7 *M NaCl*_ have values of 54.9 ± 1.6 mJ·m^–2^ and 51.4 ± 1.7 mJ·m^–2^, respectively. While an explanation of the physical basis for these distinctive thermodynamic versus kinetic-based influences on silicification rates calls for computational modeling and focused NMR studies, the findings show how rates of silicification can be tuned through additives.^165^

### 5.3 Opportunity to Use Polysaccharides for Hypothesis-Based Model Studies

In Section 3, we discussed the prevalence of polysaccharides, particularly derivatized chitin, callose, and an as-yet-unidentified phosphorylated mannan,^19^ in biosilica of major biosilicifying organisms. Despite their ubiquity, the literature has yet to quantify the energetic effects of these common macromolecules on silicification. The challenge is significant because materials present an immense array of variables that are known to influence structure–function relationships. Properties that likely influence silicification include hydrophobicity and hydrophilicity, hydrogen bonding, chain length, and possessing cationic, anionic, and zwitterionic charges via the many functional groups discussed above. Although chitin has not been shown to have a strong effect on silicification, we saw that chitosan is suggested to promote silicification (e.g., Tables 9 and 11).^162,163^ A defining feature of chitosan is the extent of deacetylation (or of exposure of amine groups which may be protonated; see Section 4.1) and presents opportunities for hypothesis-based studies using derivatives. Using well-characterized materials to explore the influence of derivatives and other macromolecule properties on function offers tremendous possibilities. Advanced synthesis and characterization along with understanding the energetics would provide clear insight into the roles of biomacromolecules on silicification, as well as a guide for materials scientists for the facile production of new biomaterials with myriad potential applications.

### 5.4 Applications of Chitosan-Silica Materials

**Figure 28:**
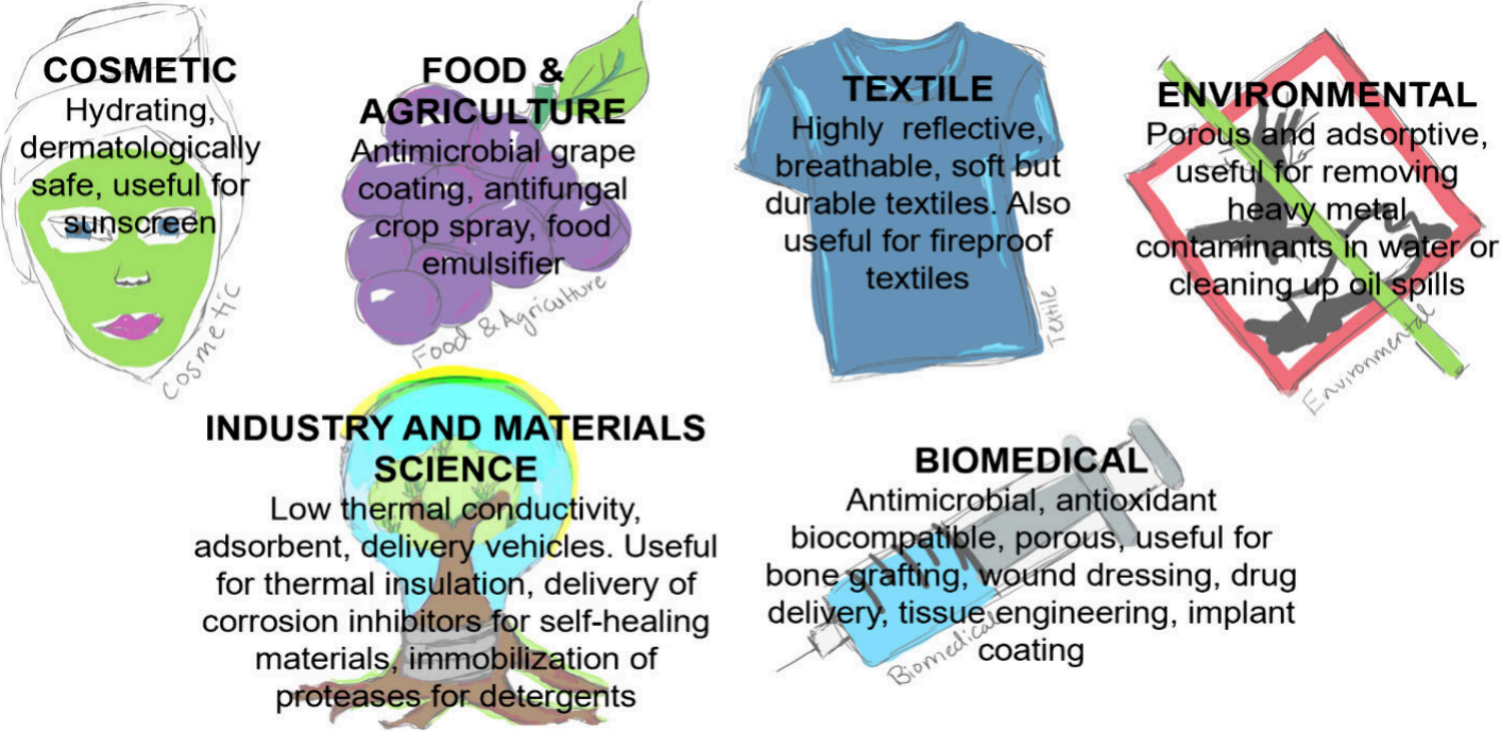
There are many applications for chitosan–silica composites. The outstanding properties of these sustainable materials hold promise for expanding their usage into new fields as we build a stronger understanding of biosilicification in molecular settings.

Silica–chitosan composite materials are finding diverse applications in a variety of industries (Figure 28). Here, we provide a glimpse of recent applications in the literature. Note that different preparation methods are used for the systems below, which emphasizes the importance of understanding the chemistry behind the preparation and deployment of silica–chitosan composites. It is also important to remember that sustainability is a factor because chitosan can be upcycled from a marine waste product and silica is an agricultural byproduct.^190^ Taken together, a common theme emerges in the many advantages these materials can offer to diverse applications with properties of biocompatibility and biodegradability, adsorbent properties, delivery mechanisms, antimicrobial properties, scaffolding, and poor conductivity.

#### 5.4.1 Cosmetic Applications

As the cosmetic industry becomes more sustainably focused, environmentally degradable and biocompatible substances, such as amorphous silica and polysaccharides, are increasingly useful. The potential of these materials will continue to increase with petrochemical-based plastics in cosmetics falling into disfavor and even being banned in some countries.^191^ Moreover, it is repeatedly shown that combined solutions of amorphous silica and a polysaccharide cause no dermal irritation in human and animal models.^192^ In addition, both polysaccharides and silica can act as carriers for small molecules and can be swelled with water, making these prime solutions for hydrating cosmetic or dermatological applications. In 2022, chitosan-coated mesoporous silica particles were found to safely contain sunscreen agents.^191^ This system repressed reactive oxygen species in solution, suggesting chitosan–silica composites are a strong alternative to plastics used in sunscreen.^191^

#### 5.4.2 Food and Agricultural Industries

Diverse applications of chitosan–silica composites are found in food and agricultural settings, from protecting plants to altering food. Antimicrobial and antioxidant protective qualities are emphasized in this industry. For example, chitosan–silica composite nanoparticles have a synergistic antifungal effect when applied to table grapes.^193^ This could provide a low-cost, safe alternative to antifungal sprays, which are increasingly ineffective due to emerging fungicide resistance.^193^ In addition, chitosan–silica nanoparticles mediate plant resistance to pests by reducing leaf oxidative stress.^194^ Alternatively, chitosan–silica nanoparticles are found to effectively perform as water–oil emulsifiers, which could be used in the food industry.^195,196^

#### 5.4.3 Textiles

Woven chitosan–silica composite textiles show excellent properties for multiple applications. They are highly reflective of specific solar wavelengths, highly flexible, breathable, and durable.^197^ The composites also exhibit a soft texture to the touch.^197^ With the textile industry producing 17 million tons of solid waste in the U.S. in 2018 alone, much of which is plastic, the construction of biodegradable fabrics is a necessity.^198^ Chitosan–silica composites as additives to cotton also enhance the properties of this traditional material with decreased flammability and decreased microbial growth.^199^ All of these properties provide new opportunities in the textile industry.

#### 5.4.4 Environmental Applications

The porosity and adsorption properties of chitosan–silica composites play a large role in their utility as environmental protection solutions. Chitosan–silica gel composites effectively remove heavy metal ions from solution for water purification.^200^ Results show this composite adsorbs ions by both physical and chemical processes.^200^ This capability could become significant for reducing pollution in urban and industrial settings. Composites could also be used for oil spill remediation as they show promise as oil adsorbents.^201^

#### 5.4.5 Biomedical

Chitosan–silica composites are perhaps the most prolific in the biomedical industry. From drug delivery to implant coating to bone grafting, there are many possible applications of this technology. Antimicrobial, biocompatibility, and small molecule delivery properties of chitosan–silica composite materials are useful features in the biomedical field. For example, chitosan–silica composites were used as biocompatible bone substitutes.^202^ In this study, the composite performed well, caused no inflammation, and promoted the growth of new bone.^202^ Different preparations of the chitosan–silica composites can produce different results in bone regeneration tissue engineering studies, suggesting how this chemistry can be tuned to optimize structure–function relationships. For example, chitosan–silica prepared by the sol–gel method can provide higher osteoconduction and cell differentiation.^203^ Chitosan–silica aerogels provided significantly more cell growth than glass when tested for tissue engineering potential.^203^ Derivatives of chitosan–silica hybrid materials have been shown to be acceptable drug carriers with added antibacterial and antioxidant properties.^204^ The antibacterial property is extremely useful when utilizing this material as a coating for implants or as a wound dressing.^205,206^ When used for cell encapsulation, osteoblasts retained over 70% viability in 168 h while antimicrobial activity inhibited the growth of *Pseudomonas aeruginosa* and *Enterococcus faecalis*.^207^

#### 5.4.6 Materials Science and Industry

Chitosan–silica materials are often used in industry for their low thermal conductivity and thermal stability, adsorbent, and delivery properties. As composites, chitosan–silica mixtures have been used to mineralize wood, to decrease water adsorption, and to increase flame-retardant properties.^208^ The thermal stability of chitosan–silica aerogels could be useful in providing thermal insulation. In one 2023 study, these composites exhibited low thermal conductivity under cryogenic and high temperature conditions, while maintaining hydrophobicity.^209^ Mesoporous chitosan–silica materials have also been used as delivery mechanisms for corrosion inhibitors.^210^ The adsorbent properties of chitosan–silica composites aided in immobilizing proteases when used in a variety of detergents.^211^ Here, the proteases maintained over 90% of their activity and their metal-based activities increased.^211^ In addition, chitosan–silica hybrid aerogels have shown promise for adsorbing thiophenes from fuels.^212^

Overall, silica–chitosan materials exhibit a wide variety of beneficial properties, which enable their use in many applications. Once the underlying chemistry of silicification onto chitosan and other polysaccharides is decoded, we can use this chemistry to facilely create tailored, detailed materials for a range of applications just as silicifying organisms produces patterned and functional materials to enhance their own life processes and survivability.

## 6 Conclusions and Future Perspectives

Despite the remarkable importance of silica–macromolecule interactions in diverse biological and synthetic systems, the mechanistic or physical basis for how macromolecules influence silicification in natural and synthetic settings has not yet been established. Many investigations are reported in the literature and provide important insights, but little quantitative understanding has been achieved regarding the ways in which macromolecules and their corresponding functional groups can be deployed to modulate silicification. Most studies are qualitative with minimal characterization of the starting material and are observational in their characterization of products.

Five major macromolecular effects have been disputed across the literature for their role in potentially promoting, inhibiting, or having no effect on silica formation. First, many studies claim that cations are a major promoter of silicification, while others suggest that cations have negligible to inhibitory effects on silicification in comparison to other macromolecular effects. Second, anionic effects have been disputed for their ability to promote or inhibit silica formation. Third, cooperative interactions between organic anions and cations promote silicification under certain conditions but inhibit silicification in others. The literature is also contradictory with regard to the two effects of hydrophobicity and finally hydrogen bonding on silica formation. The disparate experimental and analytical methods, dissimilar solution compositions (i.e., wide-ranging pH values), wide ranges of supersaturations, and varied precursor molecules contribute to the difficulty in comparing conclusions across the literature.

Looking ahead, there are now opportunities to address these shortcomings by combining advances in macromolecule synthesis and characterization with hypothesis-based approaches to experimental design and interpretation. Through quantitative approaches, we can finally discern the physical basis of (bio)silicification and build a shared mechanistic understanding of how organic molecules modulate silicification. These experiments can become the first steps toward a general understanding of how organic additives provide facile control over silica formation. Such a construct will inform diverse natural and synthetic silica systems and provide highly functional materials under environmentally friendly conditions.

## References

[ref1] Dove P. M.; De Yoreo J. J.; Weiner S. Biomineralization; Rev. Mineral. Geochem., 2003; Vol. 54.

[ref2] Baüerlein E. Biomineralization: Progress in Biology, Molecular Biology and Application; Wiley-VCH, 2005; 10.1002/3527604138.

[ref3] Simkiss K.; Wilbur K. M. Biomineralization: Cell Biology and Mineral Deposition; Academic Press Inc., 1989.

[ref4] Lowenstam H. A.; Weiner S. On Biomineralization; Oxford University Press, 1989.

[ref5] Mann S. Biomineralization: Principles and Concepts in Bioinorganic Materials Chemistry; Oxford University Press, 2001.

[ref6] van der Wal P.; de Jong E. W.; Westbroek P.; de Bruijn W. C.; Mulder-Stapel A. A. Ultrastructural Polysaccharide Localization in Calcifying and Naked Cells of the Coccolithophorid Emiliania Huxleyi. Protoplasma 1983, 118, 157–168. 10.1007/BF01293073.

[ref7] Kröger N.; Wetherbee R. Pleuralins Are Involved in Theca Differentiation in the Diatom Cylindrotheca Fusiformis. Protist 2000, 151 (3), 263–273. 10.1078/1434-4610-00024. 11079771

[ref8] Perry C. C.; Belton D.; Shafran K. Studies of Biosilicas; Structural Aspects, Chemical Principles, Model Studies and the Future. Prog. Mol. Subcell. Biol. 2003, 33, 269–299. 10.1007/978-3-642-55486-5_11. 14518377

[ref9] Young J. R.; Henriksen K. Biomineralization Within Vesicles: The Calcite of Coccoliths. Rev. Mineral. Geochem. 2003, 54 (1), 189–215. 10.2113/0540189.

[ref10] Levi-Kalisman Y.; Falini G.; Addadi L.; Weiner S. Structure of the Nacreous Organic Matrix of a Bivalve Mollusk Shell Examined in the Hydrated State Using Cryo-TEM. J. Struct. Biol. 2001, 135 (1), 8–17. 10.1006/jsbi.2001.4372. 11562161

[ref11] Weiner S.; Dove P. M. An Overview of Biomineralization Processes and the Problem of the Vital Effect. Rev. Mineral. Geochem. 2003, 54 (1), 1–29. 10.2113/0540001.

[ref12] Kotzsch A.; Pawolski D.; Milentyev A.; Shevchenko A.; Scheffel A.; Poulsen N.; Shevchenko A.; Kröger N. Biochemical Composition and Assembly of Biosilica-Associated Insoluble Organic Matrices from the Diatom Thalassiosira Pseudonana. J. Biol. Chem. 2016, 291 (10), 4982–4997. 10.1074/jbc.M115.706440. 26710847 PMC4777836

[ref13] Conley D. J.; Frings P. J.; Fontorbe G.; Clymans W.; Stadmark J.; Hendry K. R.; Marron A. O.; De La Rocha C. L. Biosilicification Drives a Decline of Dissolved Si in the Oceans through Geologic Time. Front. Mar. Sci. 2017, 4, 1–19. 10.3389/fmars.2017.00397.

[ref14] Knoll A. H. Biomineralization and Evolutionary History. Rev. Mineral. Geochem. 2003, 54 (1), 329–356. 10.2113/0540329.

[ref15] Prado Figueroa M. D. The Growth of Chalcedony (Nanocrystalline Silica) in Electric Organs from Living Marine Fish. In Advances in Crystallization Processes; 2012; pp 285–300.

[ref16] Prado Figueroa M.; Flores L.; Sanchez J.; Cesaretti N. Biosilicification (Chalcedony) in Human Cerebral Cortex, Hippocampus and Cerebellum from Aged Patients. Micron 2008, 39 (7), 859–867. 10.1016/j.micron.2007.12.005. 18243715

[ref17] Currie H. A.; Perry C. C. Silica in Plants: Biological, Biochemical and Chemical Studies. Ann. Bot. 2007, 100 (7), 1383–1389. 10.1093/aob/mcm247. 17921489 PMC2759229

[ref18] Ehrlich H.; Luczak M.; Ziganshin R.; Mikšík I.; Wysokowski M.; Simon P.; Baranowska-Bosiacka I.; Kupnicka P.; Ereskovsky A.; Galli R.; Dyshlovoy S.; Fischer J.; Tabachnick K. R.; Petrenko I.; Jesionowski T.; Lubkowska A.; Figlerowicz M.; Ivanenko V. N.; Summers A. P. Arrested in Glass: Actin within Sophisticated Architectures of Biosilica in Sponges. Advanced Science 2022, 9 (11), 2105059 10.1002/advs.202105059. 35156333 PMC9009123

[ref19] Falciatore A.; Mock T. The Molecular Life of Diatoms; Springer, 2022. 10.1016/j.margen.2014.07.002 25027953

[ref20] Ikeda T. Bacterial Biosilicification: A New Insight into the Global Silicon Cycle. Biosci., Biotechnol., Biochem. 2021, 85 (6), 1324–1331. 10.1093/bbb/zbab069. 33877302

[ref21] Afanasieva M. S.; Amon E. O. Biomineralization of Radiolarian Skeletons. Paleontol. J. 2014, 48, 1473–1486. 10.1134/S0031030114140020.

[ref22] Wysokowski M.; Jesionowski T.; Ehrlich H. Biosilica as a Source for Inspiration in Biological Materials Science. Am. Mineral. 2018, 103 (5), 665–691. 10.2138/am-2018-6429.

[ref23] Matsunaga S.; Sakai R.; Jimbo M.; Kamiya H. Long-Chain Polyamines (LCPAs) from Marine Sponge: Possible Implication in Spicule Formation. ChemBioChem. 2007, 8 (14), 1729–1735. 10.1002/cbic.200700305. 17683052

[ref24] Gregor B. Silica Balance of the Ocean. Nature 1968, 219, 360–361. 10.1038/219360b0.

[ref25] Trujillo A. P.; Thurman H. V. Essentials of Oceanography, 12th ed.; Pearson, 2016.

[ref26] Mesa M.; Becerra N. Y. Silica/Protein and Silica/Polysaccharide Interactions and Their Contributions to the Functional Properties of Derived Hybrid Wound Dressing Hydrogels. Int. J. of Biomater. 2021, 2021, 1–13. 10.1155/2021/6857204. PMC8580642 34777502

[ref27] Hernández-González A. C.; Téllez-Jurado L.; Rodríguez-Lorenzob L. M. SYNTHESIS OF IN-SITU SILICA-ALGINATE HYBRID HYDROGELS BY A SOL-GEL ROUTE. Carbohydr. Polym. 2020, 250, 116877 10.1016/j.carbpol.2020.116877. 33049876

[ref28] Shchipunov Y. A.; Karpenko T. Y.; Bakunina I. Y.; Burtseva Y. V.; Zvyagintseva T. N. A New Precursor for the Immobilization of Enzymes inside Sol-Gel-Derived Hybrid Silica Nanocomposites Containing Polysaccharides. J. Biochem. Biophys. Methods 2004, 58 (1), 25–38. 10.1016/S0165-022X(03)00108-8. 14597186

[ref29] Fu J.; Wang S.; He C.; Lu Z.; Huang J.; Chen Z. Facilitated Fabrication of High Strength Silica Aerogels Using Cellulose Nanofibrils as Scaffold. Carbohydr. Polym. 2016, 147, 89–96. 10.1016/j.carbpol.2016.03.048. 27178912

[ref30] Curley R.; Holmes J. D.; Flynn E. J. Can Sustainable, Monodisperse, Spherical Silica Be Produced from Biomolecules? A Review. Appl. Nanosci. 2021, 11, 1777–1804. 10.1007/s13204-021-01869-6.

[ref31] Annenkov V. V.; Danilovtseva E. N.; Pal’shin V. A.; Verkhozina O. N.; Zelinskiy S. N.; Krishnan U. M. Silicic Acid Condensation under the Influence of Water-Soluble Polymers: From Biology to New Materials. RSC Adv. 2017, 7, 20995–21027. 10.1039/C7RA01310H.

[ref32] Iler R. K. The Chemistry of Silica: Solubility, Polymerization, Colloid and Surface Properties and Biochemistry; Wiley, 1979.

[ref33] Belton D. J.; Deschaume O.; Perry C. C. An Overview of the Fundamentals of the Chemistry of Silica with Relevance to Biosilicification and Technological Advances. FEBS J. 2012, 279 (10), 1710–1720. 10.1111/j.1742-4658.2012.08531.x. 22333209 PMC3334419

[ref34] Hildebrand M.; Lerch S. J. L.; Shrestha R. P. Understanding Diatom Cell Wall Silicification-Moving Forward. Front. Mar. Sci. 2018, 5, 1 10.3389/fmars.2018.00125.29552559

[ref35] Schröder H. C.; Wang X.; Tremel W.; Ushijima H.; Müller W. E. G. Biofabrication of Biosilica-Glass by Living Organisms. Nat. Prod. Rep. 2008, 25, 455–474. 10.1039/b612515h. 18497895

[ref36] Sánchez-Téllez D. A.; Rodríguez-Lorenzo L. M.; Téllez-Jurado L. Siloxane-Inorganic Chemical Crosslinking of Hyaluronic Acid - Based Hybrid Hydrogels: Structural Characterization. Carbohydr. Polym. 2020, 230, 115590 10.1016/j.carbpol.2019.115590. 31887936

[ref37] Patwardhan S. V.; Clarson S. J. Silicification and Biosilicification: The Role of Macromolecules in Bioinspired Silica Synthesis. Ph.D. Dissertation, University of Cincinnati, Cincinnati, OH, 2003.

[ref38] Rimstidt J. D. Silica: A Geochemist’s Perspective. In The Physics and Chemistry of SiO2 and the Si-SiO2 Interface; Electrochemical Society, Inc., 2000; Vol. 4, pp 216–234.

[ref39] Yang J.; Mccormick A. V. Thermochemistry of Silicate Speciation in Aqueous Sodium Silicate Solutions: Ionization and Polymerization of Small Silicate Ion; Office of Naval Research, 1993.

[ref40] Jantschke A.; Spinde K.; Brunner E. Electrostatic Interplay: The Interaction Triangle of Polyamines, Silicic Acid, and Phosphate Studied through Turbidity Measurements, Silicomolybdic Acid Test, and 29Si NMR Spectroscopy. Beilstein J. Nanotechnol. 2014, 5 (1), 2026–2035. 10.3762/bjnano.5.211. 25551030 PMC4273220

[ref41] Iler R. K. Isolation and Characterization of Particle Nuclei during the Polymerization of Silicic Acid to Colloidal Silica. J. Colloid Interface Sci. 1980, 75 (1), 138–148. 10.1016/0021-9797(80)90357-4.

[ref42] Takahashi M.; Abe Y.; Tanaka M. Elucidation of Molybdosilicate Complexes in the Molybdate Yellow Method by ESI-MS. Talanta 2015, 131, 301–308. 10.1016/j.talanta.2014.07.079. 25281106

[ref43] Canabady-Rochelle L. L. S.; Belton D. J.; Deschaume O.; Currie H. A.; Kaplan D. L.; Perry C. C. Bioinspired Silicification of Silica-Binding Peptide-Silk Protein Chimeras: Comparison of Chemically and Genetically Produced Proteins. Biomacromolecules 2012, 13 (3), 683–690. 10.1021/bm201555c. 22229696 PMC3304446

[ref44] Shimono T.; Isobe T.; Tarutani T. Study of the Polymerization of Silicic Acid in Aqueous Solution by Trimethylsilylation—Gas Chromatography. J. Chromatogr. A 1983, 258, 73–80. 10.1016/S0021-9673(00)96399-4.

[ref45] Tarutani T. Polymerization of Silicic Acid A Review. Anal. Sci. 1989, 5, 245–252. 10.2116/analsci.5.245.

[ref46] Bussian P.; Sobott F.; Brutschy B.; Schrader W.; Schüth F. Speciation in Solution: Silicate Oligomers in Aqueous Solutions Detected by Mass Spectrometry. Angew. Chem., Int. Ed. 2000, 39, 3901–3905. 10.1002/1521-3773(20001103)39:21<3901::AID-ANIE3901>3.0.CO;2-D. 29711690

[ref47] Tanaka M.; Takahashi K. Characterization of Silica Dissolved in Sodium Chloride Solution Using Fast Atom Bombardment Mass Spectrometry. J. Mass Spectrom. 2000, 35 (7), 853–859. 10.1002/1096-9888(200007)35:7<853::AID-JMS9>3.0.CO;2-R. 10934438

[ref48] Benhelal E.; Oliver T. K.; Farhang F.; Hook J. M.; Rayson M. S.; Brent G. F.; Stockenhuber M.; Kennedy E. M. Structure of Silica Polymers and Reaction Mechanism for Formation of Silica-Rich Precipitated Phases in Direct Aqueous Carbon Mineralization. Ind. Eng. Chem. Res. 2020, 59 (15), 6828–6839. 10.1021/acs.iecr.9b04379.

[ref49] Belton D. J.; Patwardhan S. V.; Annenkov V. V.; Danilovtseva E. N.; Perry C. C. From Biosilicification to Tailored Materials: Optimizing Hydrophobic Domains and Resistance to Protonation of Polyamines. Proc. Natl. Acad. Sci. U. S. A. 2008, 105 (16), 5963–5968. 10.1073/pnas.0710809105. 18420819 PMC2329702

[ref50] Radulović S.; Sunkara S.; Rachel R.; Leitinger G. Three-Dimensional SEM, TEM, and STEM for Analysis of Large-Scale Biological Systems. Histochem. Cell. Biol. 2022, 158, 203–211. 10.1007/s00418-022-02117-w. 35829815 PMC9399040

[ref51] Wallace A. F.; De Yoreo J. J.; Dove P. M. Kinetics of Silica Nucleation on Carboxyl- and Amine-Terminated Surfaces: Insights for Biomineralization. J. Am. Chem. Soc. 2009, 131 (14), 5244–5250. 10.1021/ja809486b. 19301812

[ref52] Venugopal A.; Ruiz-Perez L.; Swamynathan K.; Kulkarni C.; Calò A.; Kumar M. Caught in Action: Visualizing Dynamic Nanostructures within Supramolecular Systems Chemistry. Angew. Chem., Int. Ed. 2023, 62 (8), e202208681 10.1002/anie.202208681. 36469792

[ref53] Zerda T. W.; Artaki I.; Jonas J. Study of Polymerization Processes in Acid and Base Catalyzed Silica Sol-Gels. J. Non Cryst. Solids 1986, 81 (3), 365–379. 10.1016/0022-3093(86)90503-X.

[ref54] Levitt M. H. Spin Dynamics: Basics of Nuclear Magnetic Resonance, 2^nd^ ed.; Wiley, 2008.

[ref55] Meinhold R. H.; Rothbaum H. P.; Newman R. H. Polymerization of Supersaturated Silica Solutions Monitored by Silicon-29 Nuclear Magnetic Resonance. J. Colloid Interface Sci. 1985, 108 (1), 234–236. 10.1016/0021-9797(85)90255-3.

[ref56] Montagna M.; Brückner S. I.; Dianat A.; Gutierrez R.; Daus F.; Geyer A.; Brunner E.; Cuniberti G. Interactions of Long-Chain Polyamines with Silica Studied by Molecular Dynamics Simulations and Solid-State NMR Spectroscopy. Langmuir 2020, 36 (39), 11600–11609. 10.1021/acs.langmuir.0c02157. 32924496

[ref57] Bravo-Flores I.; Meléndez-Zamudio M.; Guerra-Contreras A.; Ramírez-Oliva E.; Álvarez-Guzmán G.; Zárraga-Núñez R.; Villegas A.; Cervantes J. Revisiting the System Silanes-Polysaccharides: The Cases of THEOS-Chitosan and MeTHEOS-Chitosan. Macromol. Rapid Commun. 2021, 42 (5), 2000612 10.1002/marc.202000612. 33458894

[ref58] Eckert H.; Montagna M.; Dianat A.; Gutierrez R.; Bobeth M.; Cuniberti G. Exploring the Organic-Inorganic Interface in Biosilica: Atomistic Modeling of Polyamine and Silica Precursors Aggregation Behavior. BMC Mater. 2020, 2, 6 10.1186/s42833-020-00012-z.

[ref59] Armin G.; Inomura K. Modeling the Elemental Stoichiometry and Silica Accumulation in Diatoms. Curr. Res. Microb. Sci. 2022, 3, 100164 10.1016/j.crmicr.2022.100164. 36518164 PMC9743000

[ref60] Maher S.; Kumeria T.; Aw M. S.; Losic D. Diatom Silica for Biomedical Applications: Recent Progress and Advances. Adv. Healthcare Mater. 2018, 7 (19), 1800552 10.1002/adhm.201800552. 30118185

[ref61] Yamanaka S.; Yano R.; Usami H.; Hayashida N.; Ohguchi M.; Takeda H.; Yoshino K. Optical Properties of Diatom Silica Frustule with Special Reference to Blue Light. J. Appl. Phys. 2008, 103 (7), 74701 10.1063/1.2903342.

[ref62] Reimann B. E. F. Deposition of Silica inside a Diatom Cell. Exp. Cell. Res. 1964, 34 (3), 605–608. 10.1016/0014-4827(64)90248-4. 14170019

[ref63] Drum R. W.; Pankratz H. S. Post Mitotic Fine Structure of Gomphonema Parvulum. J. Ultrastruct. Res. 1964, 10 (3–4), 217–223. 10.1016/S0022-5320(64)80006-X. 14166290

[ref64] Mayzel B.; Aram L.; Varsano N.; Wolf S. G.; Gal A. Structural Evidence for Extracellular Silica Formation by Diatoms. Nat. Commun. 2021, 12 (1), 1–8. 10.1038/s41467-021-24944-6. 34330922 PMC8324917

[ref65] Demadis K. D.; Pachis K.; Ketsetzi A.; Stathoulopoulou A. Bioinspired Control of Colloidal Silica in Vitro by Dual Polymeric Assemblies of Zwitterionic Phosphomethylated Chitosan and Polycations or Polyanions. Adv. Colloid Interface Sci. 2009, 151 (1–2), 33–48. 10.1016/j.cis.2009.07.005. 19691946

[ref66] Hildebrand M.; Lerch S. J. L.; Shrestha R. P. Understanding Diatom Cell Wall Silicification-Moving Forward. Front. Mar. Sci. 2018, 5, 125 10.3389/fmars.2018.00125.

[ref67] Kröger N.; Deutzmann R.; Sumper M. Polycationic Peptides from Diatom Biosilica That Direct Silica Nanosphere Formation. Science 1999, 286 (5442), 1129–1132. 10.1126/science.286.5442.1129. 10550045

[ref68] Kröger N.; Lorenz S.; Brunner E.; Sumper M. Self-Assembly of Highly Phosphorylated Silaffins and Their Function in Biosilica Morphogenesis. Science 2002, 298 (5593), 584–586. 10.1126/science.1076221. 12386330

[ref69] Kumar S.; Natalio F.; Elbaum R. Protein-Driven Biomineralization: Comparing Silica Formation in Grass Silica Cells to Other Biomineralization Processes. J. Struct. Biol. 2021, 213 (1), 107665 10.1016/j.jsb.2020.107665. 33227416

[ref70] Ehren H. L.; Kolbe F.; Lucini Paioni A.; Brunner E.; Baldus M. DNP-Supported Solid-State NMR Studies of 13C,15N,29Si-Enriched Biosilica of Cyclotella Cryptica and Thalassiosira Pseudonana. Discovery Mater. 2021, 1, 9 10.1007/s43939-021-00009-9.

[ref71] Bernecker A.; Wieneke R.; Riedel R.; Seibt M.; Geyer A.; Steinem C. Tailored Synthetic Polyamines for Controlled Biomimetic Silica Formation. J. Am. Chem. Soc. 2010, 132 (3), 1023–1031. 10.1021/ja9061163. 20041715

[ref72] Jantschke A.; Koers E.; Mance D.; Weingarth M.; Brunner E.; Baldus M. Insight into the Supramolecular Architecture of Intact Diatom Biosilica from DNP-Supported Solid-State NMR Spectroscopy. Angew. Chem., Int. Ed. 2015, 54 (50), 15069–15073. 10.1002/anie.201507327. 26509491

[ref73] Knight B. M.; Edgar K. J.; De Yoreo J. J.; Dove P. M. Chitosan as a Canvas for Studies of Macromolecular Controls on CaCO3 Biological Crystallization. Biomacromolecules 2023, 24 (3), 1078–1102. 10.1021/acs.biomac.2c01394. 36853173

[ref74] Ehrlich H. Chitin and Collagen as Universal and Alternative Templates in Biomineralization. Int. Geol. Rev. 2010, 52 (7–8), 661–699. 10.1080/00206811003679521.

[ref75] Kolbe F.; Ehren H. L.; Kohrs S.; Butscher D.; Reiß L.; Baldus M.; Brunner E. Solid-State NMR Spectroscopic Studies of 13C,15N,29Si-Enriched Biosilica from the Marine Diatom Cyclotella Cryptica. Discovery Mater. 2021, 1, 3 10.1007/s43939-020-00003-7.

[ref76] Morin L. G.; Smucker R. A.; Herth W. Effects of Two Chitin Synthesis Inhibitors on Thalassiosira Fluviatilis and Cyclotella Cryptica. FEMS Microbiol. Lett. 1986, 37 (3), 263–268. 10.1111/j.1574-6968.1986.tb01806.x.

[ref77] Wustmann M.; Poulsen N.; Kröger N.; van Pée K. H. Chitin Synthase Localization in the Diatom Thalassiosira Pseudonana. BMC Mater. 2020, 2, 10 10.1186/s42833-020-00016-9.

[ref78] Hedrich R.; MacHill S.; Brunner E. Biomineralization in Diatoms-Phosphorylated Saccharides are part of Stephanopyxis Turris Biosilica. Carbohydr. Res. 2013, 365, 52–60. 10.1016/j.carres.2012.11.001. 23220060

[ref79] Le Costaouëc T.; Unamunzaga C.; Mantecon L.; Helbert W. New Structural Insights into the Cell-Wall Polysaccharide of the Diatom Phaeodactylum Tricornutum. Algal Res. 2017, 26, 172–179. 10.1016/j.algal.2017.07.021.

[ref80] Maldonado M.; López-Acosta M.; Sitjà C.; García-Puig M.; Galobart C.; Ercilla G.; Leynaert A. Sponge Skeletons as an Important Sink of Silicon in the Global Oceans. Nat. Geosci. 2019, 12, 815–822. 10.1038/s41561-019-0430-7.

[ref81] Cassarino L.; Coath C. D.; Xavier J. R.; Hendry K. R. Silicon Isotopes of Deep Sea Sponges: New Insights into Biomineralisation and Skeletal Structure. Biogeosciences 2018, 15 (22), 6959–6977. 10.5194/bg-15-6959-2018.

[ref82] Maldonado M.; López-Acosta M.; Beazley L.; Kenchington E.; Koutsouveli V.; Riesgo A. Cooperation between Passive and Active Silicon Transporters Clarifies the Ecophysiology and Evolution of Biosilicification in Sponges. Sci. Adv. 2020, 6 (28), 1–14. 10.1126/sciadv.aba9322. PMC7439455 32832609

[ref83] Ehrlich H.; Krautter M.; Hanke T.; Simon P.; Knieb C.; Heinemann S.; Worch H. First Evidence of the Presence of Chitin in Skeletons of Marine Sponges. Part II. Glass Sponges (Hexactinellida: Porifera). J. Exp. Zool., Part B 2007, 308B (4), 473–483. 10.1002/jez.b.21174. 17520693

[ref84] Wiens M.; Bausen M.; Natalio F.; Link T.; Schlossmacher U.; Müller W. E. G. The Role of the Silicatein-α Interactor Silintaphin-1 in Biomimetic Biomineralization. Biomaterials 2009, 30 (8), 1648–1656. 10.1016/j.biomaterials.2008.12.021. 19118892

[ref85] Shimizu K.; Amano T.; Bari M. R.; Weaver J. C.; Arima J.; Mori N. Glassin, a Histidine-Rich Protein from the Siliceous Skeletal System of the Marine Sponge Euplectella, Directs Silica Polycondensation. Proc. Natl. Acad. Sci. U. S. A. 2015, 112 (37), 11449–11454. 10.1073/pnas.1506968112. 26261346 PMC4577155

[ref86] Nishi M.; Kobayashi H.; Amano T.; Sakate Y.; Bito T.; Arima J.; Shimizu K. Identification of the Domains Involved in Promotion of Silica Formation in Glassin, a Protein Occluded in Hexactinellid Sponge Biosilica, for Development of a Tag for Purification and Immobilization of Recombinant Proteins. Mar. Biotechnol. 2020, 22, 739–747. 10.1007/s10126-020-09967-2. 32291549

[ref87] Guerriero G.; Hausman J. F.; Legay S. Silicon and the Plant Extracellular Matrix. Front. Plant Sci. 2016, 7, 1 10.3389/fpls.2016.00463. 27148294 PMC4828433

[ref88] Neethirajan S.; Gordon R.; Wang L. Potential of Silica Bodies (Phytoliths) for Nanotechnology. Trends Biotechnol. 2009, 27 (8), 461–467. 10.1016/j.tibtech.2009.05.002. 19577814

[ref89] Yoshida S.; Ohnishi Y.; Kitagishi K. Chemical Forms, Mobility and Deposition of Silicon in Rice Plant. Soil Sci. Plant Nutr. 1962, 8 (3), 15–21. 10.1080/00380768.1962.10430992.

[ref90] Keeping M. G.; Kvedaras O. L.; Bruton A. G. Epidermal Silicon in Sugarcane: Cultivar Differences and Role in Resistance to Sugarcane Borer Eldana Saccharina. Environ. Exp. Bot. 2009, 66 (1), 54–60. 10.1016/j.envexpbot.2008.12.012.

[ref91] Jones L. H. P.; Milne A. A.; Wadham S. M. Studies of Silica in The Oat Plant: II. Distribution of the Silica in the Plant. Plant Soil 1963, 18, 358–371. 10.1007/BF01347235.

[ref92] Whittenberger R. T. Silicon Absorption by Rye and Sunflower. Am. J. Bot. 1945, 32 (9), 539–549. 10.1002/j.1537-2197.1945.tb05156.x.

[ref93] Heine G.; Tikum G.; Horst W. J. Silicon Nutrition of Tomato and Bitter Gourd with Special Emphasis on Silicon Distribution in Root Fractions. J. Plant Nutr. Soil Sci. 2005, 168 (4), 600–606. 10.1002/jpln.200420508.

[ref94] Exley C. A Possible Mechanism of Biological Silicification in Plants. Front Plant Sci. 2015, 6, 1 10.3389/fpls.2015.00853. 26500676 PMC4598575

[ref95] Ma J. F.; Goto S.; Tamai K.; Ichii M. Role of Root Hairs and Lateral Roots in Silicon Uptake by Rice. Plant Physiol. 2001, 127 (4), 1773–1780. 10.1104/pp.010271. 11743120 PMC133580

[ref96] Mandlik R.; Thakral V.; Raturi G.; Shinde S.; Nikolić M.; Tripathi D. K.; Sonah H.; Deshmukh R. Significance of Silicon Uptake, Transport, and Deposition in Plants. J. Exp. Bot. 2020, 71 (21), 6703–6718. 10.1093/jxb/eraa301. 32592476

[ref97] Swain R.; Rout G. R. Silicon in Agriculture. Sustainable Agriculture Reviews 2017, 25, 233–260. 10.1007/978-3-319-58679-3_8.

[ref98] de Tombeur F.; Raven J. A.; Toussaint A.; Lambers H.; Cooke J.; Hartley S. E.; Johnson S. N.; Coq S.; Katz O.; Schaller J.; Violle C. Why Do Plants Silicify?. Trends Ecol. Evol. 2023, 38 (3), 275–288. 10.1016/j.tree.2022.11.002. 36428125

[ref99] Wang L.; Nie Q.; Li M.; Zhang F.; Zhuang J.; Yang W.; Li T.; Wang Y. Biosilicified Structures for Cooling Plant Leaves: A Mechanism of Highly Efficient Midinfrared Thermal Emission. Appl. Phys. Lett. 2005, 87 (19), 194105 10.1063/1.2126115.

[ref100] Zexer N.; Kumar S.; Elbaum R. Silica Deposition in Plants: Scaffolding the Mineralization. Ann. Bot. 2023, 131 (6), 897–908. 10.1093/aob/mcad056. 37094329 PMC10332400

[ref101] Preari M.; Spinde K.; Lazic J.; Brunner E.; Demadis K. D. Bioinspired Insights into Silicic Acid Stabilization Mechanisms: The Dominant Role of Polyethylene Glycol-Induced Hydrogen Bonding. J. Am. Chem. Soc. 2014, 136 (11), 4236–4244. 10.1021/ja411822s. 24564240

[ref102] Kumar S.; Adiram-Filiba N.; Blum S.; Sanchez-Lopez J. A.; Tzfadia O.; Omid A.; Volpin H.; Heifetz Y.; Goobes G.; Elbaum R. Siliplant1 Protein Precipitates Silica in Sorghum Silica Cells. J. Exp. Bot. 2020, 71 (21), 6830–6843. 10.1093/jxb/eraa258. 32485738

[ref103] Kulich I.; Vojtíková Z.; Sabol P.; Ortmannová J.; Neděla V.; Tihlaříková E.; Zárský V. Exocyst Subunit EXO70H4 Has a Specific Role in Callose Synthase Secretion and Silica Accumulation. Plant Physiol. 2018, 176 (3), 2040–2051. 10.1104/pp.17.01693. 29301954 PMC5841730

[ref104] Guerriero G.; Stokes I.; Exley C. Is Callose Required for Silicification in Plants?. Biol. Lett. 2018, 14 (10), 20180338 10.1098/rsbl.2018.0338. 30282746 PMC6227863

[ref105] Kido N.; Yokoyama R.; Yamamoto T.; Furukawa J.; Iwai H.; Satoh S.; Nishitani K. The Matrix Polysaccharide (1;3,1;4)-β-d-Glucan Is Involved in Silicon-Dependent Strengthening of Rice Cell Wall. Plant Cell Physiol. 2015, 56 (2), 268–276. 10.1093/pcp/pcu162. 25392067

[ref106] Adiram-Filiba N.; Geiger Y.; Kumar S.; Keinan-Adamsky K.; Elbaum R.; Goobes G. Peptides from Diatoms and Grasses Harness Phosphate Ion Binding to Silica to Help Regulate Biomaterial Structure. Acta Biomater. 2020, 112, 286–297. 10.1016/j.actbio.2020.05.006. 32434074

[ref107] Dickerson M. B.; Sandhage K. H.; Naik R. R. Protein- and Peptide-Directed Syntheses of Inorganic Materials. Chem. Rev. 2008, 108 (11), 4935–4978. 10.1021/cr8002328. 18973389

[ref108] Chen C.-L.; Rosi N. L. Peptide-Based Methods for the Preparation of Nanostructured Inorganic Materials. Angew. Chem., Int. Ed. 2010, 49 (11), 1924–1942. 10.1002/anie.200903572. 20183835

[ref109] Cha J. N.; Shimizu K.; Zhou Y.; Christiansen S. C.; Chmelka B. F.; Stucky G. D.; Morse D. E. Silicatein Filaments and Subunits from a Marine Sponge Direct the polymerization of Silica and Silicones in Vitro. Proc. Natl. Acad. Sci. U. S. A. 1999, 96 (2), 361 10.1073/pnas.96.2.361. 9892638 PMC15141

[ref110] Brzezinski M. A.; Nelson D. M. A Solvent Extraction Method for the Colorimetric Determination of Nanomolar Concentrations of Silicic Acid in Seawater. Mar Chem. 1986, 19 (2), 139–151. 10.1016/0304-4203(86)90045-9.

[ref111] Zhou Y.; Shimizu K.; Cha J. N.; Stucky G. D.; Morse D. E. Efficient Catalysis of Polysiloxane Synthesis by Silicatein a Requires Specific Hydroxy and Imidazole Functionalities. Angew. Chem., Int. Ed. 1999, 38 (6), 779–782. 10.1002/(SICI)1521-3773(19990315)38:6<779::AID-ANIE779>3.0.CO;2-#. 29711800

[ref112] Povarova N. V.; Barinov N. A.; Baranov M. S.; Markina N. M.; Varizhuk A. M.; Pozmogova G. E.; Klinov D. V.; Kozhemyako V. B.; Lukyanov K. A. Efficient Silica Synthesis from Tetra(Glycerol)Orthosilicate with Cathepsin-and Silicatein-like Proteins. Sci. Rep. 2018, 8, 16759 10.1038/s41598-018-34965-9. 30425281 PMC6233156

[ref113] Strunge K.; Hoinkis N.; Lutz H.; Alamdari S.; Roeters S. J.; Lu H.; Pfaendtner J.; Weidner T. Peptide Mimic of the Marine Sponge Protein Silicatein Fabricates Ultrathin Nanosheets of Silicon Dioxide and Titanium Dioxide. Langmuir 2022, 38 (26), 8087–8093. 10.1021/acs.langmuir.2c00918. 35727216

[ref114] Shkryl Y. N.; Bulgakov V. P.; Veremeichik G. N.; Kovalchuk S. N.; Kozhemyako V. B.; Kamenev D. G.; Semiletova I. v.; Timofeeva Y. O.; Shchipunov Y. A.; Kulchin Y. N. Bioinspired Enzymatic Synthesis of Silica Nanocrystals Provided by Recombinant Silicatein from the Marine Sponge Latrunculia Oparinae. Bioprocess Biosyst. En.g 2016, 39, 53–58. 10.1007/s00449-015-1488-2. 26494639

[ref115] Rai A.; Perry C. C. Facile Fabrication of Uniform Silica Films with Tunable Physical Properties Using Silicatein Protein from Sponges. Langmuir 2010, 26 (6), 4152–4159. 10.1021/la903366a. 20000795

[ref116] Sumerel J. L.; Yang W.; Kisailus D.; Weaver J. C.; Choi J. H.; Morse D. E. Biocatalytically Templated Synthesis of Titanium Dioxide. Chem. Mater. 2003, 15 (25), 4804–4809. 10.1021/cm030254u.

[ref117] Kröger N.; Deutzmann R.; Sumper M. Polycationic Peptides from Diatom Biosilica That Direct Silica Nanosphere Formation. Science 1999, 286 (5442), 1129–1132. 10.1126/science.286.5442.1129. 10550045

[ref118] Kamalov M.; Hajradini A.; Rentenberger C.; Becker C. F. W. N-Terminal Residues of Silaffin Peptides Impact Morphology of Biomimetic Silica Particles. Mater. Lett. 2018, 212, 114–117. 10.1016/j.matlet.2017.10.050.

[ref119] Brott L. L.; Naik R. R.; Pikas D. J.; Kirkpatrick S. M.; Tomlin D. W.; Whitlock P. W.; Clarson S. J.; Stone M. O. Ultrafast Holographic Nanopatterning of Biocatalytically Formed Silica. Nature 2001, 413, 291–293. 10.1038/35095031. 11565027

[ref120] Spinthaki A.; Zerfaß C.; Paulsen H.; Hobe S.; Demadis K. D. Pleiotropic Role of Recombinant Silaffin-Like Cationic Polypeptide P5S3: Peptide-Induced Silicic Acid Stabilization, Silica Formation and Inhibition of Silica Dissolution. ChemistrySelect 2017, 2 (1), 6–17. 10.1002/slct.201601086.

[ref121] Wenzl S.; Hett R.; Richthammer P.; Sumper M. Silacidins: Highly Acidic Phosphopeptides from Diatom Shells Assist in Silica Precipitation In Vitro. Angew. Chem., Int. Ed. 2008, 47 (9), 1729–1732. 10.1002/anie.200704994. 18203228

[ref122] Kauss H.; Seehaus K.; Franke R.; Gilbert S.; Dietrich R. A.; Kröger N. Silica Deposition by a Strongly Cationic Proline-Rich Protein from Systemically Resistant Cucumber Plants. Plant J. 2003, 33 (1), 87–95. 10.1046/j.1365-313X.2003.01606.x. 12943543

[ref123] Baker P. J.; Patwardhan S. v; Numata K. Synthesis of Homopolypeptides by Aminolysis Mediated by Proteases Encapsulated in Silica Nanospheres. Macromol. Biosci. 2014, 14 (11), 1619–1626. 10.1002/mabi.201400295. 25154484

[ref124] Gautier C.; Abdoul-Aribi N.; Roux C.; Lopez P. J.; Livage J.; Coradin T. Biomimetic Dual Templating of Silica by Polysaccharide/Protein Assemblies. Colloids Surf., B 2008, 65 (1), 140–145. 10.1016/j.colsurfb.2008.03.005. 18440789

[ref125] Tilburey G. E.; Patwardhan S. V.; Huang J.; Kaplan D. L.; Perry C. C. Are Hydroxyl-Containing Biomolecules Important in Biosilicification? A Model Study. J. Phys. Chem. B 2007, 111 (17), 4630–4638. 10.1021/jp068539s. 17408253

[ref126] Altunbas A.; Sharma N.; Lamm M. S.; Yan C.; Nagarkar R. P.; Schneider J. P.; Pochan D. J. Peptide-Silica Hybrid Networks: Biomimetic Control of Network Mechanical Behavior. ACS Nano 2010, 4 (1), 181–188. 10.1021/nn901226h. 20028097 PMC3488860

[ref127] Harrison C. C.; Loton N. Novel Routes to Designer Silicas: Studies of the Decomposition of (M^+^)_2_ [Si(C_6_H_4_O_2_)_3_]xH_2_O. Importance of M^+^ Identity of the Kinetics of Oligomerisation and the Structural Characteristics of the Silicas Produced. J. Chem. Soc., Faraday Trans. 1995, 91, 4287–4297. 10.1039/FT9959104287.

[ref128] Yang W.; Yin Q.; Chen C.-L. Designing Sequence-Defined Peptoids for Biomimetic Control over Inorganic Crystallization. Chem. Mater. 2021, 33 (9), 3047–3065. 10.1021/acs.chemmater.1c00243.

[ref129] Cai B.; Li Z.; Chen C.-L. Programming Amphiphilic Peptoid Oligomers for Hierarchical Assembly and Inorganic Crystallization. Acc. Chem. Res. 2021, 54 (1), 81–91. 10.1021/acs.accounts.0c00533. 33136361

[ref130] Yang W.; Cai B.; Lachowski K. J.; Yin Q.; De Yoreo J. J.; Pozzo L. D.; Chen C.-L. Insights into the Biomimetic Synthesis of 2D ZnO Nanomaterials through Peptoid Engineering. J. Phys. Chem. Lett. 2023, 14 (43), 9732–9739. 10.1021/acs.jpclett.3c01882. 37882440

[ref131] Yang W.; Zhou Y.; Jin B.; Qi X.; Cai B.; Yin Q.; Pfaendtner J.; De Yoreo J. J.; Chen C.-L. Designing Sequence-Defined Peptoids for Fibrillar Self-Assembly and Silicification. J. Colloid Interface Sci. 2023, 634, 450–459. 10.1016/j.jcis.2022.11.136. 36542974

[ref132] Torkelson K.; Naser N. Y.; Qi X.; Li Z.; Yang W.; Pushpavanam K.; Chen C.-L.; Baneyx F.; Pfaendtner J. Rational Design of Novel Biomimetic Sequence-Defined Polymers for Mineralization Applications. Chem. Mater. 2024, 36 (2), 786–794. 10.1021/acs.chemmater.3c02216.

[ref133] Shao L.; Ma J.; Prelesnik J. L.; Zhou Y.; Nguyen M.; Zhao M.; Jenekhe S. A.; Kalinin S. V.; Ferguson A. L.; Pfaendtner J.; Mundy C. J.; De Yoreo J. J.; Baneyx F.; Chen C.-L. Hierarchical Materials from High Information Content Macromolecular Building Blocks: Construction, Dynamic Interventions, and Prediction. Chem. Rev. 2022, 122 (24), 17397–17478. 10.1021/acs.chemrev.2c00220. 36260695

[ref134] Li Z.; Cai B.; Yang W.; Chen C.-L. Hierarchical Nanomaterials Assembled from Peptoids and Other Sequence-Defined Synthetic Polymers. Chem. Rev. 2021, 121 (22), 14031–14087. 10.1021/acs.chemrev.1c00024. 34342989

[ref135] Lau K. H. A. Peptoids for Biomaterials Science. Biomater. Sci. 2014, 2, 627–633. 10.1039/C3BM60269A. 32481842

[ref136] Chen C.-L.; Qi J.; Zuckermann R. N.; De Yoreo J. J. Engineered Biomimetic Polymers as Tunable Agents for Controlling CaCO3 mineralization. J. Am. Chem. Soc. 2011, 133 (14), 5214–5217. 10.1021/ja200595f. 21417474

[ref137] Chen C.-L.; Qi J.; Tao J.; Zuckermann R. N.; De Yoreo J. J. Tuning Calcite Morphology and Growth Acceleration by a Rational Design of Highly Stable Protein-Mimetics. Sci. Rep. 2014, 4, 6266 10.1038/srep06266. 25189418 PMC5385837

[ref138] Ma J.; Jin B.; Guye K. N.; Chowdhury M. E.; Naser N. Y.; Chen C.-L.; De Yoreo J. J.; Baneyx F. Controlling Mineralization with Protein-Functionalized Peptoid Nanotubes. Adv. Mater. 2023, 35 (3), 2207543 10.1002/adma.202207543. 36281797

[ref139] Chien Y. C.; Tao J.; Saeki K.; Chin A. F.; Lau J. L.; Chen C.-L.; Zuckermann R. N.; Marshall S. J.; Marshall G. W.; De Yoreo J. J. Using Biomimetic Polymers in Place of Noncollagenous Proteins to Achieve Functional Remineralization of Dentin Tissues. ACS Biomater. Sci. Eng. 2017, 3 (12), 3469–3479. 10.1021/acsbiomaterials.7b00378. 29479561 PMC5821432

[ref140] Yan F.; Liu L.; Walsh T. R.; Gong Y.; El-Khoury P. Z.; Zhang Y.; Zhu Z.; De Yoreo J. J.; Engelhard M. H.; Zhang X.; Chen C.-L. Controlled Synthesis of Highly-Branched Plasmonic Gold Nanoparticles through Peptoid Engineering. Nat. Commun. 2018, 9, 2327 10.1038/s41467-018-04789-2. 29899378 PMC5998043

[ref141] Jin B.; Yan F.; Qi X.; Cai B.; Tao J.; Fu X.; Tan S.; Zhang P.; Pfaendtner J.; Naser N. Y.; Baneyx F.; Zhang X.; De Yoreo J. J.; Chen C.-L. Peptoid-Directed Formation of Five-Fold Twinned Au Nanostars through Particle Attachment and Facet Stabilization. Angew. Chem., Int. Ed. 2022, 61 (14), e202201980 10.1002/anie.202201980. PMC9258440 35167709

[ref142] Qi X.; Jin B.; Cai B.; Yan F.; De Yoreo J.; Chen C.-L.; Pfaendtner J. Molecular Driving Force for Facet Selectivity of Sequence-Defined Amphiphilic Peptoids at Au-Water Interfaces. J. Phys. Chem. B 2022, 126 (27), 5117–5126. 10.1021/acs.jpcb.2c02638. 35763341

[ref143] Calkins A. L.; Yin J.; Rangel J. L.; Landry M. R.; Fuller A. A.; Stokes G. Y. A Thermodynamic Description of the Adsorption of Simple Water-Soluble Peptoids to Silica. Langmuir 2016, 32 (44), 11690–11697. 10.1021/acs.langmuir.6b02804. 27756123

[ref144] Abdelhamid M. A. A.; Pack S. P. Biomimetic and Bioinspired Silicifications: Recent Advances for Biomaterial Design and Applications. Acta Biomater. 2021, 120, 38–56. 10.1016/j.actbio.2020.05.017. 32447061

[ref145] Sumper M.; Lorenz S.; Brunner Biomimetic Control of Size in the Polyamine-Directed Formation of Silica Nanospheres. Angew. Chem., Int. Ed. 2003, 42 (42), 5192–5195. 10.1002/anie.200352212. 14601169

[ref146] Kröger N.; Deutzmann R.; Bergsdorf C.; Sumper M. Species-Specific Polyamines from Diatoms Control Silica Morphology. Proc. Natl. Acad. Sci. U.S.A. 2000, 97 (26), 14133–14138. 10.1073/pnas.260496497. 11106386 PMC18883

[ref147] Patwardhan S. V.; Tilburey G. E.; Perry C. C. Interactions of Amines with Silicon Species in Undersaturated Solutions Leads to Dissolution and/or Precipitation of Silica. Langmuir 2011, 27 (24), 15135–15145. 10.1021/la204180r. 22085267

[ref148] Manning J. R. H.; Yip T. W. S.; Centi A.; Jorge M.; Patwardhan S. V. An Eco-Friendly, Tunable and Scalable Method for Producing Porous Functional Nanomaterials Designed Using Molecular Interactions. ChemSusChem 2017, 10 (8), 1683–1691. 10.1002/cssc.201700027. 28235156 PMC5434938

[ref149] Le Chatelier H. On a General Statement of the Laws of Chemical Equilibrium. C. R. Acad. Sci. Paris 1884, 786–789.

[ref150] Gügi B.; Le Costaouec T.; Burel C.; Lerouge P.; Helbert W.; Bardor M. Diatom-Specific Oligosaccharide and Polysaccharide Structures Help to Unravel Biosynthetic Capabilities in Diatoms. Mar. Drugs 2015, 13 (9), 5993–6018. 10.3390/md13095993. 26393622 PMC4584364

[ref151] Shchipunov Y. A.; Karpenko T. Y. Hybrid Polysaccharide-Silica Nanocomposites Prepared by the Sol-Gel Technique. Langmuir 2004, 20 (10), 3882–3887. 10.1021/la0356912. 15969374

[ref152] Shchipunov Y. A.; Burtseva Y. V.; Karpenko T. Y.; Shevchenko N. M.; Zvyagintseva T. N. Highly Efficient Immobilization of Endo-1,3-d-Glucanases (Laminarinases) from Marine Mollusks in Novel Hybrid Polysaccharide-Silica Nanocomposites with Regulated Composition. J. Mol. Catal. B. Enzym. 2006, 40 (1–2), 16–23. 10.1016/j.molcatb.2006.02.002.

[ref153] Shchipunov Y. A. Sol-Gel-Derived Biomaterials of Silica and Carrageenans. J. Colloid Interface Sci. 2003, 268 (1), 68–76. 10.1016/S0021-9797(03)00457-0. 14611774

[ref154] Shchipunov Y. A.; Karpenko T. Y.; Krekoten A. V.; Postnova I. V. Gelling of Otherwise Nongelable Polysaccharides. J. Colloid Interface Sci. 2005, 287, 373–378. 10.1016/j.jcis.2005.02.004. 15925600

[ref155] Witoon T.; Chareonpanich M. Interaction of Chitosan with Tetraethyl Orthosilicate on the Formation of Silica Nanoparticles: Effect of PH and Chitosan Concentration. Ceram. Int. 2012, 38 (7), 5999–6007. 10.1016/j.ceramint.2012.04.056.

[ref156] Witoon T.; Tepsarn S.; Kittipokin P.; Embley B.; Chareonpanich M. Effect of PH and Chitosan Concentration on Precipitation and Morphology of Hierarchical Porous Silica. J. Non. Cryst. Solids 2011, 357 (19–20), 3513–3519. 10.1016/j.jnoncrysol.2011.06.029.

[ref157] Chang J. S.; Kong Z. L.; Hwang D. F.; Chang K. L. B. Chitosan-Catalyzed Aggregation during the Biomimetic Synthesis of Silica Nanoparticles. Chem. Mater. 2006, 18 (3), 1714 10.1021/cm0603553.

[ref158] Witoon T.; Chareonpanich M.; Limtrakul J. Effect of Acidity on the Formation of Silica-Chitosan Hybrid Materials and Thermal Conductive Property. J. Solgel Sci. Technol. 2009, 51, 146–152. 10.1007/s10971-009-1986-2.

[ref159] Leng B.; Shao Z.; Bomans P. H. H.; Brylka L. J.; Sommerdijk N. A. J. M.; De With G.; Ming W. Cryogenic Electron Tomography Reveals the Template Effect of Chitosan in Biomimetic Silicification. Chem. Commun. 2010, 46, 1703–1705. 10.1039/b922670b. 20177622

[ref160] Roberts G. A. F. Chitin Chemistry; Macmillan Education UK, 1992.

[ref161] Coradin T.; Livage J. Synthesis and Characterization of Alginate/Silica Biocomposites. J. SolGel Sci. Technol. 2003, 26, 1165–1168. 10.1023/A:1020787514512.

[ref162] Spinde K.; Kammer M.; Freyer K.; Ehrlich H.; Vournakis J. N.; Brunner E. Biomimetic Silicification of Fibrous Chitin from Diatoms. Chem. Mater. 2011, 23 (11), 2973–2978. 10.1021/cm200677d.

[ref163] Wysokowski M.; Behm T.; Born R.; Bazhenov V. V.; Meißner H.; Richter G.; Szwarc-Rzepka K.; Makarova A.; Vyalikh D.; Schupp P.; Jesionowski T.; Ehrlich H. Preparation of Chitin-Silica Composites by in Vitro Silicification of Two-Dimensional Ianthella Basta Demosponge Chitinous Scaffolds under Modified Stöber Conditions. Mater. Sci. Eng., C 2013, 33 (7), 3935–3941. 10.1016/j.msec.2013.05.030. 23910299

[ref164] Wang G. H.; Zhang L. M. Manipulating Formation and Drug-Release Behavior of New Sol-Gel Silica Matrix by Hydroxypropyl Guar Gum. J. Phys. Chem. B 2007, 111 (36), 10665–10670. 10.1021/jp070370a. 17711328

[ref165] Dove P. M.; Han N.; Wallace A. F. Systematic Dependence of Kinetic and Thermodynamic Barriers to Homogeneous Silica Nucleation on NaCl and Amino Acids. J. Mater. Res. 2019, 34, 442–455. 10.1557/jmr.2018.474.

[ref166] Kinrade S. D.; Hamilton R. J.; Schach A. S.; Knight C. T. G. Aqueous Hypervalent Silicon Complexes with Aliphatic Sugar Acids. J. Chem. Soc., Dalton Trans. 2001, 961–963. 10.1039/b010111g.

[ref167] Fang J. Y.; Ma X. L. In Vitro Simulation Studies of Silica Deposition Induced by Lignin from Rice. J. Zhejiang Univ.-Sci. B 2006, 7, 267 10.1631/jzus.2006.B0267. 16532527 PMC1447512

[ref168] Wang G.; Wang H. J.; Zhou H.; Nian Q. G.; Song Z.; Deng Y. Q.; Wang X.; Zhu S. Y.; Li X. F.; Qin C. F.; Tang R. Hydrated Silica Exterior Produced by Biomimetic Silicification Confers Viral Vaccine Heat-Resistance. ACS Nano 2015, 9 (1), 799–808. 10.1021/nn5063276. 25574563

[ref169] Brückner S. I.; Donets S.; Dianat A.; Bobeth M.; Gutiérrez R.; Cuniberti G.; Brunner E. Probing Silica-Biomolecule Interactions by Solid-State NMR and Molecular Dynamics Simulations. Langmuir 2016, 32 (44), 11698–11705. 10.1021/acs.langmuir.6b03311. 27759396

[ref170] Spinthaki A.; Skordalou G.; Stathoulopoulou A.; Demadis K. D. Modified Macromolecules in the Prevention of Silica Scale. Pure Appl. Chem. 2016, 88 (10–11), 1037–1047. 10.1515/pac-2016-0807.

[ref171] Korhatzis E.; Xanthopoulos K.; Demadis K. D. Polymeric Silica Scale Inhibitors: Computational Predictions and Experimental Studies on Inhibitor Efficiencies. In AMPP Annual Conference + Expo 2022, San Antonio, TX.

[ref172] Wang S.; Lin P. A.; DeLuca M.; Zauscher S.; Arya G.; Ke Y. Controlling Silicification on DNA Origami with Polynucleotide Brushes. J. Am. Chem. Soc. 2024, 146 (1), 358–367. 10.1021/jacs.3c09310. 38117542 PMC10785815

[ref173] Wassermann L. M.; Scheckenbach M.; Baptist A. V.; Glembockyte V.; Heuer-Jungemann A. Full Site-Specific Addressability in DNA Origami-Templated Silica Nanostructures. Adv. Mater. 2023, 35 (23), 2212024 10.1002/adma.202212024. 36932052

[ref174] Wang H.; Li Z.; Liu X.; Jia S.; Gao Y.; Li M. Rapid Silicification of a DNA Origami with Shape Fidelity. ACS Appl. Bio. Mater. 2024, 7 (4), 2511–2518. 10.1021/acsabm.4c00124. 38512069

[ref175] Cao Y.; Xie J.; Liu B.; Han L.; Che S. Synthesis and Characterization of Multi-Helical DNA-Silica Fibers. Chem. Commun. 2013, 49, 1097–1099. 10.1039/c2cc37470f. 23282743

[ref176] Nielsen A. R.; Jelavić S.; Murray D.; Rad B.; Andersson M. P.; Ceccato M.; Mitchell A. C.; Stipp S. L. S.; Zuckermann R. N.; Sand K. K. Thermodynamic and Kinetic Parameters for Calcite Nucleation on Peptoid and Model Scaffolds: A Step toward Nacre Mimicry. Cryst. Growth. Des. 2020, 20 (6), 3762–3771. 10.1021/acs.cgd.0c00029. PMC7660692 33192182

[ref177] Hamm L. M.; Giuffre A. J.; Han N.; Tao J.; Wang D.; De Yoreo J. J.; Dove P. M. Reconciling Disparate Views of Template-Directed Nucleation through Measurement of Calcite Nucleation Kinetics and Binding Energies. Proc. Natl. Acad. Sci. U. S. A. 2014, 111 (4), 1304–1309. 10.1073/pnas.1312369111. 24434555 PMC3910584

[ref178] Demadis K. D.; Ketsetzi A.; Pachis K.; Ramos V. M. Inhibitory Effects of Multicomponent, Phosphonate-Grafted, Zwitterionic Chitosan Biomacromolecules on Silicic Acid Condensation. Biomacromolecules 2008, 9 (11), 3288–3293. 10.1021/bm800872n. 18947251

[ref179] Brückner S. I.; Donets S.; Dianat A.; Bobeth M.; Gutiérrez R.; Cuniberti G.; Brunner E. Probing Silica-Biomolecule Interactions by Solid-State NMR and Molecular Dynamics Simulations. Langmuir 2016, 32 (44), 11698–11705. 10.1021/acs.langmuir.6b03311. 27759396

[ref180] Wysokowski M.; Piasecki A.; Bazhenov V. V.; Paukszta D.; Born R.; Schupp P.; Petrenko I.; Jesionowski T. Poriferan Chitin as the Scaffold for Nanosilica Deposition under Hydrothermal Synthesis Conditions. Journal of Chitin and Chitosan Science 2013, 1 (1), 26–33. 10.1166/jcc.2013.1009.

[ref181] Makrides A. C.; Turner M.; Slaughter J. Condensation of Silica from Supersaturated Silicic Acid Solutions. J. Colloid Interface Sci. 1980, 73 (2), 345–367. 10.1016/0021-9797(80)90081-8.

[ref182] Gibbs J. W. On the equilibrium of heterogeneous substances. Science 1878, S3–16 (96), 441–458. 10.2475/ajs.s3-16.96.441.

[ref183] Teychené S.; Biscans B. Nucleation Kinetics of Polymorphs: Induction Period and Interfacial Energy Measurements. Cryst. Growth Des. 2008, 8 (4), 1133–1139. 10.1021/cg0609320.

[ref184] Söhnel O.; Mullin J. W. Interpretation of Crystallization Induction Periods. J. Colloid Interface Sci. 1988, 123 (1), 43–50. 10.1016/0021-9797(88)90219-6.

[ref185] Chernov A. A. Modern Crystallography III; Springer-Verlag, 1984.

[ref186] De Yoreo J. J.; Sommerdijk N. A. J. M.; Dove P. M. Nucleation Pathways in Electrolyte Solutions. In New Perspectives on Mineral Nucleation and Growth; Springer: Cham, 2017; pp 1–24; 10.1007/978-3-319-45669-0_1.

[ref187] Karthika S.; Radhakrishnan T. K.; Kalaichelvi P. A Review of Classical and Nonclassical Nucleation Theories. Cryst. Growth Des. 2016, 16 (11), 6663–6681. 10.1021/acs.cgd.6b00794.

[ref188] Gunnarsson I.; Arnórsson S. Amorphous Silica Solubility and the Thermodynamic Properties of H4SiO°4 in the Range of 0° to 350°C at Psat. Geochim. Cosmochim. Acta 2000, 64 (13), 2295–2307. 10.1016/S0016-7037(99)00426-3.

[ref189] Becker R.; Döring W. Kinetische Behandlung Der Keimbildung in Übersättigten Dämpfen. Ann. Phys. 1935, 416 (8), 719–752. 10.1002/andp.19354160806.

[ref190] Aswathi V. P.; Meera S.; Maria C. G. A.; Nidhin M. Green Synthesis of Nanoparticles from Biodegradable Waste Extracts and Their Applications: A Critical Review. Nanotechnol. Environ. Eng. 2023, 8, 377–397. 10.1007/s41204-022-00276-8.

[ref191] Choi S.; Na H.; Rahman R. T.; Sim J.; Chang J. B.; Nam Y. S. Chitosan-Coated Mesoporous Silica Particles as a Plastic-Free Platform for Photochemical Suppression and Stabilization of Organic Ultraviolet Filters. J. Photochem. Photobiol., B 2022, 235, 112565 10.1016/j.jphotobiol.2022.112565. 36113261

[ref192] Bergfeld W. F.; Belsito D. V.; Hill R. A.; Klaassen C. D.; Liebler D. C.; Marks J. G.; Shank R. C.; Slaga T. J.; Snyder P. W.; Heldreth B. Amended Safety Assessment of Synthetically-Manufactured Amorphous Silica and Hydrated Silica as Used in Cosmetics; Cosmetic Ingredient Review: Washington, DC, 2019; https://cir-safety.org/sites/default/files/silica092019FR.pdf (accessed 2024–07–17). 10.1177/10915818251356416 40932354

[ref193] Youssef K.; de Oliveira A. G.; Tischer C. A.; Hussain I.; Roberto S. R. Synergistic Effect of a Novel Chitosan/Silica Nanocomposites-Based Formulation against Gray Mold of Table Grapes and Its Possible Mode of Action. Int. J. Biol. Macromol. 2019, 141, 247–258. 10.1016/j.ijbiomac.2019.08.249. 31476398

[ref194] Ji H.; Wang J.; Xue A.; Chen F.; Guo H.; Xiao Z.; Wang Z. Chitosan-Silica Nanocomposites Induced Resistance in Faba Bean Plants against Aphids (Acyrthosiphon Pisum). Environ. Sci. Nano 2023, 10 (8), 1966–1977. 10.1039/D3EN00234A.

[ref195] Alison L.; Demirörs A. F.; Tervoort E.; Teleki A.; Vermant J.; Studart A. R. Emulsions Stabilized by Chitosan-Modified Silica Nanoparticles: PH Control of Structure-Property Relations. Langmuir 2018, 34 (21), 6147–6160. 10.1021/acs.langmuir.8b00622. 29719151

[ref196] Heidari F.; Jafari S. M.; Ziaiifar A. M.; Anton N. Surface Modification of Silica Nanoparticles by Chitosan for Stabilization of Water-in-Oil Pickering Emulsions. Carbohydr. Polym. Technol. Appl. 2023, 6, 100381 10.1016/j.carpta.2023.100381.

[ref197] Chen C.; Jia X.; Li X.; Shi M.; Hu J.; Song M.; Wu S.; Dai H.; Wang X.; Geng H. Scalable Wet-Spinning of Wearable Chitosan-Silica Textile for All-Day Radiative Cooling. Chem. Eng. J. 2023, 475, 146307 10.1016/j.cej.2023.146307.

[ref198] US EPA. Textiles: Material-Specific Data; https://www.epa.gov/facts-and-figures-about-materials-waste-and-recycling/textiles-material-specific-data (accessed 2023–10–22).

[ref199] Szadkowski B.; Piotrowska M.; Rybiński P.; Marzec A. Natural Bioactive Formulations for Biodegradable Cotton Eco-Fabrics with Antimicrobial and Fire-Shielding Properties. Int. J. Biol. Macromol. 2023, 237, 124143 10.1016/j.ijbiomac.2023.124143. 36972831

[ref200] Joshi S.; Srivastava R. K. Adsorptive Removal of Lead (Pb), Copper (Cu), Nickel (Ni) and Mercury (Hg) Ions from Water Using Chitosan Silica Gel Composite. Environ. Monit. Assess. 2019, 191, 615 10.1007/s10661-019-7777-5. 31493036

[ref201] Saharan Y.; Singh J.; Goyat R.; Umar A.; Algadi H.; Ibrahim A. A.; Kumar R.; Baskoutas S. Nanoporous and Hydrophobic New Chitosan-Silica Blend Aerogels for Enhanced Oil Adsorption Capacity. J. Clean. Prod. 2022, 351, 131247 10.1016/j.jclepro.2022.131247.

[ref202] Alvarez Echazú M. I.; Renou S. J.; Alvarez G. S.; Desimone M. F.; Olmedo D. G. Synthesis and Evaluation of a Chitosan-Silica-Based Bone Substitute for Tissue Engineering. Int. J. Mol. Sci. 2022, 23 (21), 13379 10.3390/ijms232113379. 36362167 PMC9657383

[ref203] Pérez-Moreno A.; Piñero M.; Fernández-Montesinos R.; Pinaglia-Tobaruela G.; Reyes-Peces M. V.; Mesa-Díaz M. d. M.; Vilches-Pérez J. I.; Esquivias L.; de la Rosa-Fox N.; Salido M. Chitosan-Silica Hybrid Biomaterials for Bone Tissue Engineering: A Comparative Study of Xerogels and Aerogels. Gels 2023, 9 (5), 383 10.3390/gels9050383. 37232975 PMC10217634

[ref204] Kumari R.; Narvi S. S.; Dutta P. K. Thiol Modified Chitosan-Silica Nanohybrid for Antibacterial, Antioxidant and Drug Delivery Application. J. Indian Chem. Soc. 2021, 98 (8), 100108 10.1016/j.jics.2021.100108.

[ref205] Becerra N. Y.; Restrepo L. M.; Galeano Y.; Tobón A. C.; Turizo L. F.; Mesa M. Improving Fibrin Hydrogels’ Mechanical Properties, through Addition of Silica or Chitosan-Silica Materials, for Potential Application as Wound Dressings. Int. J. Biomater. 2021, 2021, 1 10.1155/2021/9933331. PMC8192204 34188685

[ref206] Gouveia Z.; Perinpanayagam H.; Zhu J. Development of Robust Chitosan-Silica Class II Hybrid Coatings with Antimicrobial Properties for Titanium Implants. Coatings 2020, 10 (6), 534 10.3390/coatings10060534.

[ref207] Jayash S. N.; Cooper P. R.; Shelton R. M.; Kuehne S. A.; Poologasundarampillai G. Novel Chitosan-Silica Hybrid Hydrogels for Cell Encapsulation and Drug Delivery. Int. J. Mol. Sci. 2021, 22 (22), 12267 10.3390/ijms222212267. 34830145 PMC8624171

[ref208] Li H.; Wang C.; Yang T.; Wang Z.; Xia M.; Zhang M.; Liu D.; Yuan G. Mineralizing Wood with Chitosan-Silica to Enhance the Flame Retardant and Physical-Mechanical Properties. J. Solgel Sci. Technol. 2023, 107, 57–67. 10.1007/s10971-022-05730-2.

[ref209] Zhang S.; Liao Y.; Lu K.; Wang Z.; Wang J.; Lai L.; Xin W.; Xiao Y.; Xiong S.; Ding F. Chitosan/Silica Hybrid Aerogels with Synergistic Capability for Superior Hydrophobicity and Mechanical Robustness. Carbohydr. Polym. 2023, 320, 121245 10.1016/j.carbpol.2023.121245. 37659825

[ref210] Zhang C.; Li W.; Guo Z.; Sun T.; Wang W.; Chen S. Controllable Construction of Mesoporous Silica/2D-COF Nanocomposites Reinforced Epoxy Coatings with Excellent Self-Repairing and Long-Lasting Anticorrosion Performances. Prog. Org. Coat. 2023, 177, 107441 10.1016/j.porgcoat.2023.107441.

[ref211] Karakurt V.; Samsa C. G. Immobilization of Protease on Chitosan-Silica Gel Beads for High Detergent and Surfactant Stability and High Tolerance against Metallic Ions and Organic Solvents. Chem. Pap. 2023, 77 (6), 3361–3372. 10.1007/s11696-023-02709-3.

[ref212] Xu J.; Teng X.; Wen Z.; Zhang B.; Tao W.; Zhang Z.; Ning W. High Adsorptive Performance of SiO2@Chitosan Oligosaccharide Hybrid Aerogels for Thiophenics in Model Fuels and Aromatic Products. J. Solgel Sci. Technol. 2023, 108, 862–877. 10.1007/s10971-023-06234-3.

[ref213] Wilhelm S.; Kind M. Influence of PH, Temperature and Sample Size on Natural and Enforced Syneresis of Precipitated Silica. Polymers 2015, 7 (12), 2504–2521. 10.3390/polym7121528.

[ref214] Zgłobicka I.; Gluch J.; Liao Z.; Werner S.; Guttmann P.; Li Q.; Bazarnik P.; Plocinski T.; Witkowski A.; Kurzydlowski K. J. Insight into Diatom Frustule Structures Using Various Imaging Techniques. Sci. Rep. 2021, 11, 14555 10.1038/s41598-021-94069-9. 34267299 PMC8282634

[ref215] Kröger N.; Deutzmann R.; Sumper M. Silica-Precipitating Peptides from Diatoms: The Chemical Structure of Silaffin-1a from Cylindrotheca Fusiformis. J. Biol. Chem. 2001, 276 (28), 26066–26070. 10.1074/jbc.M102093200. 11349130

[ref216] Müller W. E. G.; Rothenberger M.; Boreiko A.; Tremel W.; Reiber A.; Schröder H. C. Formation of Siliceous Spicules in the Marine Demosponge Suberites Domuncula. Cell Tissue Res. 2005, 321, 285–297. 10.1007/s00441-005-1141-5. 15947968

